# DAF-16/FOXO requires Protein Phosphatase 4 to initiate transcription of stress resistance and longevity promoting genes

**DOI:** 10.1038/s41467-019-13931-7

**Published:** 2020-01-09

**Authors:** Ilke Sen, Xin Zhou, Alexey Chernobrovkin, Nataly Puerta-Cavanzo, Takaharu Kanno, Jérôme Salignon, Andrea Stoehr, Xin-Xuan Lin, Bora Baskaner, Simone Brandenburg, Camilla Björkegren, Roman A. Zubarev, Christian G. Riedel

**Affiliations:** 1 Integrated Cardio Metabolic Centre (ICMC), Department of Medicine, Karolinska Institute, Blickagången 6, 14157 Huddinge, Sweden; 2 Department of Biosciences and Nutrition, Karolinska Institute, Blickagången 16, 14157 Huddinge, Sweden; 3 European Research Institute for the Biology of Ageing (ERIBA), University Medical Center Groningen (UMCG), University of Groningen, Antonius Deusinglaan, 1, 9713AV Groningen, The Netherlands; 4 Department of Medical Biochemistry and Biophysics, Karolinska Institute, Solnavägen 9, 17165 Solna, Sweden; 5 Department of Cellular and Molecular Biology, Karolinska Institute, Solnavägen 9, 17165 Solna, Sweden

**Keywords:** Transcription, Ageing

## Abstract

In *C. elegans,* the conserved transcription factor DAF-16/FOXO is a powerful aging regulator, relaying dire conditions into expression of stress resistance and longevity promoting genes. For some of these functions, including low insulin/IGF signaling (IIS), DAF-16 depends on the protein SMK-1/SMEK, but how SMK-1 exerts this role has remained unknown. We show that SMK-1 functions as part of a specific Protein Phosphatase 4 complex (PP4^SMK-1^). Loss of PP4^SMK-1^ hinders transcriptional initiation at several DAF-16-activated genes, predominantly by impairing RNA polymerase II recruitment to their promoters. Search for the relevant substrate of PP4^SMK-1^ by phosphoproteomics identified the conserved transcriptional regulator SPT-5/SUPT5H, whose knockdown phenocopies the loss of PP4^SMK-1^. Phosphoregulation of SPT-5 is known to control transcriptional events such as elongation and termination. Here we also show that transcription initiating events are influenced by the phosphorylation status of SPT-5, particularly at DAF-16 target genes where transcriptional initiation appears rate limiting, rendering PP4^SMK-1^ crucial for many of DAF-16’s physiological roles.

## Abstract (web summary)

The transcription factor DAF-16/FOXO mediates a wide variety of aging-preventive responses by driving the expression of stress resistance and longevity promoting genes. Here the authors show that transcriptional initiation at many DAF-16/FOXO target genes requires the dephosphorylation of SPT-5 by Protein Phosphatase 4.

## Introduction

Insulin/IGF-like signaling (IIS) is one of the most prominent aging-regulatory pathways. Conserved from worms to mammals^1,2^, it has critical roles in the regulation of metabolism, reproduction, stress resistance, and longevity^3,4^. In particular the stress resistance and longevity phenotypes are controlled through a conserved downstream transcription factor called DAF-16/FOXO. Under normal conditions, IIS is active and phosphorylates DAF-16 via AKT and SGK kinases, leading to the sequestration of DAF-16 in the cytoplasm through interaction with 14-3-3, away from its target genes. However, under dire conditions—those leading to low IIS in particular—phosphorylation is reduced, allowing DAF-16 to translocate into the nucleus. There it binds to the promoters and regulates transcription of its target genes, predominantly inducing genes that contribute to stress response mechanisms and promote longevity^5,6^.

Although nuclear translocation of DAF-16 is a prerequisite, it is not sufficient for DAF-16 to regulate its target genes^7^. Additional regulators and cofactors are needed. One of the most powerful positive regulators of DAF-16, the conserved protein SMK-1/SMEK, was described already a decade ago^8^. Under low IIS, SMK-1 is required for DAF-16-driven longevity and the resistance to oxidative stress, to ultraviolet (UV) irradiation, and to pathogens. Interestingly though, it is not required for DAF-16 to promote thermotolerance, to promote dauer formation, or to delay reproduction^8^. This suggests that SMK-1 genetically interacts with DAF-16 but regulates only parts of its functions, likely by influencing the expression of only a specific subset of its target genes.

Despite this prior work and the interesting phenotypes observed, the mechanism by which SMK-1 would influence gene expression has remained unknown. Here we show that SMK-1 acts as part of a specific Protein Phosphatase 4 complex, PP4^SMK-1^, which binds and dephosphorylates the transcriptional regulator SPT-5. Under low IIS, this dephosphorylation of SPT-5 promotes transcription initiating events, in particular RNA Pol II (Pol II) recruitment  at several DAF-16 target genes, and thereby promotes a large part of DAF-16’s functions.

## Results

### SMK-1 is part of a nuclear Protein Phosphatase 4 complex

**Fig. 1:**
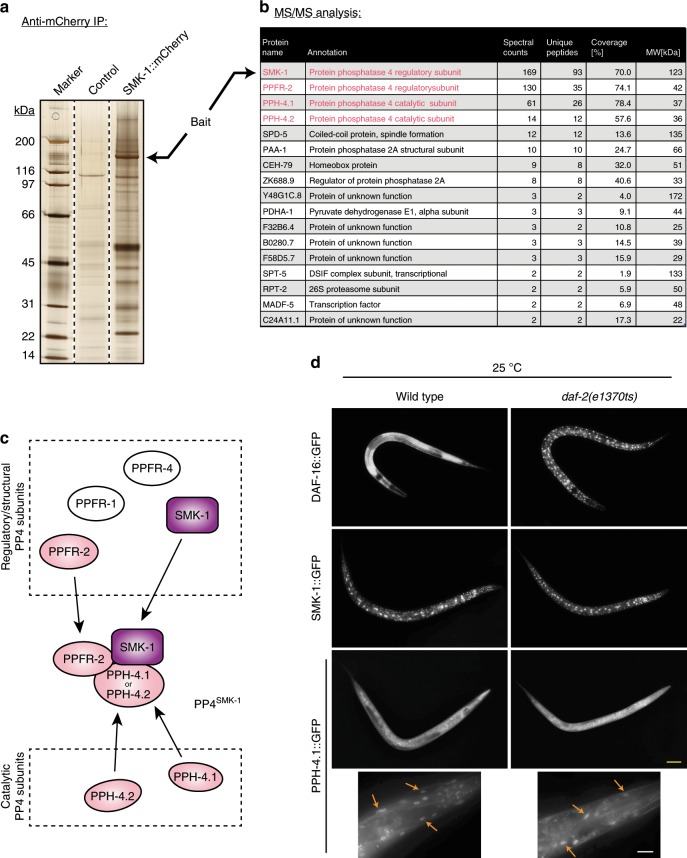
SMK-1 is part of a globally expressed nuclear Protein Phosphatase 4 complex. **a** SDS-PAGE and silver staining analysis of an anti-mCherry immunoprecipitation from whole-animal lysates of *C. elegans* expressing SMK-1::mCherry. Animals expressing no transgene were used as negative control. **b** List of the proteins identified in the purification from **a**, as determined by mass spectrometry (LC–MS/MS). Only proteins identified by at least two unique peptides are shown. See also Supplementary Table 2 for an independent repeat of this experiment. **c** Model illustrating the composition of the specific Protein Phosphatase 4 complex (PP4^SMK-1^) that co-purified with SMK-1, next to other implicated PP4 subunits not found in this complex. **d** Localization studies of DAF-16::GFP, SMK-1::GFP, and PPH-4.1::GFP in wild type and in *daf-2(e1370ts)* mutant animals. Worms were grown from the L1 stage at 15 °C, then shifted to 25 °C at the L2/L3 stage. After 16 h the GFP signal was recorded in L4 animals (yellow scale bar: 100 µm). The higher magnification images for PPH-4.1::GFP show head regions. Arrows are highlighting the partial nuclear accumulation of PPH-4.1 in both wild type and *daf-2(e1370ts)* animals (white scale bar: 20 µm).

To gain insight into the mechanism by which SMK-1 promotes DAF-16 functions, we sought to identify proteins bound to SMK-1. We grew *Caenorhabditis elegans* expressing SMK-1::mCherry to large quantity, lysed them, and conducted an immunoprecipitation (IP) using anti-mCherry antibody. The precipitate was then analyzed by silver staining (Fig. 1a) and tandem mass spectrometry (MS/MS, Fig. 1b). By this approach, we found 16 proteins that co-purified with SMK-1::mCherry (Fig. 1b, c, see Supplementary Table 2 for an independent repeat). The most abundant of these were subunits of Protein Phosphatase 4 (PP4); namely the catalytic subunits PPH-4.1 and PPH-4.2, and the regulatory subunit PPFR-2 (Fig. 1b, c). According to previous work in yeast and mammals, PP4 complexes exist in different defined subunit compositions. Each complex contains a catalytic subunit of PP4, of which the closest *C. elegans* orthologs are PPH-4.1 and PPH-4.2, and it contains a selection of four regulatory subunits, which help to target PP4 to its substrates^9–11^. Closest *C. elegans* orthologs to three of these regulatory subunits are PPFR-1, PPFR-2, and PPFR-4. Strikingly, closest ortholog to the fourth regulatory subunit is SMK-1. Together with our IP–MS/MS data, this suggests that SMK-1 is actually a regulatory subunit of a specific PP4 complex in *C. elegans*, hereafter referred to as PP4^SMK-1^. This complex has a unique subunit composition, containing either of the catalytic subunits and the regulatory subunits SMK-1 and PPFR-2, but not PPFR-1 or PPFR-4 (see also Fig. 1c). Next, we sought to confirm this result by independent IP–MS/MS experiments, using a different epitope tag on SMK-1 and a different affinity matrix (SMK-1::GFP and GFP-Trap, respectively). Given that SMK-1 is required for DAF-16 functions when IIS activity is low but not when it is high^8^, we further tested in this experiment whether the incorporation of SMK-1 into the PP4^SMK-1^ complex is IIS-dependent, by conducting the IPs from either *daf-2(e1370)* (a mutant of the insulin/IGF receptor, resulting in low IIS) or *daf-18(mg198)* (a mutant of PTEN, resulting in high IIS) animals. We confirmed the existence and composition of the PP4^SMK-1^ complex and found that the formation of this complex was not affected by IIS activity (Supplementary Table 3). As a final demonstration of how readily SMK-1 incorporates into PP4^SMK-1^ complexes in vivo, we expressed a minimal *C. elegans* PP4^SMK-1^ complex comprised of SMK-1::HA_3_, PPFR-2, and PPH-4.1::TAP in *Saccharomyces cerevisiae*, purified PPH-4.1-TAP by IgG pulldown, and then detected SMK-1::HA_3_ by western blotting. SMK-1::HA_3_ specifically co-purified with PPH-4.1::TAP (Supplementary Fig. 9d).

A surprising observation in these SMK-1 IPs was the prominent presence of the phosphatase subunit PAA-1 (Fig. 1b, c, Supplementary Tables 2, 3). PAA-1 is a well-established scaffold subunit of Protein Phosphatase 2A (PP2A) complexes^12^ that had not been found in PP4 complexes before. Our data suggests that, at least in *C. elegans*, PAA-1 may also be part of PP4 complexes, or alternatively that SMK-1 may be part not only of PP4^SMK-1^ but also a PP2A-related complex. Future studies will have to evaluate this.

Next, we were interested in the localization of the PP4^SMK-1^ complex, to get an indication of the tissues and cellular compartments, in which the complex regulates DAF-16 function under low IIS. We studied wild type and *daf-2(e1370)* animals expressing either SMK-1::GFP or PPH-4.1::GFP under their endogenous promoters. Animals expressing DAF-16::GFP served as additional control. We found that similar to DAF-16::GFP also SMK-1::GFP and PPH-4.1::GFP were globally expressed (Fig. 1d, Supplementary Fig. 1, see also^8,13^). Within cells, SMK-1::GFP localized strictly to the nucleus, while PPH-4.1::GFP could be seen in both the nucleus and cytoplasm (Fig. 1d, Supplementary Fig. 1). Expression pattern and subcellular localization of SMK-1::GFP and PPH-4.1::GFP were not affected by changes in IIS activity (Fig. 1d, Supplementary Fig. 1). This data showed that under low IIS the PP4^SMK-1^ complex has the opportunity to form in all tissues, and the intracellular localization patterns of its subunits suggest that the complex resides and exerts its functions in the nucleus.

### PP4^SMK-1^ is required for DAF-16 to promote longevity

**Fig. 2:**
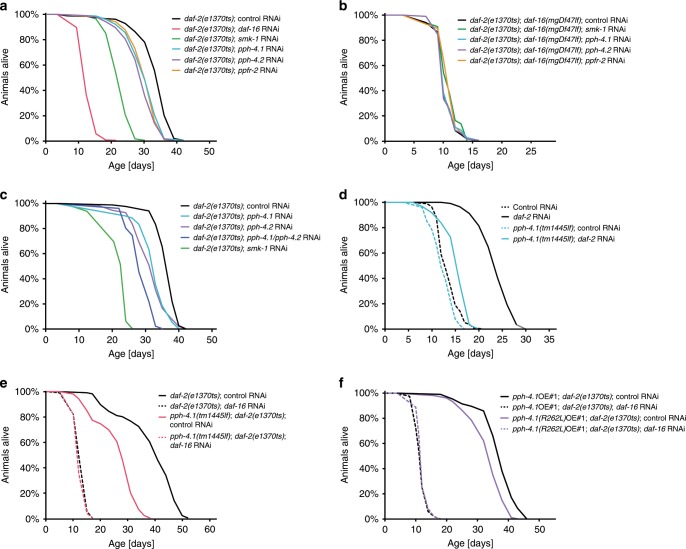
SMK-1 promotes the DAF-16-mediated longevity induced by low IIS as part of PP4^SMK-1^. Lifespan phenotypes caused by loss of *daf-16* or specific PP4 subunits in various genetic backgrounds. Animals of indicated genotypes were grown from the L1 stage on the indicated RNAi bacteria at 15 °C. At the L4 stage the animals were shifted to 25 °C to fully inactivate *daf-2(e1370ts)* and their lifespan was monitored. All strains used in **a**–**c** harbored the *eri-1(mg366ts)* mutation to yield better knockdown efficiencies^51^. **a** Individual knockdown of *daf-16* or any of the PP4^SMK-1^ subunits shortened the lifespan of *daf-2(e1370ts)* mutant animals. **b** All these phenotypes were abolished in *daf-16(mgDf47lf)* null mutant animals. **c** Combined knockdown of the catalytic PP4^SMK-1^ subunit paralogs *pph-4.1* and *pph-4.2* enhanced the lifespan-shortening effects of their individual knockdowns in *daf-2(e1370ts)* mutant animals. **d**, **e** The *pph-4.1(tm1445lf)* null mutation strongly impaired the longevity of low IIS animals in a *daf-16*-dependent manner, too. **f** Lifespan analyses of *daf-2(e1370ts)* mutant animals ectopically expressing either catalytically active PPH-4.1 or catalytic dead PPH-4.1(R262L). Expression of the catalytically dead PPH-4.1(R262L) specifically impaired the longevity of *daf-2(e1370ts)* mutant animals. For detailed statistics see Supplementary Table 4. Source data are provided as a Source Data file.

In light of this complex formation, we tested whether SMK-1 contributes to DAF-16’s functions as part of PP4^SMK-1^—in particular through the complex’s catalytic activity. First, we explored the role of PP4^SMK-1^ for lifespan regulation by DAF-16. We began by individually knocking down all PP4^SMK-1^ complex subunits by RNAi, either in *daf-2* mutant animals, which are long-lived due to constitutive DAF-16 activation, or in *daf-2; daf-16* mutant animals, where this DAF-16-driven effect is absent. Consistent with SMK-1 acting as part of PP4^SMK-1^, *smk-1* as well as *pph-4.1*, *pph-4.2* and *ppfr-2* were all required for full lifespan extension in *daf-2* mutant animals (Fig. 2a). Importantly, their influence on lifespan was completely dependent on the presence of *daf-16* (Fig. 2b). Next, we wanted to compare the ability of PP4^SMK-1^ versus other PP4 complexes to promote DAF-16 functions. For this we extended the previous experiment to include knockdowns of *ppfr-1* and *ppfr-4*—two PP4 subunits that we found to be absent from PP4^SMK-1^ (Fig. 1b, c). Consistent with PP4^SMK-1^ being the preferred PP4 configuration to regulate DAF-16 functions, loss of *ppfr-1* had no effect on lifespan (Supplementary Fig. 2a, b). And although loss of *ppfr-4* shortened the lifespan of *daf-2* animals, it did so in a manner independent of *daf-16* (Supplementary Fig. 2a, b).

In Fig. 2a, RNAi of *pph-4.1* or *pph-4.2* had a lesser effect on DAF-16-driven longevity than RNAi of *smk-1*. We attributed this to a possible redundancy of these two PP4 catalytic subunits. To test this, we conducted double-RNAi experiments targeting *pph-4.1* and *pph-4.2* together. Indeed, combined knockdown of both catalytic subunits led to additive suppression of longevity in *daf-2* animals, to an extent now more comparable to *smk-1* RNAi (Fig. 2c). Again, these lifespan phenotypes were dependent on *daf-16*, as illustrated by conducting the same knockdowns in wild type (Supplementary Fig. 2c) or *daf-2; daf-16* mutant animals (Supplementary Fig. 2d).

We then validated the phenotypes obtained from knockdown of the PP4^SMK-1^ catalytic subunits using a loss-of-function allele of *pph-4.1*, *pph-4.1(tm1445)*. This allele lacks exons 3 and 4 of *pph-4.1*, which contain the active site of the enzyme. Consistent with our RNAi data, this allele also specifically suppressed the longevity of *daf-2* mutant animals, while it had little effect in wild type and no effect in *daf-2; daf-16* mutant animals (Fig. 2d, e).

So far, our data was consistent with SMK-1 and PPH-4.1/4.2 each affecting lifespan as part of the PP4^SMK-1^ complex, acting through the same genetic pathway. If this were true, then combined depletion of *smk-1* and *pph-4.1/4.2* should lead to largely nonadditive phenotypes. We tested this by lifespan assays in *daf-2* mutant animals, confirming that this was indeed the case (Supplementary Fig. 2e).

Finally, we tested if it is truly the catalytic activity rather than structural properties of PP4^SMK-1^ that promote DAF-16 functions. We created transgenic lines overexpressing either wild type *pph-4.1* or an allele encoding an R262L mutant form of PPH-4.1, which has been shown to be catalytically inactive by studies in *C. elegans* and mammals^14,15^. Consistent with the importance of the catalytic activity of PP4^SMK-1^, overexpression of the catalytic dead form of PPH-4.1 led to partial suppression of longevity in *daf-2* mutant animals (Fig. 2f, Supplementary Fig. 2f), and this lifespan-shortening effect was lost upon knockdown of *daf-16* (Fig. 2f, Supplementary Fig. 2f).

We conclude that SMK-1 acts as part of a specific PP4 complex and uses its catalytic activity to promote DAF-16 functions, as shown here for DAF-16-driven longevity under low IIS.

### PP4^SMK-1^ promotes only some DAF-16-driven stress responses

**Fig. 3:**
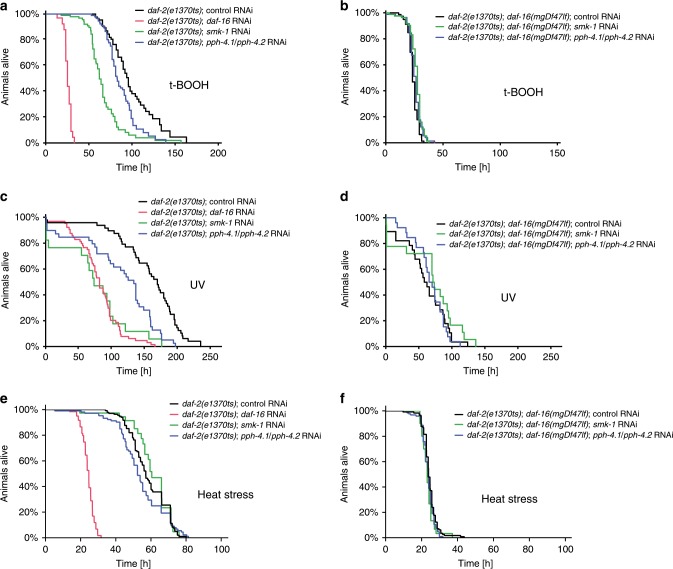
Under low IIS, PP4^SMK-1^ is required for DAF-16-mediated resistance to oxidative stress and UV. Stress survival phenotypes caused by loss of *daf-16* or PP4^SMK-1^ in various genetic backgrounds. Animals of indicated genotypes were grown at 15 °C from the L1 stage on the indicated RNAi bacteria. At the L4 stage the temperature was shifted to 25 °C to fully inactivate *daf-2(e1370ts)*. On day 2 adulthood, animals were then transferred to 6 mM tBOOH containing RNAi plates (oxidative stress), exposed to 1500 J/m^2^ UV light (UV stress), or shifted to 32 °C (heat stress). All strains used in in this figure harbored the *eri-1(mg366ts)* mutation to yield better knockdown efficiencies^51^. **a**, **c** Loss of PP4^SMK-1^, either by knockdown of SMK-1 or double knockdown of PPH-4.1/PPH-4.2, impaired the enhanced survival of *daf-2(e1370ts)* mutant animals that were exposed to oxidative stress (**a**) or UV stress (**c**). **b**, **d** These phenotypes were abolished in the absence of *daf-16*. **e**, **f** In contrast to oxidative stress and UV stress, loss of these PP4^SMK-1^ subunits did not impair the enhanced survival of *daf-2(e1370ts)* mutant animals exposed to heat stress. For detailed statistics see Supplementary Table 4. Source data are provided as a Source Data file.

Much of the DAF-16-driven longevity in *daf-2* mutant animals can be attributed to improved stress responses^6^. Consistently, a previous study had shown that SMK-1 was required for the *daf-16*-dependent improved resistance of *daf-2* animals to oxidative stress and UV irradiation^8^. Notably though, SMK-1 showed surprising specificity in mediating these phenotypes, as it did not affect the *daf-16*-dependent increased resistance to for example heat stress in these animals^8^. We now tested, if PP4^SMK-1^ confers stress resistance with similar specificity. For this we knocked down the catalytic subunits, *pph-4.1* and *pph-4.2*, by combined RNAi in either *daf-2* or *daf-2*; *daf-16* mutant animals and exposed them to either 1500 J/m^2^ UV irradiation or shifted them to 6 mM tert-butyl hydroperoxide (tBOOH; oxidative stress) or to 32 °C (heat stress) at day 2 of adulthood. Survival under these stresses was determined using a lifespan scoring machine^16^, to obtain data of particularly high resolution. We found that just like RNAi against *smk-1*, RNAi against *pph-4.1/pph-4.2* also led to reduced resistance to tBOOH and UV irradiation but not to heat (Fig. 3a, c, e). Furthermore, consistent with these being *daf-16*-dependent effects, these phenotypes were reduced in wild type animals when *daf-16* activity is reduced (Supplementary Fig. 3), and were entirely absent in *daf-16* null mutant animals (Fig. 3b, d, f).

These data illustrate that SMK-1’s roles in promoting stress resistance under low IIS also rely on the catalytic subunits of PP4^SMK-1^; and we could confirm the prior observation that SMK-1, and now PP4^SMK-1^, selectively help DAF-16 to confer a subset of its stress responses, e.g. resistance to oxidative stress and UV but not heat stress.

### PP4^SMK-1^ influence expression of many DAF-16 target genes

**Fig. 4:**
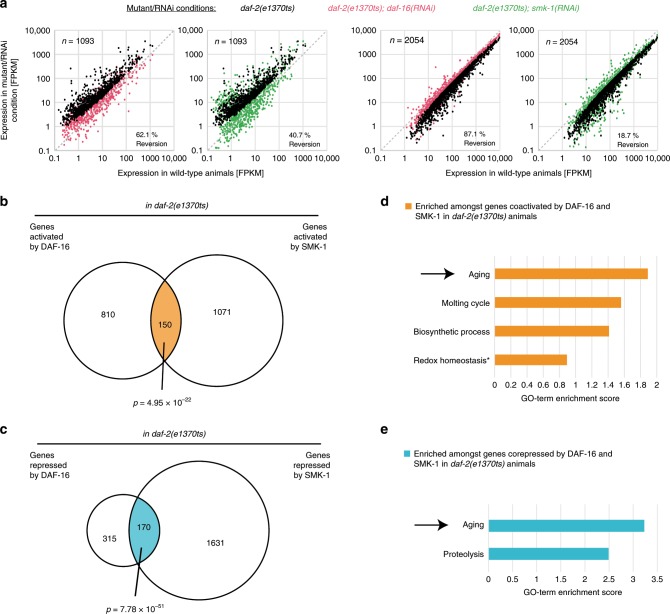
PP4^SMK-1^ is required for a substantial part of the DAF-16-mediated gene expression changes induced by low IIS. *daf-2(e1370ts)* mutant *C. elegans* were grown from the L1 stage on either control, *daf-16*, or *smk-1* RNAi bacteria. At the L4 stage, animals were shifted for 16 h to 25 °C, then harvested, and their transcriptomes determined by mRNA-seq. All strains used in in this figure harbored the *eri-1(mg366ts)* mutation to yield better knockdown efficiencies^51^. **a** Similar to *daf-16* loss, *smk-1* loss also suppresses many of the gene expression changes that occur under low IIS. The scatter plots on the left show the genes significantly upregulated and the scatter plots on the right show the genes significantly downregulated in *daf-2(e1370ts)* compared with wild type animals. **b**, **c** Venn diagrams illustrate the number of genes significantly regulated by DAF-16 or SMK-1 in *daf-2(e1370ts)* mutant animals as well as their overlap. **d**, **e** Functional enrichment analyses, conducted on the co-activated and co-repressed genes shown in **b** and **c**. Only significant enrichments are shown (DAVID scores ≥1.3, the symbol asterisk indicates that this term was still amongst the four most enriched GO-terms and relevant in the context of aging and stress responses, but it had a score below the significance threshold). Arrows highlight the GO-term “aging”, which turned out to be the most enriched in both analyses.

Given that PP4^SMK-1^ is required for many of DAF-16’s functions under low IIS, and because DAF-16 is a transcription factor, we wondered whether PP4^SMK-1^ acts by influencing the expression of DAF-16-regulated genes. We conducted mRNA-seq experiments using either wild type or *daf-2* mutant *C. elegans*. The animals were treated with either control, *daf-16*, or *smk-1* RNAi from the L1 stage and their mRNA sequenced at young adulthood. From the resulting dataset, we first determined the genes differentially expressed by the reduced IIS in *daf-2* mutant animals, identifying 1093 upregulated and 2054 downregulated genes (Fig. 4a). These gene expression changes were consistent with previous work^17^ (Supplementary Table 9). Next we asked how these differentially expressed genes are affected by RNAi against *daf-16* or *smk-1* in *daf-2* mutant animals. Most of the gene expression changes that occur in *daf-2* mutant animals are known to be mediated by DAF-16^6,17^. In agreement with this prior work, RNAi of *daf-16* suppressed 62.1% of the gene activation and 87.1% of the gene repression events in *daf-2* mutant animals (Fig. 4a, Supplementary Table 9). RNAi of *smk-1* also suppressed many of these gene expression changes, namely 40.7% and 18.7%, respectively (Fig. 4a). These findings were consistent with PP4^SMK-1^ indeed influencing the lifespan extending and largely DAF-16-induced gene expression changes under low IIS. Next we determined which actual genes were regulated by DAF-16 or SMK-1 under low IIS and which genes were common targets of both. As expected from the results in Fig. 4a, we found significant overlap amongst the genes that were activated or amongst the genes that were repressed by DAF-16 and SMK-1 (*p* = 4.95 × 10^−22^ and *p* = 7.78 × 10^−51^, respectively; Fig. 4b, c). And consistent with both DAF-16 and SMK-1 being required for the longevity under low IIS, we found these co-regulated genes to be enriched for genes related to aging (based on GO-term enrichment analyses, Fig. 4d, e).

Taken together, PP4^SMK-1^ is required for DAF-16 to properly regulate a subset of its target genes—many of which are aging related.

### PP4^SMK-1^ barely affects DAF-16 activation and DNA binding

Having established that PP4^SMK-1^ is important for many DAF-16 functions under low IIS, presumably by helping it to regulate aging-related target genes, we wondered how this function arises. Between the initial DAF-16-activating stimulus of low IIS activity and the successful regulation of target genes resides a long series of events. At which level of this cascade does PP4^SMK-1^ act? First, we studied the kinetics of nuclear entry and exit of DAF-16 upon inactivation and reactivation of the temperature-sensitive *daf-2(e1370)* allele, respectively (Supplementary Fig. 4a). Previous work only comparing translocation endpoints had found no impact of SMK-1 on the nuclear translocation of DAF-16^8^. Consistently, we observed no difference in the endpoints of our nuclear entry and nuclear exit time courses (Supplementary Figs. 4b, c, and  5). Only when we followed the kinetics of nuclear translocation we observed that knockdown of *smk-1* led to a mild delay, particularly in the entry of DAF-16 upon *daf-2* inactivation (Supplementary Figs. 4b, c and  5). However, we interpret this phenotype as very moderate and hence unlikely to explain the strong impact of PP4^SMK-1^ on DAF-16 functions.

Upon nuclear entry of DAF-16, it next needs to bind to its target promoters (Supplementary Fig. 4a). To see if this is influenced by PP4^SMK-1^ we determined the genome-wide binding of DAF-16 to its target promoters in the presence or absence of SMK-1. We used *daf-2* mutant animals expressing DAF-16::GFP, treated them with either control or *smk-1* RNAi, and then subjected them to chromatin immunoprecipitation (ChIP)-seq analysis using an anti-GFP antibody. Looking across 2824 DAF-16 binding sites described in previous work^18^, we found no significant change in the binding of DAF-16 to its target regions (*p* = 0.152; Supplementary Fig. 4d; see also Supplementary Fig. 4e for examples of individual genes contributing to Supplementary Fig. 4d).

In summary, absence of SMK-1 led to only a slight delay in DAF-16 nuclear entry and no significant change in the binding of DAF-16 to its target promoters. We therefore conclude that PP4^SMK-1^’s main impact on DAF-16 function should occur yet further downstream.

### PP4^SMK-1^ promotes transcription initiating events

**Fig. 5:**
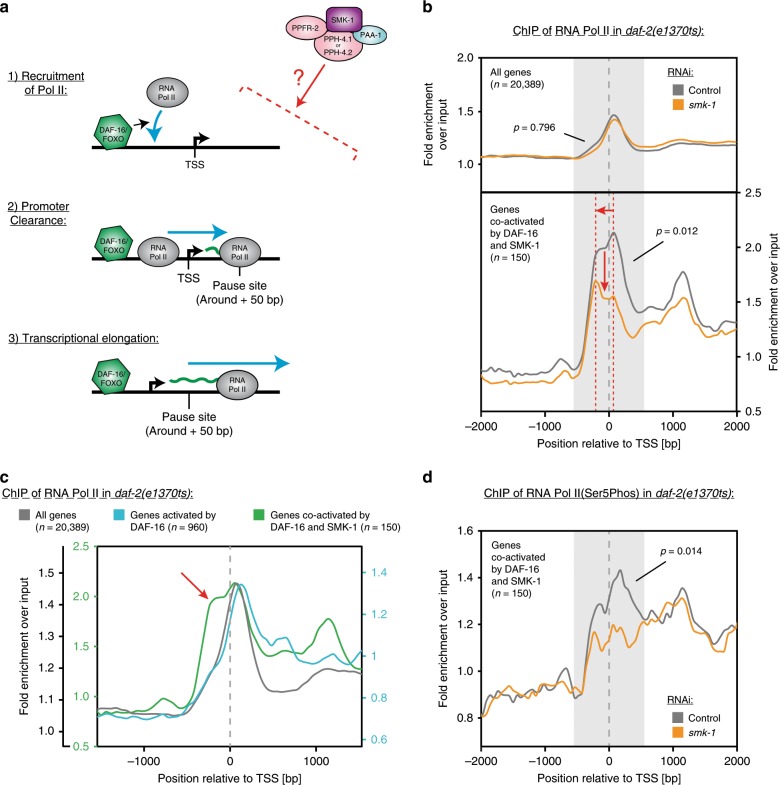
PP4^SMK-1^ is required for transcription initiating events at the genes co-activated by DAF-16 and SMK-1. **a** Schematic of the key steps that lead from DAF-16’s binding to promoter regions to the eventual transcription of the downstream genes. **b**–**d** ChIP-seq analysis of RNA Pol II under low IIS. *daf-2(e1370ts)* mutant animals were synchronized and grown from the L1 stage on either control or *smk-1* RNAi bacteria. At the L4 stage, animals were shifted to 25 °C. After an additional 16 h, animals were harvested and ChIP-seq analysis was conducted, using antibodies recognizing RNA Pol II, either in any modification state (**b**, **c**) or specifically RNA Pol II that was phosphorylated at Ser5 of its CTD (**d**). Average read densities across TSS regions of the indicated gene sets are shown. *p* values indicate the significance of read density differences in the grayed regions (−600 to +600 around the TSSs) between control and *smk-1* RNAi treated animals. Source data underlying Fig. 5b, d are provided as a Source Data file.

DAF-16 is predominantly a transcriptional activator^17^. Thus, upon binding its target promoters the next important downstream steps should be: (1) the recruitment of RNA Polymerase II (Pol II) to the promoters; (2) promoter clearance, which causes Pol II to leave the promoter region and move into the gene body where it often will pause around the +50 position (promoter-proximal pausing)^19,20^; (3) transcriptional elongation, during which Pol II moves beyond the pause site and transcribes the entire gene (Fig. 5a). To evaluate these steps we used *daf-2* mutant animals, subjected them to either control or *smk-1* RNAi, and then determined the genome-wide distribution of Pol II on chromatin by ChIP-seq using an antibody against the large subunit of Pol II, AMA-1. Strikingly, while Pol II recruitment was unaffected by *smk-1* RNAi when looking cumulatively at all promoter regions genome-wide (*p* = 0.796), we saw a substantial reduction in Pol II recruitment to promoters whose downstream genes would both be activated by DAF-16 and depend on SMK-1 for their activation (genes co-activated by DAF-16 and SMK-1 according to Fig. 4b) (*p* = 0.012; Fig. 5b, see left panels of Supplementary Fig. 6 for examples of genes that exhibit this phenotype).

Besides this apparent phenotype, we also noted an unexpected distribution of Pol II at these promoters. When we looked cumulatively at the regions around transcriptional start sites (TSSs) genome-wide, most Pol II had already undergone transcriptional initiation and localized to the pause site in the gene body (Fig. 5c). A similar distribution of Pol II could be found when looking cumulatively at all genes activated by DAF-16 (Fig. 5c). However, at the genes co-activated by DAF-16 and SMK-1, the distribution of Pol II was noticeably wider and a substantial amount of Pol II localized upstream of the TSS (Fig. 5c, see red arrow). We believe that this Pol II has been recruited to promoters but not fully undergone promoter clearance yet, which may indicate that the events of transcriptional initiation at DAF-16/SMK-1-co-activated genes occur slower and hence may be particularly rate limiting for their transcription^21^. Upon *smk-1* RNAi, presence of Pol II upstream of the TSS was even more pronounced (Fig. 5b), which would be consistent with SMK-1 promoting not only Pol II recruitment but also promoter clearance. However, we remain uncertain of this phenotype as it was difficult to recapitulate on the level of individual genes (see right panels of Supplementary Fig. 6 for example genes).

To further test our notion of transcription initiating events being impaired, we repeated our ChIP-seq experiments using an antibody that detects only initiating Pol II, which is phosphorylated at the Ser5 position within the repeats of its C-terminal domain (CTD)^22,23^. Indeed, we found significantly less initiating Pol II in the TSS regions of DAF-16/SMK-1-co-activated genes (Fig. 5d).

Finally, we investigated if PP4^SMK-1^ influences Pol II pausing, pause release or transcriptional elongation. A good indicator for defects in these processes are changes in the pausing index (the ratio of the average read densities of paused to elongating Pol II), as it can be derived from Pol II ChIP-seq data^24^. Calculating this index for DAF-16/SMK-1-co-activated genes under low IIS we found no impact of *smk-1* RNAi on the pausing index (Supplementary Table 6), suggesting that PP4^SMK-1^ had no direct role in Pol II pausing, pause release or transcriptional elongation.

We conclude that PP4^SMK-1^ is required for transcriptional initiation, predominantly by promoting efficient Pol II recruitment at a subset of DAF-16-activated target genes, and that these are genes whose expression may particularly depend on transcriptional initiation as a rate limiting step. We propose that this function is the major reason why PP4^SMK-1^ is required for the induction of many DAF-16 target genes under low IIS.

### The relevant substrate of PP4^SMK-1^ is SPT-5

We now had identified the steps in the cascade from DAF-16 activation to target gene expression which depend on PP4^SMK-1^. But PP4^SMK-1^ is a phosphatase, and we still did not know the relevant substrate whose dephosphorylation influences Pol II recruitment and behavior at a subset of DAF-16 target genes. First we took a candidate approach to test whether DAF-16 itself could be the target. However, neither our IP–MS/MS experiments (Fig. 1a, b, Supplementary Tables 2, 3, and ref. ^17^) nor published co-IPs^8^ provided evidence for a physical interaction between PP4^SMK-1^ and DAF-16. In addition, we investigated the *smk-1*-dependent phosphorylation status of DAF-16 by phos-tag gel-electrophoresis. We grew DAF-16::GFP-expressing *C. elegans* containing either *daf-2(e1370)* or *daf-18(mg198)* and exposed them to either control or *smk-1* by RNAi. As expected from high IIS activity leading to phosphorylation of DAF-16, we observed an upshift of DAF-16::GFP in *daf-18* versus *daf-2* mutant protein extracts when analyzed by a phos-tag gel and western blotting, but RNAi of *smk-1* had no effect on the migratory behavior of DAF-16::GFP in this assay (Supplementary Fig. 7). All this data argued that the relevant substrate of PP4^SMK-1^ is distinct from DAF-16.

**Fig. 6:**
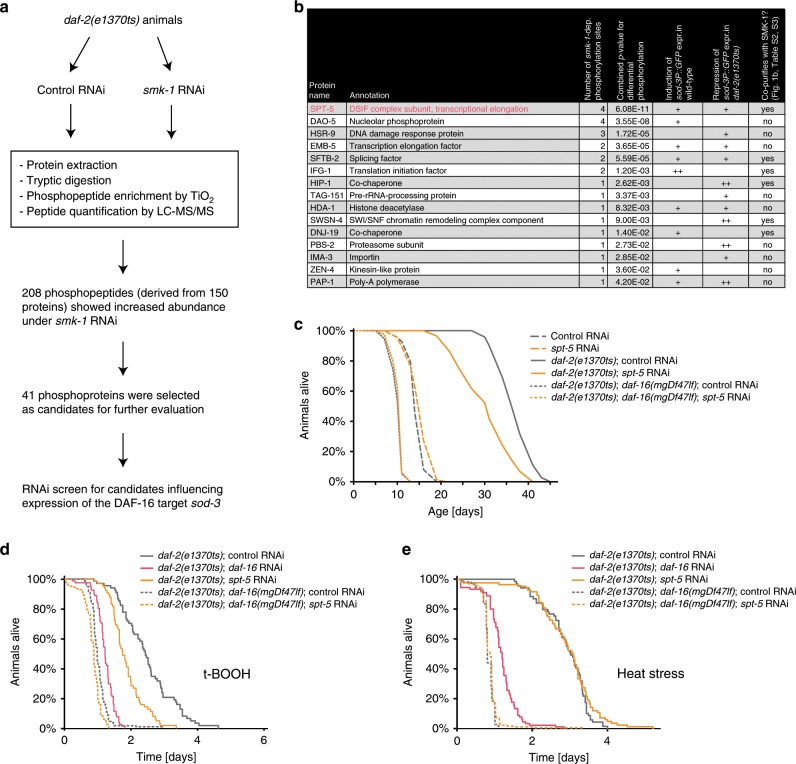
PP4^SMK-1^ promotes DAF-16’s functions under low IIS by dephosphorylating SPT-5. Identification of the relevant PP4^SMK-1^ substrate by unbiased phosphoproteomics and functional evaluation of the emerging candidates. **a** Workflow of the phosphoproteomics approach to identify PP4^SMK-1^ substrate candidates, followed by their functional evaluation through RNAi screening using a *sod-3P::GFP* reporter in either wild type or *daf-2(e1370ts)* backgrounds. **b** Results from the approach shown under **a**. The table is limited to proteins that were found significantly more phosphorylated upon *smk-1* RNAi and that additionally affected expression of *sod-3*, i.e., by *sod-3* induction in wild type or the repression of *sod-3* expression in *daf-2(e1370ts)* mutant animals. The table is ranked by the significance (*p* value) of the increased phosphorylation observed upon *smk-1* RNAi. SPT-5 is highlighted in red as being the top-ranked candidate from this prioritization approach. **c**–**e** Loss of SPT-5 phenocopied the stress resistance and lifespan phenotypes caused by loss of SMK-1. All strains used in in this figure harbored the *eri-1(mg366ts)* mutation to yield better knockdown efficiencies^51^. Wild type, *daf-2(e1370ts)*, or *daf-16(mgDf47lf)*; *daf-2(e1370ts)* mutant animals were grown from the L4 stage on either control or *spt-5* RNAi bacteria at 25°C. Animals remained either untreated (**c**), or were grown until day 2 of adulthood and then transferred to either 6 mM tBOOH containing RNAi plates (oxidative stress) (**d**) or shifted to 32 °C (heat stress) (**e**). Survival of the animals was monitored. For detailed statistics see Supplementary Table 4. Source data underlying Fig. 6c–e are provided as a Source Data file.

To identify the relevant substrate, we turned to an unbiased phosphoproteomics approach. We grew *daf-2* mutant animals to scale, treated them with either control or *smk-1* RNAi, lysed them, and determined their phosphoproteome by TiO_2_-based phosphopeptide enrichment and LC–MS/MS (see also Fig. 6a). 208 phosphopeptides whose abundance increased upon *smk-1* RNAi—and thus should be direct or indirect targets of PP4^SMK-1^—could be identified (Fig. 6a, Supplementary Table 7). The phosphopeptides originated from 150 proteins (Supplementary Table 7), of which we selected 41 for further evaluation based on their annotation and the significance of their *smk-1*-dependent differential phosphorylation. To determine the functional relevance of these candidate proteins, we used a transcriptional reporter for the canonical DAF-16 target gene *sod-3*. This reporter is induced by DAF-16 under low IIS—an induction that requires SMK-1^8^. Hence we knocked down each of the 41 candidates by RNAi in wild type or *daf-2* mutant animals carrying *Psod-3::GFP* and evaluated their GFP expression from day 1 to day 3 of adulthood. Knockdown of several candidates led to substantial changes in the expression of this reporter (Fig. 6b, Supplementary Fig. 8a). We additionally searched our SMK-1 IP–MS/MS data to see whether any of these candidates co-purified with PP4^SMK-1^, a behavior that often can be observed for phosphatase substrates^25^. Indeed, we found that several of our candidate substrates co-purified with SMK-1 (Figs. 1b and 6b, Supplementary Tables 2, 3). Together, these analyses highlighted one candidate in particular—the evolutionarily conserved protein SPT-5, which is predominantly known as a transcriptional elongation factor (Fig. 6b). Firstly, SPT-5 influences the expression of *sod-3* (Fig. 6b, Supplementary Fig. 8a). Secondly, SPT-5 harbors four phosphorylation sites that become more phosphorylated in the absence of PP4^SMK-1^ under low IIS (Fig. 6b, Supplementary Table 7). This differential phosphorylation of SPT-5 had the best *p* value of all *sod-3*-influencing candidates. Thirdly, SPT-5 physically associated with PP4^SMK-1^ according to our IP–MS/MS data (Figs. 1b and 6b, Supplementary Table 3)—an interaction that we were able to validate even between the human orthologs of SMK-1 and SPT-5 in HEK293T cells (Supplementary Fig. 8c). Finally, two additional transcription elongation factors from the same SPT family which closely synergize with SPT-5^26^ emerged from our study: (1) EMB-5^27^, which just like SPT-5 is a candidate substrate of PP4^SMK-1^ (Fig. 6b); and (2) SPT-4, which just like SPT-5 can be bound by PP4^SMK-1^ (Supplementary Table 3) and which together with SPT-5 forms the so-called DSIF (DRB sensitivity inducing factor) complex, involved in regulating Pol II’s promoter-proximal pausing and transcriptional elongation^28^. We then tested the consequences of losing any of these SPT family members for DAF-16-driven longevity and the increased resistance to oxidative and heat stress under low IIS. Already during the *sod-3* reporter assays we observed that RNAi of *spt-5* and *emb-5* from the L1 stage leads to developmental arrest. This is not surprising, as they should be essential for transcription throughout the genome^24^. Hence we conducted knockdowns of *spt-4* from the L1 or L4 stage, while *spt-5* and *emb-5* were knocked down from the L4 stage to overcome developmental phenotypes and hopefully leave behind sufficient residual amounts of these proteins to retain a basic level of genome-wide transcription. In these experiments, RNAi of *emb-5* or *spt-4* did not affect DAF-16-driven longevity under low IIS (Supplementary Fig. 8b; L1 feeding data for *spt-4* RNAi are not shown), leading us to exclude these SPT family members as relevant substrates. However strikingly, RNAi of *spt-5* led to phenotypes perfectly reminiscent of *smk-1* loss, namely impaired longevity and resistance to oxidative stress but not to heat stress in *daf-2* mutant animals, while *spt-5* RNAi had no effect in wild type or in *daf-2; daf-16* animals (Fig. 6c–e). Western blotting revealed that our knockdown eliminated ~92% of the SPT-5 protein (Supplementary Fig. 9a, b), which indicated that as little as ~8% of the physiological amount of SPT-5 is sufficient to assure a basic level of genome-wide transcription but is insufficient to help PP4^SMK-1^ promote the induction of DAF-16 target genes and therefore the mechanisms described in this study.

Overall, this data is consistent with SPT-5 being a key substrate by which PP4^SMK-1^ influences Pol II at many DAF-16 target genes and thereby fulfills its lifespan and stress resistance regulatory roles under low IIS.

### SPT-5 is a direct substrate of PP4^SMK-1^

Having identified SPT-5, we wanted to test if it is a direct or rather an indirect substrate of PP4^SMK-1^. To address this, we conducted in vitro phosphatase assays. First, we immunoprecipitated the PP4^SMK-1^ complex from *C. elegans* and transferred it to the appropriate assay buffer. Next, we incubated the complex with a variety of different synthetically made phosphopeptides which resembled the four PP4^SMK-1^-dependent phosphosites that we had identified by our phosphoproteomics. As a negative control, we incubated the complex with a phosphopeptide that is known to be targeted by the closely related phosphatase PP2A^29^. Remarkably, we found that PP4^SMK-1^ selectively dephosphorylated two of these phosphosites in SPT-5, S677, and S882, while it did not dephosphorylate the other phosphosites nor the negative control peptide (Supplementary Fig. 9c). To further confirm that this dephosphorylation was truly caused by PP4^SMK-1^ and not other *C. elegans* proteins or contaminants that might co-purify with the complex, we expressed a minimal PP4^SMK-1^ complex comprised of *C. elegans* PPH-4.1, PPFR-2, and SMK-1 recombinantly in *S. cerevisiae*. We purified this complex (see also Supplementary Fig. 9d), transferred it to assay buffer and conducted the same in vitro phosphatase assay as previously. Similar to the PP4^SMK-1^ complex purified from *C. elegans*, the recombinantly expressed minimal PP4^SMK-1^ complex also selectively dephosphorylated the sites S677 and S882 (Supplementary Fig. 9e). Finally, we tried to explore the purpose of the regulatory subunits, including SMK-1, in the PP4^SMK-1^ complex. Other studies have shown that regulatory subunits of PP4 can confer substrate binding and specificity^10,11,30,31^. Thus, we repeated our in vitro phosphatase assay one more time, now using only the catalytic subunit of PP4^SMK-1^, PPH-4.1, which we recombinantly expressed in *E. coli*. Notably, even though PPH-4.1 was sufficient to dephosphorylate the PP4^SMK-1^-targeted sites in vitro, the enzyme had now lost its selectivity and also dephosphorylated the SPT-5 phosphosite S904 or the PP2A target peptide with equal or even better efficiency (Supplementary Fig. 9f).

We conclude that PP4^SMK-1^ selectively dephosphorylates at least two phosphorylation sites in SPT-5 in vitro, which provides strong support for SPT-5 being a direct and specific substrate of PP4^SMK-1^ also in vivo.

## Discussion

The transcription factor DAF-16/FOXO is one of the most central and powerful aging regulators across metazoans, relaying many distress signals into compensatory, aging-preventive transcriptional responses. Nevertheless, it is not self-sufficient but depends on other proteins’ assistance. SMK-1, the first protein of this kind to be discovered^8^, is essential for DAF-16 to promote longevity and the resistance to many stresses under low IIS. This makes SMK-1 an important aging regulator in itself. Despite this prominence and the thorough genetic exploration of its functions^8^, it had remained unknown how SMK-1 is acting mechanistically. We could show that SMK-1 functions as part and through the catalytic activity of a specific Protein Phosphatase 4 complex, PP4^SMK-1^, and identified a direct substrate of this complex, SPT-5, whose dephosphorylation appears important for promoting DAF-16 functions under low IIS.

But how does PP4^SMK-1^-driven dephosphorylation of SPT-5 promote target gene expression by DAF-16? The literature describes SPT-5 predominantly as a transcriptional elongation factor and part of the conserved DSIF complex which it forms together with SPT-4^32^. This complex binds to Pol II during transcriptional initiation and remains associated with it throughout transcription^33,34^. DSIF is important for Pol II’s promoter-proximal pausing and transcriptional elongation^35^; but specifically SPT-5 was found to influence also other stages of transcription. Under certain circumstances it can influence Pol II’s binding to promoter regions and transcriptional initiation^36^. This has been proposed to originate from elongation-engaged SPT-5 inferring epigenetic changes in promoter regions that facilitate new rounds of preinitiation complex (PIC) formation and thereby Pol II recruitment^36^. Furthermore, SPT-5 facilitates promoter clearance by outcompeting PIC components like TFIIE from association with Pol II^36,37^. And last but not least, SPT-5 can influence transcriptional termination^34^. Several of these functions depend on the phosphorylation status SPT5^32,34^. Upon transcriptional initiation, Pol II and SPT-5 await elongation-promoting signals by the kinase P-TEFb, which usually phosphorylates both proteins at “proline-directed” sites (S/T-P) in their CTDs and thereby drives pause site release and transcriptional elongation^38–40^. SPT-5’s CTD remains phosphorylated throughout elongation and is dephosphorylated again near the transcriptional end site (TES), which contributes to transcriptional termination and release of Pol II and the DSIF complex from DNA^34^. In yeast, this dephosphorylation is conferred by Protein Phosphatase 1 (PP1)^34^.

All this prior work has already shown that SPT-5, even though it is mostly known as an elongation factor, can in principle influence most stages of transcription and that the phosphorylation state of SPT-5 is important for these roles. Such is consistent with our study showing that dephosphorylation of SPT-5 by PP4^SMK-1^ promotes transcription initiating events at DAF-16 target genes under low IIS. Only how this works exactly remains an open question. The actual phosphorylation status of the PP4^SMK-1^-targeted sites in SPT-5 during the different stages of transcription as well as off the DNA remains unknown. We can only say that they are overall hyperphosphorylated upon *smk-1* RNAi and that none of these sites, even though some of them are located within SPT-5’s CTD, are the canonical P-TEFb target sites mentioned above. Thus, we also do not know if PP4^SMK-1^ directly counteracts P-TEFb or whether it may dephosphorylate sites targeted by other kinases. The exact role of all phosphorylation sites on SPT-5 both inside and outside of its CTD, their turnover in the course of transcription, and the full portfolio of kinases and phosphatases that target them will be interesting topics of future investigation.

We would like to note that PP4^SMK-1^ predominantly promotes the step of Pol II recruitment to promoter regions, which is an event when SPT-5 has not yet bound to Pol II. Thus, in this context the effect of SPT-5 on Pol II should be indirect, which by analogy to previous observations^36^ may be caused by elongation-engaged SPT-5 conferring epigenetic changes in promoter regions that facilitate Pol II recruitment.

In a last effort to supplement our understanding of the PP4^SMK-1^–SPT-5–Pol II regulatory axis described in our study, we addressed three remaining questions. First, is it possible that PP4^SMK-1^ impacts transcription initiating events by regulating SPT-5’s recruitment to promoter/TSS regions? We addressed this by ChIP-seq analysis of DAF-16/SMK-1 co-activated genes in *daf-2* animals and found that knockdown of *smk-1* had no significant effect on SPT-5 recruitment (Supplementary Fig. 10a). This suggests that it is not absence of SPT-5 but its altered phosphorylation status that impairs transcription initiating events in the absence of PP4^SMK-1^. Second, we asked if, analogous to PP1 in yeast, PP4^SMK-1^ also influences transcriptional termination. Again through ChIP-seq in *daf-2* animals, we observed that *smk-1* RNAi causes a moderate shift of SPT-5 and Pol II distribution into the downstream direction, specifically at TESs of DAF-16/SMK-1-co-activated genes (Supplementary Fig. 10b, c). This result would be consistent with also transcriptional termination being moderately delayed at these genes. However, it remains to be tested whether and to what extend such termination defects would actually contribute to reduced expression of these genes. Third, we asked where PP4^SMK-1^ encounters SPT-5 and dephosphorylates it. We conducted ChIP-seq of SMK-1 using SMK-1::GFP-expressing *daf-2* mutant *C. elegans*. Association of SMK-1 with chromatin was extremely weak. However when looking cumulatively at all genes genome-wide, we observed a profile that would be consistent with SMK-1 binding to SPT-5 specifically during transcriptional elongation and dissociating just before the TES is reached (Supplementary Fig. 10d). This data argues that dephosphorylation of SPT-5 by PP4^SMK-1^ may occur during transcriptional elongation prior to termination. However, we cannot exclude that PP4^SMK-1^ acts also in the nucleoplasm, where it would dephosphorylate SPT-5 to prepare it for engagement in new rounds of transcription.

A remarkable aspect of our study is that PP4^SMK-1^ influences transcription through a highly conserved and common component of the transcription regulatory machinery, namely SPT-5. Nevertheless, it does not affect transcription genome-wide but only of a limited gene set. Such specificity actually has precedence. Studies in zebrafish and flies have similarly shown that mutations of SPT-5 affect only parts of the transcriptome^41,42^. But where would this specificity come from? We could see in Fig. 5c that promoter regions of DAF-16/SMK-1-co-activated genes may have unique properties, resulting in Pol II clearing these promoter regions more slowly. We propose that this makes them more sensitive to defects in transcription initiating events. Looking for sequence features that may distinguish these promoter regions, we found them enriched for TATA-boxes (TATA) (*p* = 2.45 × 10^−4^; Supplementary Table 8), while initiator elements (Inr) were not enriched (Supplementary Table 8). In contrast, DAF-16-activated promoters that do not depend on SMK-1 for their activation are depleted of both TATA and Inr (Supplementary Table 8). Notably, presence of TATA-boxes has been associated with genes for which transcriptional initiation is particularly rate limiting^43^. Considering all these observations, it would be consistent that loss of PP4^SMK-1^ and the resulting impairment of transcription initiating events selectively impair the expression of this subset of DAF-16 target genes. Regarding the purpose of such regulatory mechanism we can only speculate. It may be a feature of genes that need the ability to be rapidly and/or robustly induced in response to certain stimuli, i.e., a drop in IIS. Consistent with such notion, a recent study in humans showed particular involvement of SPT-5 in regulating the transcriptional initiation of rapidly-induced immune response genes^36^. Nevertheless, future studies will have to explore this in more detail.

It is also notable that PP4^SMK-1^, although it is required for most DAF-16 functions under low IIS, is irrelevant for some, e.g., for promoting resistance to heat stress (Fig. 3). Consistently, we observed that depletion of SPT-5 impaired longevity and oxidative stress resistance but not heat stress resistance under low IIS (Fig. 6c–e). This indicates that some DAF-16 target genes are regulated in a fundamentally different manner that does not involve the PP4^SMK-1^–SPT-5–Pol II axis, e.g., due to different promoter characteristics or DAF-16 fulfilling these functions in synergy with other transcription factors that impose different mechanisms of transcriptional control at these loci. Intriguingly, for heat shock response genes it is already known that DAF-16 regulates them combinatorially together with HSF-1^44^; and it has been shown that expression of HSF-1 target genes is not controlled on the level of Pol II recruitment or promoter clearance but rather by pause site release^45^.

**Fig. 7:**
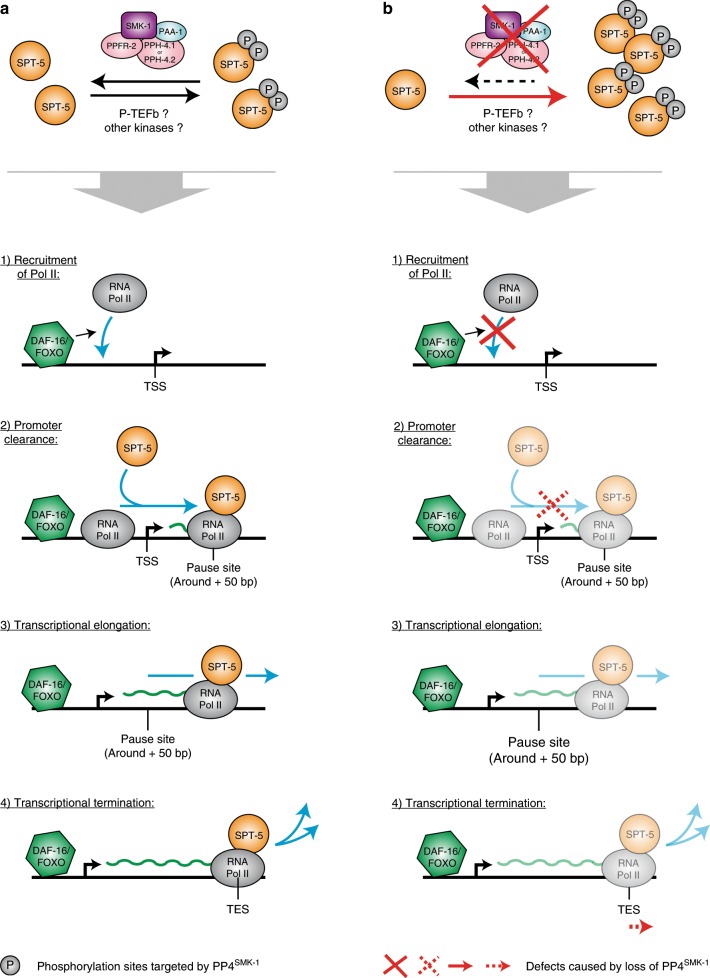
Model illustrating the roles of PP4^SMK-1^ and SPT-5 for the transcription of DAF-16/FOXO target genes. SPT-5 is known to get phosphorylated at various sites. Some of these sites, in particular proline-directed sites in the SPT-5 CTD, are targeted by the kinase P-TEFb, to promote transcriptional elongation. However, our study shows that other phosphorylation events in- and outside the SPT-5 CTD occur, too. We do not know the kinases which phosphorylate these other sites, be it P-TEFb or rather distinct kinases. However, we found that several of these other sites are dephosphorylated by the phosphatase PP4^SMK-1^. **a** Normally, the phosphorylation state of these PP4^SMK-1^-targeted sites is in balance. We unfortunately do not know during which stages of transcription they are phosphorylated nor when their dephosphorylation by PP4^SMK-1^ has to occur; but ultimately this balance allows for the efficient expression of DAF-16 target genes under low IIS. **b** In contrast, when PP4^SMK-1^ activity is missing, this balance shifts toward an overabundance of SPT-5 that is phosphorylated at these sites. Even though we do not understand the full mechanistic basis to it, this shift toward increased SPT-5 phosphorylation interferes with Pol II recruitment to promoter regions and may also mildly affect promoter clearance, leading to impaired transcription initiating events. Finally, a mild delay in transcriptional termination may occur, too. The red crosses and arrow indicate these phenotypes.

Taken together, our study provides substantial mechanistic insight and suggests a possible model for how SMK-1 promotes the expression of many DAF-16-activated genes under low IIS. Figure 7 illustrates this model and highlights the transcriptional events that seem affected when PP4^SMK-1^ activity is missing.

Intriguingly, SMK-1 may influence not only the expression of DAF-16-dependent genes but also the targets of other transcription factors, and it may promote longevity also in conditions besides low IIS. We obtained preliminary support for this notion when we compared the genes regulated by SMK-1 under low IIS to the genes regulated by two other transcription factors that also contribute to the longevity of *daf-2* mutant *C. elegans*—SKN-1^46^ and HLH-30^18^. In particular, genes regulated by HLH-30 were strongly dependent on SMK-1 for their regulation (Supplementary Table 9). A previous study investigating dietarily restricted *C. elegans* (*eat-2* mutants) showed that their longevity, which depends not on DAF-16 but rather PHA-4, was also dependent on SMK-1^47^. Finally, it was shown that SMK-1 is essential for the increased lifespan of mitochondrial electron transport chain mutants^8^. This points to an involvement of PP4^SMK-1^ also with other aging-regulatory signaling pathways and their downstream transcription factors, which will be interesting to explore further.

Finally we would like to highlight the strong conservation of all components mentioned in our study across metazoans. Two previous studies in *S. cerevisae* have observed a physical interaction between Psy2 (the *S. cerevisiae* ortholog of SMK-1) and Spt5 in tandem affinity purification-MS/MS experiments^9^, and we were able to confirm the existence of a PP4^SMEK^-SUPT5H complex under low IIS conditions in human cells (Supplementary Fig. 8c). This suggests that a PP4^SMEK^–SUPT5H–Pol II axis influencing the expression of FOXO target genes might exist in humans, too. It will be important to verify its conservation and whether it likewise promotes stress resistance and longevity.

## Methods

### *C. elegans* strains and alleles

For a complete list of strains used in this study, see Supplementary Table 1. Alleles obtained from other labs were 4 times outcrossed to the lab’s N2 background. All *C. elegans* strains were grown and maintained on *Escherichia coli* OP50 using standard methods^48^.

### RNAi by feeding

*C. elegans* were grown on the *E. coli* strain HT115 containing dsRNA-expressing plasmids targeting the genes of interest. RNAi clones either were obtained from published collections^49,50^ or were newly made. RNAi clones for *daf-2*, *pph-4.1, pph-4.2, ppfr-2, spt-4*, and *emb-5* were used from^49^ and RNAi clones for *daf-16*, *spt-5*, and *smk-1* were used from^50^. For screening purposes, all available RNAi clones from^49,50^ that targeted the genes of interest were used. RNAi clones targeting *ppfr-1* and *ppfr-4* were newly made by PCR-amplifying approximately 1 kb of cDNA or gDNA sequence located in the coding region of the gene of interest, cloning it into L4440 by AT-ligation, and then transforming the resulting construct into *E. coli* HT115. PCR primers CAACAACTTGGCGGTGCTATC (forward) and GTTCCGGATTGCAAGCAACG (reverse) were used on gDNA to make the *ppfr-1* RNAi clone; and CAAGCGCTTTACGATCCGAG (forward) and CATCCACGTCGATGATCATCC (reverse) were used on cDNA to make the *ppfr-4* RNAi clone. *E. coli* HT115 containing the empty plasmid L4440 served as negative control in the RNAi experiments. Combined knockdown of either the two PP4^SMK-1^ catalytic subunits *pph-4-1* and *pph-4.2* or of *pph-4.2* and *smk-1* was achieved by a 1:1 mixture of their RNAi bacteria.

Unless stated otherwise, RNAi feeding was initiated at the L1 stage. In most RNAi experiments, a RNAi-sensitized strain background (*eri-1(mg366)*) was used, to yield better knockdown efficiency^51^. Usage of this background is always stated in the respective figure legends.

### Generating transgenic *C. elegans*

To generate PPH-4.1::GFP-expressing *C. elegans*, the promoter of *pph-4.1* (the first 600 bp upstream of the START codon) was cloned from genomic DNA and the spliced *pph-4.1* coding region from cDNA of N2 animals. These sequences were then fused by PCR and cloned into the GFP-fusion expression vector pPD95_75 (Addgene), to yield the desired *pph-4.1P::pph-4.1::gfp* construct. Next, we coinjected this plasmid (30 ng/µl) with ScaI-digested pUC19 (40 ng/µl) and EcoRI-digested pRF4 (30 ng/µl) to yield complex arrays marked by *rol-6(su1006)*. Injections were performed using an inverted DIC microscope (Zeiss) in combination with an InjectMan 4 and FemtoJet 4i (Eppendorf). Rollers were selected from the F1 progeny and lines were established.

*C. elegans* expressing catalytically dead PPH-4.1(R262L) were generated in the same way—only that we mutated the *pph-4.1P::pph-4.1::gfp* construct by site-directed mutagenesis, replacing AGA by CTT.

### Microscopy and nuclear translocation assays

For imaging, L4 stage worms were paralyzed by 2,3-butanedione monoxime (BDM), mounted on 2% agarose pads, and imaged using a Zeiss Axio Observer Z1 microscope at 20x or 40x magnifications.

To investigate the effect of SMK-1 on nuclear entry of DAF-16, *daf-2(e1370)* mutant animals expressing DAF-16::GFP were grown from the L1 stage at 15 °C on either control or *smk-1* RNAi. On day 2 of adulthood, animals were shifted to 25 °C, upon which moment nuclear entry was scored in the intestine of the animals. To investigate the effect of SMK-1 on nuclear exit of DAF-16, animals were grown in the same way to day 1 adulthood, shifted to 25 °C for 24 h to reach full nuclear entry of DAF-16, and then shifted back to 15 °C, upon which moment nuclear exit was scored in the intestine of the animals. Scoring was done blinded, using a Zeiss Axio Zoom V16 microscope.

### Testing the knockdown efficiency of spt-5 RNAi

*daf-2(e1370)* animals expressing SPT-5::GFP were synchronized by bleaching, grown on HT115 bacteria without dsRNA-expressing plasmids at 15 °C until the L4 stage, washed in antibiotics to remove the HT115, and transferred to plates seeded with either control or *spt-5* RNAi bacteria. 5-fluoro-2′-deoxyuridine (FUDR) was added to a final concentration of 50 µM to prevent progeny production and the plates were shifted to 25 °C. On day 2 of adulthood, 50 worms per condition were harvested and analyzed by SDS-PAGE and western blotting. For detection of SPT-5::GFP we used monoclonal mouse anti-GFP antibody (Roche) and for detection of actin we used monoclonal (C4) mouse anti-actin antibody—both diluted 1:1000 in TBST containing 5% milk powder. For quantification, band intensities were determined using the Fiji image processing package (ImageJ) and data were normalized to actin. This experiment was repeated 3 times. Unpaired *t* test (two-tailed) was used for comparisons and statistical analysis.

### Lifespan assays

*C. elegans* were synchronized by bleaching and grown, depending on the experiment, on either OP50 or RNAi bacteria at 15 °C until the late L4 stage. In cases where the RNAi bacteria would lead to developmental arrest, animals were initially grown on HT115 bacteria without dsRNA-expressing plasmids at 15 °C and only at the L4 stage washed in antibiotics to remove the HT115 and transferred to RNAi bacteria. In all cases 5-fluoro-2′-deoxyuridine (FUDR) was added to a final concentration of 50 µM at the late L4 stage to prevent progeny production and the plates were shifted to 25 °C. Survival of animals was determined every 2–3 days by scoring movement upon prodding with a platinum wire^52^.

### Stress survival assays

*C. elegans* were synchronized by bleaching and grown, depending on the experiment, on either OP50 or RNAi bacteria at 15 °C until the late L4 stage. In cases where the RNAi bacteria would lead to developmental arrest, animals were initially grown on HT115 bacteria without dsRNA-expressing plasmids at 15 °C and only at the L4 stage washed in antibiotics to remove the HT115 and transferred to RNAi bacteria. In all cases FUDR (50 µM final concentration) was added at the late L4 stage to prevent progeny production and the plates were shifted to 25 °C. At day 2 of adulthood, animals were transferred to plates containing 6 mM tBOOH (for oxidative stress assays), the temperature was shifted to 32 °C (for heat stress assays), or animals were exposed to 1500 J/m^2^ UV light (for UV stress assays). Their survival was recorded and analyzed by a fully automated lifespan machine^16^.

### Immunoprecipitation from *C. elegans*

Growth of *C. elegans* to large scale, harvesting, lysis, IP of the bait proteins, and eventual analysis of the precipitated material by tandem mass spectrometry were conducted as previously described^17^. At first, the animals were grown asynchronously at 15 °C to scale, then shifted for 16 h to 25 °C and harvested. Animals were transferred to lysis buffer (50 mM HEPES at pH 7.4, 1 mM EGTA, 1 mM MgCl2, 150 mM KCl, 10% (v/v) glycerol, Complete without EDTA (Roche), 1 mM phenylmethyl sulphonyl fluoride, and phosphatase inhibitors (Sigma)) and lysed by grinding under liquid nitrogen. NP-40 was added to 0.05% (v/v), and the resulting lysate was cleared at 20,000 g. mCherry-tagged bait proteins were immunoprecipitated using a rabbit-anti-mCherry antibody that we generated in the lab and coupled to Protein A resin (Biorad). GFP-tagged bait proteins were immunoprecipitated using GFP-trap resin (Chromotek). Immunoprecipitated proteins were eluted using 100 mM glycine at pH 2.6. As negative controls for background subtraction, the same purifications and later mass spectrometry analyses were conducted using wild type (untagged) *C. elegans*.

### Mass spectrometry to identify co-purifying proteins

Immunoprecipitated proteins were digested overnight at pH 8.3 using sequencing-grade trypsin. Resulting peptides were separated by microcapillary reverse-phase chromatography and identified by online tandem mass spectrometry using an LTQ Orbitrap XL hybrid linear ion trap-orbitrap high-resolution mass spectrometer (Thermo). Tandem mass spectra produced by collision induced dissociation (CID) in the ion trap were searched against the Wormpep database (version 246) using ProLuCID^53^ and DTASelect^54^. Enzyme specificity was set to trypsin, allowing cleavage N-terminally to proline. Further modifications were cysteine carbamidomethylation (fixed), as well as methionine oxidation and phosphorylation of the amino acids S,T, or Y (all variable). False discovery rates (FDRs) were determined by searching the mass spectra against the reversed sequences of the Wormpep database. Proteins had to be identified by at least two independent peptides, resulting in an FDR below 1%. Finally, samples were compared using Contrast^54^. Any proteins that were identified also in the control purifications (from untagged *C. elegans* lysates) were likely contaminants and thus eliminated from Fig. 1b and Supplementary Tables 2, 3.

### Co-immunoprecipitation from HEK293T cells

The HEK293T cells (ATCC, tested to be void of mycoplasma contamination) were grown in advanced Dulbecco’s modified Eagle’s medium/F12 supplemented with 10% fetal bovine serum and then transfected in 9 cm dishes with 15 μg of plasmid DNA for GFP-SMEK1 (Addgene), using the Profection mammalian transfection system (Promega). 12 h post transfection, medium was replaced with fresh antibiotic-free media and LY294002 was added at a final concentration of 20 μM. After 48 h of LY294002 treatment, cells were harvested by scraping and frozen in liquid nitrogen. For the co-IPs, these cell pellets were thawed into lysis buffer (50 mM HEPES at pH 7.4, 1 mM EGTA, 1 mM MgCl2, 150 mM KCl, 10% (v/v) glycerol, Complete (Roche), 1 mM phenylmethyl sulphonyl fluoride, phosphatase inhibitors (Roche), and 0.5% (v/v) NP-40) and lysed by shearing through a G30 syringe. Lysates were cleared at 20,000 g. GFP-tagged proteins were immunoprecipitated using GFP-Trap resin (Chromotek) and eluted by boiling in 2× sample buffer. Input (IN) and eluate (IP) samples were analyzed by SDS-PAGE and western blotting.

### Production of recombinant proteins in *E. coli*

The cDNA of *pph-4.1* was cloned into the GST-fusion expression vector pGEX-4T1 and the resulting plasmid transformed into BL21(DE3)-RIPL (Agilent). The resulting bacterial strain was grown to an OD_600_ of 0.6 at 37 °C and 230 rpm and then induced with IPTG for 18 h at 18 °C and 230 rpm. Next the bacteria were harvested and frozen in liquid nitrogen. For the purification of PPH-4.1, the bacteria were thawed into TBS buffer containing 1 mM EDTA, 1 mM DTT, and Complete without EDTA (Roche), and incubated with lysozyme for 30 min at 4 °C. Resulting lysate was sonicated, Triton X-100, RNase A (Roche), and DNase I (Invitrogen) were added to concentrations of 1% (v/v), 10 µg ml^−1^, and 5 µg ml^−1^, respectively, and the lysate then incubated for another 30 min at 4 °C. Glutathione-Sepharose 4B resin (GE Healthcare) was added to the lysate and incubated rotating for 2 h at 4 °C. Finally, the resin was washed in TBS, resuspended in TBS, and then used for in vitro phosphatase assays.

### Production of recombinant proteins in *S. cerevisiae*

The cDNA of *pph-4.1* was fused to a TAP tag and then used to replace the cdt1 gene in the plasmid pJF2^55^, creating a Gal1 promoter-driven expression construct. The cDNA of *ppfr-2* was used to replace mcm7 and the cDNA of *smk-1* was fused to a HA_3_ tag and then used to replace mcm6 in the plasmid pJF4^55^, creating a Gal1-10 promoter-driven expression construct. Both expression constructs were integrated into the genome of the *S. cerevisiae* strain CB3245 (MATa, pep4∆::HPH). Resulting cells were grown at 30 °C and 200 rpm in YEP medium containing 2% raffinose to an OD_600_ of 1.0, then 2% galactose was added to induce the overexpression and the cells were grown for an additional 4 h at 30 °C and 200 rpm. Cells were harvested by centrifugation, washed with IPP150 buffer (50 mM Tris-HCl (pH 8.0), 150 mM NaCl, 10% glycerol, 0.1% NP-40, and 1 mM dithiothreitol (DTT)) and ground in liquid nitrogen using a SPEX 6870 freezer mill. Ground cells were dissolved in 1 ml IPP150 buffer containing Complete without EDTA (Roche), 10 mM MgCl_2_ and 12.5 U Benzonase (Sigma), and incubated for 1 h at 4 °C. The lysate was cleared by centrifugation for 20 min at 16.000 *g*, and the purification of the TAP-tagged protein was performed as described before^56^ with slight modifications. As beads for the purification we used IgG Sepharose 6 Fast Flow (GE Healthcare). Glutathione-Sepharose 4B beads (GE Healthcare) were used for the mock purification. Beads were divided into two tubes and washed with GF150 buffer (20 mM HEPES (pH 7.5), 150 mM NaCl, 10% glycerol, 0.1% NP-40, and 1 mM DTT). Finally, the PPH-4.1::TAP was eluted with TEV protease and used for in vitro phosphatase assays.

For detection of PPH-4.1::TAP by western blotting we used anti-TAP antibody (antibodies online.com, Cat#ABIN398491) diluted 1:1000 in TBST containing 2% (w/v) milk powder (Sigma). For detection of SMK-1::HA_3_, we used anti-HA antibody (Abcam, cat # ab9110) diluted 1:3000 in TBST containing 5% (w/v) milk powder.

### PP4^SMK-1^ purification from *C. elegans* for in vitro assays

*daf-2(e1370)* animals expressing SMK-1::GFP were grown asynchronously to scale at 15 °C, then shifted for 16 h to 25 °C and harvested. Animals were transferred to lysis buffer (50 mM HEPES at pH 7.4, 1 mM EGTA, 1 mM MgCl_2_, 150 mM KCl, 10% (v/v) glycerol, Complete without EDTA (Roche)), and lysed by grinding under liquid nitrogen. NP-40 was added to 0.05% (v/v), and the resulting lysate was cleared at 20,000 g. The SMK-1::GFP was immunoprecipitated using GFP-trap resin (Chromotek), the resin washed, and then resuspended in IP-EL buffer (50 mM HEPES at pH 7.4, 1 mM EGTA, 1 mM MgCl_2_, 150 mM KCl, and 10% (v/v) glycerol), to be used in the in vitro phosphatase assays.

### In vitro phosphatase assay

We designed phosphopeptides for each PP4^SMK-1^-dependent phosphosite on SPT-5 (S671: PMVSRMAS(p)PNPMAS, S677: ASPNPMAS(p)PRHSSGG, S882: TPAYGS(p)ADGAR, S904: TPAYGS(p)TEGGR). In addition, we included a known PP2A substrate peptide (“PP2A target”), derived from the retinoblastoma protein RB^29^ (PP2A target: INGSPRT(p)PRRGQNR), as a negative control. These peptides were synthesized (95% purity, TAG Copenhagen A/S) and, prior to assays, freshly diluted to 1 mM final concentration in pNPP Ser/Thr Assay Buffer (50 mM Tris-HCl (pH 7.0), 100 µM CaCl_2_). Purified material (either 10 µl beads or 10 µl eluted protein) was mixed with 40 µl of 1 mM Phosphopeptide solution. Samples were incubated at RT for 15 min on a shaking incubator at 800 rpm. All these assays were conducted in triplicate. For the eventual detection of the released phosphate, samples were transferred to 96-well half area assay plates (Corning) and diluted fivefold in pNPP Ser/Thr Assay Buffer to reach detectable range. We used a Malachite Green Phosphate Assay Kit (Sigma, cat # MAK307) and performed the assay according to the manufacturer’s instructions. One-way ANOVA and post hoc Dunnett’s test were used for statistical calculations and multiple comparisons of each sample with the control substrate (“PP2A target”).

### Phos-tag SDS-PAGE analysis

Worms were grown on either control or *smk-1* RNAi bacteria until day 2 of adulthood and then harvested into a lysis buffer (20 mM MOPS (pH 7.2), 15 mM MgCl_2_, 100 mM NaCl, 1% (v/v) Triton X-100, 1 mM DTT, Complete without EDTA (Roche), 1 mM phenylmethyl sulphonyl fluoride, and PhosSTOP (Roche)). Samples were lysed using a FastPrep and silicon carbide beads with 1.0 mm diameter (BioSpec). Per condition, material equivalent to 50 worms was loaded onto SuperSep Phos-tag (50 µM) 10% precast gels (Wako) and then analyzed by SDS-PAGE and western blotting. Monoclonal mouse anti-GFP antibody (Roche) diluted 1:1000 in TBST containing 5% milk powder was used to detect DAF-16::GFP in the different samples.

### Sample preparation for phosphoproteomic analyses

*daf-2(e1370)* mutant worms were synchronized by bleaching, seeded as L1 larvae onto plates containing either control or *smk-1* RNAi bacteria, and grown at 15 °C until the L4 stage. Plates were then shifted to 25 °C and FUDR (50 µM final concentration) was added to prevent progeny production. On day 2 of adulthood, animals were harvested, washed, and frozen in liquid nitrogen. Animals were lysed by grinding under liquid nitrogen, using a cryomill (Retsch). Then 300 mg of worm powder were thawed and 200 µl of the resulting lysate transferred to a new tube, where it was diluted with 300 µl of lysis buffer (8 M Urea, 1% SDS, 50 mM Tris pH 8.5, protease inhibitors (Roche), and phosphatase inhibitors (Roche)). Samples were then sonicated on ice to shear the DNA and cleared by centrifugation for 10 min at 4 °C and 20,000 *g*. Supernatants were transferred to new tubes and their protein concentration measured using BCA assay (Pierce). Six hundred micrograms of proteins were reduced by adding DTT to a final concentration of 5 mM and incubating for 1 h at 25 °C. Samples were then alkylated by adding iodoacetamide to a final concentration of 15 mM and incubating for 30 min in the dark at room temperature. Excess iodoacetamide was quenched by adding DTT to an additional 10 mM of final concentration. Proteins were isolated by methanol/chloroform precipitation, redissolved in 100 µl of 50 mM Tris pH 8.5 containing 8 M urea, and then diluted with an equal volume of 50 mM Tris pH 8.5. Proteins were digested by adding 10 µg of LysC (Promega) and incubating for 6 h at 24 °C and 300 rpm. Next, three volumes of 50 mM Tris pH 8.5 were added to reach a final urea concentration of 1 M. 10 µg of LC grade trypsin (Promega) was added and samples were incubated overnight at room temperature. After digestion, TFA was added to 0.5% (v/v) and samples were centrifuged for 10 min at 4 °C and 20,000 g. The pellet was discarded and the supernatant desalted using C18 SepPak cartridges (Waters). Phosphopeptides were enriched using a TiO_2_ Phosphopeptide Enrichment and Clean-up Kit (Pierce) according to the manufacturer’s recommendations.

### Mass spectrometry for phosphoproteomic analyses

Peptides were separated using a 50 cm EASY-Spray™ LC column (Thermo Scientific) and the EASY-LC 1000 chromatography system (Thermo Scientific). Separation was achieved by a 120 min linear gradient from 2 to 26% acetonitrile in 0.1% formic acid at a flow rate of 300 nL/min. Eluting peptides were ionized by electron spray and analyzed by an Orbitrap Fusion mass spectrometer (Thermo Fisher Scientific). The survey MS spectrum was acquired at a resolution of *R* = 120,000 in the range of m/z 200–2000. MS/MS data for the 20 most intense precursors were obtained using higher-energy collisional dissociation for ions with a charge *z* > 1 at a resolution of *R* = 15,000.

### Data analysis for phosphoproteomic analyses

RAW data from the mass spectrometer were analyzed using MaxQuant 1.5.3.30^57^^.^ A FDR of 0.01 for proteins and peptides and a minimum peptide length of six amino acids were required. The Andromeda search engine^58^ was used to search the MS/MS spectra against the Uniprot *C. elegans* database (WBcel235) combined with 262 common contaminants and concatenated with the reversed versions of all sequences. Enzyme specificity was set to trypsin, allowing cleavage N-terminally to proline. Further modifications were cysteine carbamidomethylation (fixed), as well as protein N-terminal acetylation, asparagine and glutamine deamidation, methionine oxidation, and phosphorylation of the amino acids S,T, or Y (all variable). Further evaluation of the data provided by MaxQuant was performed using R^59^. Proteins’ relative intensities were normalized to the total protein signal. Differences in relative protein abundances between treated and control samples were assessed by moderated *t* test using the R-package limma^60^. Benjamini–Hochberg correction for multiple comparisons was used.

### Transcriptional reporter assays

The *sod-3p::GFP*-expressing *C. elegans* strains CF1553 or AS23 were synchronized by bleaching and seeded as L1 larvae onto plates containing RNAi bacteria, grown at 15 °C until the L4 stage, and then shifted to 25 °C. For each gene of interest, all RNAi clones available in the two major genome-wide collections^49,50^ were tested and we then used the ones with the strongest phenotypes. Any RNAi clones that would lead to developmental arrests were retested by initially growing the animals on HT115 bacteria without dsRNA-expressing plasmids at 15 °C and only at the L4 stage washing them in antibiotics to remove the HT115 and transferring them to the respective RNAi bacteria and to 25 °C. In all cases, production of progeny was prevented by addition of FUDR (50 µM final concentration) at the late L4 stage. GFP expression was evaluated/scored by visual inspection from day 1 to day 3 of adulthood, using a Zeiss Axio Zoom V16 microscope.

### mRNA isolation and sequencing

Worms were synchronized by bleaching and about 150 of them grown from the L1 stage at 15 °C on control, *smk-1*, or *daf-16* RNAi bacteria. At the L4 stage, the plates were shifted to 25 °C and FUDR (50 µM final concentration) was added to prevent production of progeny. 16 h later, 100 animals were collected, washed, and immediately frozen in liquid nitrogen. Total RNA was extracted using Trizol (Sigma). RNA quality was assessed by determining its RNA integrity number (RIN). We required all samples to have a RIN above 9. mRNA-seq libraries were constructed using a TruSeq RNA SamplePrep V2 kit (Illumina) according to the manufacturer’s instructions.

### Chromatin immunoprecipitation

Approximately 800,000 *C. elegans* were used per condition in each experiment. Worms were either grown asynchronously at 15 °C on large plates seeded with OP50 bacteria or they were synchronized by bleaching, L1 larvae seeded onto plates containing either control or *smk-1* RNAi bacteria, and the animals grown at 15 °C until the late L4 stage. For the RNAi experiments, FUDR (50 µM final concentration) was added to prevent production of progeny. The animals were then shifted to 25 °C, grown for another 16–18 h, harvested, and subjected to chromatin immunoprecipitation (ChIP)^17^. Briefly, ground frozen worm powder was crosslinked using 1% formaldehyde in PBS and sonicated. IP was performed using Protein A Dynabeads (Invitrogen) and either rabbit polyclonal anti-GFP antibody (Takara, Cat# 632592), mouse monoclonal anti-RNA Polymerase II antibody [8WG16] (BioLegend, Cat# 920102), or mouse monoclonal anti-RNA polymerase II antibody that specifically detects CTD repeats phosphorylated at Ser5 [4H8] (Abcam, Cat# ab5408). Protein–DNA complexes were then eluted from protein A beads, treated with RNase A and proteinase K, and the resulting DNA purified. Sequencing libraries of inputs and immunoprecipitated samples were constructed using a protocol optimized for small DNA quantities^61^.

### High-throughput sequencing

For both multiplexed mRNA-seq and ChIP-seq libraries, single-end sequencing was conducted for 50 cycles on Illumina HiSeq 2000 sequencers according to the manufacturer’s instructions. Image analysis, base calling, and quality scoring were carried out in real time with the standard Illumina analysis pipeline using a phiX control.

### Processing of mRNA-seq reads and downstream analysis

Reads were mapped to the *C. elegans* genome using TopHat (v2.0.8b)^62^ and known gene model annotations (WS220), as well as the following parameters: --library-type fr-unstranded --b2-very-sensitive --min-coverage-intron 10 --min-segment-intron 10 --microexon-search --no-novel-juncs. Transcript abundance (FPKM, fragments per kilo base of transcript per million fragments) and differential expression was calculated using Cuffdiff (v2.1.1) from the Cufflinks software package^63^ and the following parameters: -u --FDR 0.05 --upper-quartile-norm --compatible-hits-norm --library-type fr-unstranded. All conditions were analyzed at least by using two biological replicates. Replicates were mapped individually and then combined by Cuffdiff. All analyses were limited to protein-coding genes. Statistically significant differentially expressed genes (DEGs, in other words “up- and downregulated” genes), were identified by Cuffdiff, using a 5% FDR. Differential gene expression values were calculated as the ratio of FPKM values. To test for significant overlap between gene lists, we used the hypergeometric test (R function phyper^59^).

### Processing of ChIP-seq reads and metagene analysis

For basic quality metrics of our ChIP-seq samples see Supplementary Table 5. We aligned the reads to the *C. elegans* genome (WS220) by using Bowtie (v2.1)^64^ with the following parameter: -q. Uniquely mapping reads that contained no more than one mismatch were used for peak calling and read density calculations. For the calculation of normalized read densities around TSSs, around transcriptional end sites (TESs; the sites where the transcribed region of the mRNA ends and polyadenylation begins), and at DAF-16 peaks, the R package ngs.plot^65^ was used with the following parameters: -R tss -FL 200 (for TSSs), -R gene body -FL 200 (for TESs), or -R bed -E {bed-file of DAF16 peaks} -FL 200 (for DAF-16 peaks). The list of DAF-16 peaks was obtained from^17^. Using this software, read densities were calculated independently for input and IP samples in bins of 60 bp (Fig. 5b–d, Supplementary Figs. 4d and 10a and for the regions outside the gene body in Supplementary Fig. 10b–d) or of 1% of the gene body length (Supplementary Fig. 10b–d). To then obtain the “fold enrichment over input” values which we plot in the figures, we divide the normalized IP read density of every bin by its corresponding input read density. For *p* value calculations determining whether DAF-16 binding to DAF-16 peak regions had significantly changed between the compared conditions, we first calculated the “fold enrichment over input” values in 60 bp bins, as stated above, covering a region from −600 to +600 bp around DAF-16 peak summits. Next, we compared the “fold enrichment over input” values in these bins between conditions using a *t* test^66^. *p* value calculations determining whether RNA Pol II, RNA Pol II(Ser5Phos), or SPT-5 binding to TSS regions had significantly changed between the compared conditions were conducted in identical manner, only that regions from −600 to +600 bp around TSSs were investigated.

To calculate pausing indices from RNA Pol II ChIP-seq data, we first created an input-normalized BigWig file of IP data, using DeepTools^67^ (function BamCompare with the following arguments: extendReads 200, operation ratio, binSize 60, scaleFactorsMethod None, normalizeUsing RPKM). For each gene, mean read coverages were calculated in specific regions (“relaxed” pausing region [−500 to +500 from TSS], “stringent” pausing region [TSS to +500], or the gene body/elongating region [+500 to TES])) using the function mergeByOverlaps from the IRanges package. For each gene, the coverage ratios between the pausing regions and the gene body were calculated independently. Mean ratio values across the gene sets of interest were then used as the index values shown in Supplementary Table 6.

### Gene functional enrichment analysis and annotation

Gene functional enrichments were determined using the DAVID Bioinformatics Resources (v. 6.7)^68^. Annotation clusters identified by DAVID (clusters of related annotation terms) having an enrichment score of ≥1.3 were considered significant and a representative naming for the cluster was derived from the contained Gene Ontology terms.

### Statistical analysis

Lifespan and stress resistance assays were evaluated by Kaplan–Maier and log-rank tests. Statistical analyses for other experiments are stated in the respective methods sections, tables, or figure legends. Analyses were performed using either SPSS (IBM), OASIS^69^, Excel (Microsoft), Graphpad Prism, or R^59^.

### Reporting summary

Further information on research design is available in the Nature Research Reporting Summary linked to this article.

## Supplementary information

Supplementary Information

Peer Review

Reporting Summary

## Data Availability

The high-throughput sequencing data generated and analyzed during this study are available from the authors upon reasonable request as well as from the Sequence Read Archive at NCBI at the following accession code: PRJNA560378. The mass spectrometry data generated and analyzed during this study are available from the authors upon reasonable request as well as from the PeptideAtlas at the following accession code: PASS01428. The source data underlying Figs. 2a–f, 3a–f, 5b, d, 6c–e and Supplementary Figs. 2a–f, 3a–c, 4b–d, 8b, 9b, c, e, f, and 10a are provided as a Source Data file. Noncropped western blot data underlying Supplementary Figs. 7, 8c, and 9a, d are provided in Supplementary Fig. 11.

## Source Data

Source Data
