# Supplementary material for: DAF-16/FOXO requires Protein Phosphatase 4 to initiate transcription of stress resistance and longevity promoting genes
Source: Nat Commun. 2020 Jan 9;11:138. doi: 10.1038/s41467-019-13931-7 (PMC6952425; doi:10.1038/s41467-019-13931-7)
Supplement: Supplementary file 1 — Supplementary Information [file 41467_2019_13931_MOESM1_ESM.pdf]

SUPPLEMENTARY INFORMATION

DAF-16/FOXO requires Protein Phosphatase 4 to initiate transcription of stress resistance and longevity promoting genes

Sen et al.

Sen et al., Supplementary Table 1

C. elegans strains used in this study:

Strain name	Genotype
N2	*wild type*
CB1370	*daf-2(e1370ts)III*
GR1373	*eri-1(mg366ts)IV*
GR1895	*daf-2(e1370ts)III; zIs356[daf-16p::daf-16::GFP; rol-6(su1006)]IV*
GR1899	*daf-2(e1370ts)III; eri-1(mg366ts)IV*
GR1901	*daf-16(mgDf47lf)I; daf-2(e1370ts)III; eri-1(mg366ts)IV*
KW2104	*ckSi5 [spt-5::GFP + unc-119(+)]II; unc-119(ed3lf)III]*
RIE97	*smk-1(tm2993lf)V; Is_pCR266[smk-1ap::smk-1a::LAP(Spep-TEV-mCherry)::smk-1a-3'UTR; Cb_unc-119(+)]*
RIE364	*Ex[pph-4.1P::pph-4.1::gfp; rol-6(su1006)]*
RIE375	*daf-18 (mg198lf)IV; Is_pAD187[smk-1p::GFP::smk-1a; rol-6(su1006)]*
RIE378	*Is_pAD187[smk-1p::GFP::smk-1a; rol-6(su1006)]*
RIE384	*daf-2 (e1370ts)III; Is_pAD187[smk-1p::GFP::smk-1a; rol-6(su1006)]*
RIE388	*daf-2 (e1370ts)III; Ex[pph-4.1P::pph-4.1(R262L)::gfp; rol-6 (su1006)]*
RIE392	*daf-2 (e1370ts)III; Ex[pph-4.1P::pph-4.1::gfp; rol-6(su1006)]*
RIE393	*daf-2 (e1370ts)III; Ex[pph-4.1P::pph-4.1(R262L)::gfp; rol-6 (su1006)]*
RIE400	*daf-2 (e1370ts)III; Ex[pph-4.1P::pph-4.1::gfp; rol-6(su1006)]*
RIE413	*pph-4.1(tm1445lf)III/ hT2[bli-4(e937) let-?(q782) qIs48](I;III)*
RIE415	*daf-2(e1370ts)III; pph-4.1(tm1445lf)III/ hT2[bli-4(e937) let-?(q782) qIs48](I;III)*
RIE500	*ckSi5 [spt-5::GFP + unc-119(+)]II; daf-2(e1370ts)III; unc-119(ed3lf)III*
AS23	*muIs84[(pAD76) sod-3p::GFP + rol-6]; daf-2(e1370ts)III*
CF1553	*muIs84[(pAD76) sod-3p::GFP + rol-6]*
TJ356	*zIs356[daf-16p::daf-16::GFP; rol-6(su1006)]IV*

Sen et al., Supplementary Table 2

MS/MS results of the SMK-1::mCherry purification from wild type animals (independent repeat of Fig. 1b):

Only proteins identified by at least two unique peptides and with a coverage of at least 1.5% are shown.
The shown proteins were entirely absent from control purifications.
Table is sorted by spectral counts.

Sequence name	Protein name	Annotation	Spectral counts	Unique peptides	Coverage [%]	MW [kDa]
F41E6.4	SMK-1	Protein phosphatase 4 regulatory subunit	91	65	57,1	123
D2092.2	PPFR-2	Protein phosphatase 4 regulatory subunit	29	19	69,3	42
Y75B8A.30	PPH-4.1	Protein phosphatase 4 catalytic subunit	27	21	64,0	37
Y48G9A.3	GCN-1	Translational activator	11	11	6,5	291
F01G4.6	F01G4.6	Mitochondrial phosphate carrier protein	4	4	9,4	37
F48E8.5	PAA-1	Protein phosphatase 2A structural subunit	4	3	8,5	66
C27D11.1	EGL-45	Translation initiation factor	4	3	4,0	124
Y71F9AL.17	COPA-1	Coatomer protein complex subunit	4	3	3,5	138
T09B4.9	TIN-44	Mitochondrial import inner membrane translocase subunit	3	3	9,2	49
F38B2.1	IFA-1	Intermediate filament	3	3	7,8	67
Y59A8A.3	Y59A8A.3	Protein of unknown function	3	3	4,6	76
M110.4	IFG-1	Translation initiation factor	3	2	3,6	129
T23E7.2	T23E7.2	Protein of unknown function	3	2	2,7	98
F39B2.10	DNJ-12	Chaperone	2	2	9,2	44
Y49E10.3	PPH-4.2	Protein phosphatase 4 catalytic subunit	2	2	8,7	36
C07A12.4	PDI-2	Protein disulfide isomerase subunit	2	2	7,3	55
C26E1.3	CEH-79	Homeobox protein	2	2	6,1	51
T05C12.7	CCT-1	Cytosolic 'T complex' chaperonin	2	2	5,8	59
F01F1.8	CCT-6	Cytosolic 'T complex' chaperonin	2	2	3,5	59
Y25C1A.5	COPB-1	Coatomer protein complex subunit	2	2	3,0	108
F18E2.3	SCC-3	Cohesin subunit	2	2	2,4	125
C24H11.7	GBF-1	Guanine nucleotide exchange factor	2	2	2,1	223
C34D4.14	HECD-1	E3 ubiquitin protein ligase	2	2	1,7	304
K10B3.10	SPC-1	Spectrin	2	2	1,7	282

Sen et al., Supplementary Table 3

MS/MS results of the SMK-1::GFP purifications from *daf-2(e1370ts)* or *daf-18(mg198lf)* animals:

Only proteins identified by at least five unique peptides in one of the conditions are shown.
Also SPT-4 is shown, although it was identified by less peptides.
The shown proteins were entirely absent from control purifications.
Table is sorted by the proteins' maximal spectral count across both conditions.

Sequence name	Protein name	Annotation	Spectral counts in *daf-2*	Unique peptides in *daf-2*	Coverage in *daf-2* [%]	Spectral counts in *daf-18*	Unique peptides in *daf-18*	Coverage in *daf-18* [%]	MW [kDa]
F41E6.4	SMK-1	Protein phosphatase 4 regulatory subunit	323	70	61	406	84	70	123
D2092.2	PPFR-2	Protein phosphatase 4 regulatory subunit	174	32	83	193	34	79	42
F56A3.4	SPD-5	Coiled-coil protein required for centrosome maturation	127	39	38	176	46	40	135
F48E8.5	PAA-1	Protein phosphatase 2A structural subunit	107	28	51	91	23	42	66
Y75B8A.30	PPH-4.1	Protein phosphatase 4 catalytic subunit	104	14	62	98	14	58	37
T20G5.1	CHC-1	Clathrin heavy chain	42	15	11	71	23	17	191
T10F2.4	PRP-19	Splicing factor	59	16	49	54	14	42	53
Y47D3A.26	SMC-3	Cohesin complex subunit	44	21	21	59	27	28	146
T20F5.6	T20F5.6	Ring finger protein	49	20	32	50	19	30	88
C06A6.2	C06A6.2	Protein of unknown function	31	14	27	37	15	29	75
D1081.8	CDC-5L	CDC5-like protein	35	17	31	26	12	21	86
K08E4.1	SPT-5	Transcription elongation factor, DSIF complex subunit	6	2	3	35	12	13	133
Y49E10.3	PPH-4.2	Protein phosphatase 4 catalytic subunit	32	6	40	33	7	39	36
C26E1.3	CEH-79	Homeobox protein	14	6	22	33	14	45	51
T15H9.2	T15H9.2	Protein of unknown function	18	6	34	32	10	51	29
F28B3.7	HIM-1	Cohesin complex subunit	20	10	9	31	15	13	144
T08A11.2	SFTB-1	Splicing factor	22	11	11	29	14	13	147
ZK688.9	ZK688.9	Regulator of protein phosphatase 2A				29	8	36	33
C18D4.2	FBXA-136	F-box protein				26	12	10	214
F26F12.7	LET-418	NURD chromatin remodeling complex subunit	12	5	3	25	10	7	209
K07A1.12	LIN-53	Chromatin protein	22	8	24	20	7	20	47
F26B1.3	IMA-2	Importin	14	5	17	21	7	22	59
C42D8.2	VIT-2	Vitellogenin	20	9	7				188
F36A2.13	UBR-5	E3 ubiquitin ligase				20	10	4	331
C50B6.2	NASP-2	Chromatin protein	19	8	21	11	5	12	63
K07H8.6	VIT-6	Vitellogenin				19	7	5	193
F25H5.4	EEF-2	Translation elongation factor				19	7	7	95
R07B1.4	GST-36	Prostaglandin D synthase				19	7	38	24
B0393.1	RPS-0	Ribosomal Protein	18	6	30	14	6	27	31
K02F2.2	AHCY-1	S-adenosylhomocysteine hydrolase	18	5	12				48
T05F1.3	RPS-19	Ribosomal Protein	17	5	33	17	6	34	16
C05E4.9	ICL-1	Isocitrate lyase	16	6	7				109
F11G11.2	GST-7	Glutathione S-Transferase				16	6	21	23
Y77E11A.7	Y77E11A.7	Protein of unknown function	10	5	10	16	7	12	71
B0041.4	RPL-4	Ribosomal Protein	15	6	19				39
ZK688.9	ZK688.9	Regulator of protein phosphatase 2A	15	5	25				33
C32E8.2	RPL-13	Ribosomal Protein	15	5	27				24
C07A12.4	PDI-2	Protein disulfide isomerase subunit	14	5	12	15	5	13	55
Y37D8A.2	Y37D8A.2	Phospholipase				15	5	13	65
F10B5.1	RPL-10	Ribosomal Protein				15	5	23	25
T23E7.2	T23E7.2	Protein of unknown function	14	7	11				98
T01C3.6	RPS-16	Ribosomal Protein	14	5	32				16
K08A8.3	COH-1	Cohesin complex subunit				14	5	10	74
C04F6.1	VIT-5	Vitellogenin				14	6	4	186
T16H12.11	HAL-2	Protein involved in meiotic chromosome segregation				14	5	20	36
H20J04.8	MOG-2	Splicing factor				14	5	27	29
F18E2.3	SCC-3	Cohesin complex subunit	12	6	6	14	7	8	125
F36A2.6	RPS-15	Ribosomal Protein	13	5	22				17
P49405	RPL-5	Ribosomal Protein	13	5	25				33
K12G11.3	SODH-1	Sorbitol dehydrogenase	13	5	19				38
Y113G7B.23	SWSN-1	SWI/SNF chromatin remodeling complex subunit	12	5	9	13	5	10	86
W01B11.3	NOL-58	Ribonucleoprotein	12	6	17				55
C23G10.3	RPS-3	Ribosomal Protein				12	5	30	27
C26C6.5	DCP-66	NURD chromatin remodeling complex subunit				11	6	12	79
F39B2.10	DNJ-12	Chaperone				11	5	17	44
Y106G6H.2	PAB-1	Poly(A)-binding protein				11	5	11	72
K02F2.3	TEG-4	Splicing factor				11	5	5	135
R12E2.3	RPN-8	Proteasome subunit	11	5	19				41
ZK1193.5	DVE-1	Transcription factor/regulator				10	5	9	53
Y48A6B.11	RSA-2	Regulator of Spindle Assembly				10	5	8	108
Y48G9A.3	GCN-1	Translational activator				10	5	2	291
D1054.15	PLRG-1	Splicing factor	10	5	14				55
W03F9.10	SFTB-2	Splicing factor	10	5	11	10	5	9	68
T27E9.1	ANT-1.1	ADP/ATP translocase	10	5	17	9	5	15	33
Y71H2AM.20	Y71H2AM.20	Protein phosphatase 2A activator	9	5	19				43
:	:	:	:	:	:	:	:	:	:
:	:	:	:	:	:	:	:	:	:
F54C4.2	SPT-4	Transcription elongation factor, DSIF complex subunit	3	1	8	2	1	8	14

Sen et al., Supplementary Table 4

Summary of the survival data and their statistical evaluation:

Figure 2a, lifespan

Conditions	n	mean survival time [days]	Std.Error	change vs. control RNAi [%]	log-rank test [corr. *p*-value]
daf-2(e1370)III; eri-1(mg366)IV; control RNAi	529	33,63	0,25	n/a	n/a
daf-2(e1370)III; eri-1(mg366)IV; daf-16 RNAi	401	12,94	0,12	-61,5	< 0.001
daf-2(e1370)III; eri-1(mg366)IV; smk-1 RNAi	201	22,69	0,26	-32,5	< 0.001
daf-2(e1370)III; eri-1(mg366)IV; pph-4.1 RNAi	175	30,34	0,40	-9,8	< 0.001
daf-2(e1370)III; eri-1(mg366)IV; pph-4.2 RNAi	199	29,26	0,37	-13,0	< 0.001
daf-2(e1370)III; eri-1(mg366)IV; ppfr-2 RNAi	184	30,41	0,37	-9,6	< 0.001

Figure 2b, lifespan

Conditions	n	mean survival time [days]	Std.Error	change vs. control RNAi [%]	log-rank test [corr. *p*-value]
daf-16(mgDf47)I; daf-2(e1370)III; eri-1(mg366)IV; control RNAi	321	10,49	0,09	n/a	n/a
daf-16(mgDf47)I; daf-2(e1370)III; eri-1(mg366)IV; smk-1 RNAi	91	11,12	0,17	6.0*	0.006*
daf-16(mgDf47)I; daf-2(e1370)III; eri-1(mg366)IV; pph-4.1 RNAi	106	10,66	0,16	1.6*	0.567*
daf-16(mgDf47)I; daf-2(e1370)III; eri-1(mg366)IV; pph-4.2 RNAi	113	10,57	0,14	0.8*	0.905*
daf-16(mgDf47)I; daf-2(e1370)III; eri-1(mg366)IV; ppfr-2 RNAi	73	11,09	0,17	5.7*	0.023*

*: This RNAi clone actually extended the lifespan when compared to control RNAi.

Figure 2c, lifespan

Conditions	n	mean survival time [days]	Std.Error	change vs. control RNAi [%]	log-rank test [corr. *p*-value]
daf-2(e1370)III; eri-1(mg366)IV; control RNAi	92	36,77	0,38	n/a	n/a
daf-2(e1370)III; eri-1(mg366)IV; pph-4.1 RNAi	66	32,68	0,54	-11,1	< 0.001
daf-2(e1370)III; eri-1(mg366)IV; pph-4.2 RNAi	65	31,62	0,70	-14,0	< 0.001
daf-2(e1370)III; eri-1(mg366)IV; pph-4.1/pph-4.2 RNAi	63	28,65	0,57	-22,1	< 0.001
daf-2(e1370)III; eri-1(mg366)IV; smk-1 RNAi	66	22,06	0,39	-40,0	< 0.001

	change [%]			log-rank test [corr. p-value]
daf-2(e1370)III; eri-1(mg366)IV; pph-4.1 RNAi -> daf-2(e1370)III; eri-1(mg366)IV; pph-4.1/pph-4.2 RNAi	-12,3			< 0.001
daf-2(e1370)III; eri-1(mg366)IV; pph-4.2 RNAi -> daf-2(e1370)III; eri-1(mg366)IV; pph-4.1/pph-4.2 RNAi	-9,4			< 0.001

Figure 2d, lifespan

Conditions	n	mean survival time [days]	Std.Error	change vs. control RNAi [%]	log-rank test [corr. *p*-value]
N2; control RNAi	399	13,66	0.11	n/a	n/a
N2; daf-2 RNAi	120	23,93	0.31	75,2	< 0.001
		mean survival time [days]	Std.Error	change vs. control RNAi [%]	log-rank test [corr. *p*-value]
pph-4.1(tm1445)III; control RNAi	110	12,43	0.24	n/a	n/a
pph-4.1(tm1445)III; daf-2 RNAi	74	15,68	0.34	26,1	< 0.001

Figure 2e, lifespan

Conditions	n	mean survival time [days]	Std.Error	*pph-4.1* mutant vs. control [%]	log-rank test [corr. *p*-value]
daf-2 (e1370ts); control RNAi	129	38,90	0,92	n/a	n/a
daf-2(e1370ts)III; pph-4.1(tm1445)III ; control RNAi	113	26,50	0,72	-31,9	< 0.001
		mean survival time [days]	Std.Error	*pph-4.1* mutant vs. control [%]	log-rank test [corr. *p*-value]
daf-2 (e1370ts); daf-16 RNAi	107	13,17	0,22	n/a	n/a
daf-2(e1370ts)III; pph-4.1(tm1445)III ; daf-16 RNAi	120	12,77	0,19	-3,0	0,114

Figure 2f, lifespan

Conditions	n	mean survival time [days]	Std.Error	*pph-4.1(R262L)* vs. control [%]	log-rank test [corr. *p*-value]
daf-2 (e1370)III; Ex[pph-4.1P::pph-4.1::gfp; rol-6 (su1006)]; control RNAi	120	37,43	0,57	n/a	n/a
daf-2 (e1370)III; Ex[pph-4.1P::pph-4.1(R262L)::gfp; rol-6 (su1006)]; control RNAi	125	33,75	0,58	-9,8	< 0.001
		mean survival time [days]	Std.Error	*pph-4.1(R262L)* vs. control [%]	log-rank test [corr. *p*-value]
daf-2 (e1370)III; Ex[pph-4.1P::pph-4.1::gfp; rol-6 (su1006)]; daf-16 RNAi	136	12,26	0,15	n/a	n/a
daf-2 (e1370)III; Ex[pph-4.1P::pph-4.1(R262L)::gfp; rol-6 (su1006)]; daf-16 RNAi	135	11,87	0,18	-3,2	0,136

Supplementary Figure 2a, lifespan

Conditions	n	mean survival time [days]	Std.Error	change vs. control RNAi [%]	log-rank test [corr. *p*-value]
daf-2(e1370)III; eri-1(mg366)IV; control RNAi	529	33,63	0,25	n/a	n/a
daf-2(e1370)III; eri-1(mg366)IV; daf-16 RNAi	401	12,94	0,12	-61,5	< 0.001
daf-2(e1370)III; eri-1(mg366)IV; smk-1 RNAi	201	22,69	0,26	-32,5	< 0.001
daf-2(e1370)III; eri-1(mg366)IV; ppfr-1 RNAi	181	33,71	0,40	0.2*	0.978*
daf-2(e1370)III; eri-1(mg366)IV; ppfr-4 RNAi	182	28,42	0,34	-15,5	< 0.001

*: This RNAi clone actually extended the lifespan when compared to control RNAi.

Supplementary Figure 2b, lifespan

Conditions	n	mean survival time [days]	Std.Error	change vs. control RNAi [%]	log-rank test [corr. *p*-value]
daf-16(mgDf47)I; daf-2(e1370)III; eri-1(mg366)IV; control RNAi	321	10,49	0,09	n/a	n/a
daf-16(mgDf47)I; daf-2(e1370)III; eri-1(mg366)IV; smk-1 RNAi	91	11,12	0,17	6.0*	0.006*
daf-16(mgDf47)I; daf-2(e1370)III; eri-1(mg366)IV; ppfr-1 RNAi	88	10,14	0,12	-3,3	0,021
daf-16(mgDf47)I; daf-2(e1370)III; eri-1(mg366)IV; ppfr-4 RNAi	52	9,02	0,25	-14,0	< 0.001

*: This RNAi clone actually extended the lifespan when compared to control RNAi.

Supplementary Figure 2c, lifespan

Conditions	n	mean survival time [days]	Std.Error	change vs. control RNAi [%]	log-rank test [corr. *p*-value]
eri-1(mg366)IV; control RNAi	325	14,03	0,15	n/a	n/a
eri-1(mg366)IV; pph-4.1 RNAi	220	13,01	0,15	-7,2	< 0.001
eri-1(mg366)IV; pph-4.2 RNAi	241	13,01	0,14	-7,3	< 0.001
eri-1(mg366)IV; pph-4.1/pph-4.2 RNAi	205	12,98	0,16	-7,5	< 0.001

	change [%]			log-rank test [corr. p-value]
eri-1(mg366)IV; pph-4.1 RNAi -> eri-1(mg366)IV; pph-4.1/pph-4.2 RNAi	-0,3			0,983
eri-1(mg366)IV; pph-4.2 RNAi -> eri-1(mg366)IV; pph-4.1/pph-4.2 RNAi	-0,3			0,858

Supplementary Figure 2d, lifespan

Conditions	n	mean survival time [days]	Std.Error	change vs. control RNAi [%]	log-rank test [corr. *p*-value]
daf-16(mgDf47)I; daf-2(e1370)III; eri-1(mg366)IV; control RNAi	73	10,21	0,16	n/a	n/a
daf-16(mgDf47)I; daf-2(e1370)III; eri-1(mg366)IV; pph-4.1 RNAi	70	11,13	0,18	9.0*	< 0.001*
daf-16(mgDf47)I; daf-2(e1370)III; eri-1(mg366)IV; pph-4.2 RNAi	64	11,47	0,20	12.4*	< 0.001*
daf-16(mgDf47)I; daf-2(e1370)III; eri-1(mg366)IV; pph-4.1/pph-4.2 RNAi	71	11,53	0,22	12.9*	< 0.001*

*: This RNAi clone actually extended the lifespan when compared to control RNAi.

	change [%]			log-rank test [corr. p-value]
daf-16(mgDf47)I; daf-2(e1370)III; eri-1(mg366)IV; pph-4.1 RNAi -> daf-16(mgDf47)I; daf-2(e1370)III; eri-1(mg366)IV; pph-4.1/pph-4.2 RNAi	3.5*			0.148*
daf-16(mgDf47)I; daf-2(e1370)III; eri-1(mg366)IV; pph-4.2 RNAi -> daf-16(mgDf47)I; daf-2(e1370)III; eri-1(mg366)IV; pph-4.1/pph-4.2 RNAi	0.5*			0.745*

#: Here combined pph-4.1/pph-4.2 RNAi actually extended the lifespan when compared to the single RNAi.

Supplementary Figure 2e, lifespan

Conditions	n	mean survival time [days]	Std.Error	change vs. control RNAi [%]	log-rank test [corr. *p*-value]
daf-2(e1370ts); control RNAi	117	41,53	0.68	n/a	n/a
daf-2(e1370ts); smk RNAi	107	28,06	0.55	-32,4	< 0.001

pph-4.1(tm1445lf); daf-2(e1370ts); pph-4.2/control RNAi	88	25,27	0.59	-39,2	< 0.001
pph-4.1(tm1445lf); daf-2(e1370ts); pph-4.2/smk-1 RNAi	111	21,90	0.54	-47,3	< 0.001

Supplementary Figure 2f, lifespan

Conditions	n	mean survival time [days]	Std.Error	*pph-4.1(R262L)* vs. control [%]	log-rank test [corr. *p*-value]
daf-2 (e1370)III; Ex[pph-4.1P::pph-4.1::gfp; rol-6 (su1006)]; control RNAi	114	37,11	0.50	n/a	n/a
daf-2 (e1370)III; Ex[pph-4.1P::pph-4.1(R262L)::gfp; rol-6 (su1006)]; control RNAi	145	33,39	0.50	-10,0	< 0.001
		mean survival time [days]	Std.Error	*pph-4.1(R262L)* vs. control [%]	log-rank test [corr. *p*-value]
daf-2 (e1370)III; Ex[pph-4.1P::pph-4.1::gfp; rol-6 (su1006)]; daf-16 RNAi	134	12,60	0.15	n/a	n/a
daf-2 (e1370)III; Ex[pph-4.1P::pph-4.1(R262L)::gfp; rol-6 (su1006)]; daf-16 RNAi	135	12,64	0.18	0,3	1

Figure 3a, oxidative stress survival

Conditions	n	mean survival time [hours]	Std.Error	change vs. control RNAi [%]	log-rank test [corr. *p*-value]
daf-2(e1370)III; eri-1(mg366)IV; control RNAi	99	99,64	3,39	n/a	n/a
daf-2(e1370)III; eri-1(mg366)IV; daf-16 RNAi	68	25,68	0,50	-74,2	< 0.001
daf-2(e1370)III; eri-1(mg366)IV; smk-1 RNAi	107	67,20	2,64	-32,6	< 0.001
daf-2(e1370)III; eri-1(mg366)IV; pph-4.1/pph-4.2 RNAi	115	86,98	2,18	-12,7	0,002

Figure 3b, oxidative stress survival

Conditions	n	mean survival time [hours]	Std.Error	change vs. control RNAi [%]	log-rank test [corr. *p*-value]
daf-16(mgDf47)I; daf-2(e1370)III; eri-1(mg366)IV; control RNAi	79	23,91	0,48	n/a	n/a
daf-16(mgDf47)I; daf-2(e1370)III; eri-1(mg366)IV; smk-1 RNAi	83	26,61	0,62	11,3	< 0.001
daf-16(mgDf47)I; daf-2(e1370)III; eri-1(mg366)IV; pph-4.1/pph-4.2 RNAi	87	25,48	0,62	6,5	0,019

Figure 3c, UV stress survival

Conditions	n	mean survival time [hours]	Std.Error	change vs. control RNAi [%]	log-rank test [corr. *p*-value]
daf-2(e1370)III; eri-1(mg366)IV; control RNAi	96	156,69	5,05	n/a	n/a
daf-2(e1370)III; eri-1(mg366)IV; daf-16 RNAi	128	80,43	2,92	-48,7	< 0.001
daf-2(e1370)III; eri-1(mg366)IV; smk-1 RNAi	51	74,37	7,13	-52,5	< 0.001
daf-2(e1370)III; eri-1(mg366)IV; pph-4.1/pph-4.2 RNAi	78	112,58	6,34	-28,1	< 0.001

Figure 3d, UV stress survival

Conditions	n	mean survival time [hours]	Std.Error	change vs. control RNAi [%]	log-rank test [corr. *p*-value]
daf-16(mgDf47)I; daf-2(e1370)III; eri-1(mg366)IV; control RNAi	56	59,33	4,30	n/a	n/a
daf-16(mgDf47)I; daf-2(e1370)III; eri-1(mg366)IV; smk-1 RNAi	54	67,72	5,81	14,1	0,030
daf-16(mgDf47)I; daf-2(e1370)III; eri-1(mg366)IV; pph-4.1/pph-4.2 RNAi	52	66,51	3,40	12,1	0,924

Figure 3e, heat stress survival

Conditions	n	mean survival time [hours]	Std.Error	change vs. control RNAi [%]	log-rank test [corr. *p*-value]
daf-2(e1370)III; eri-1(mg366)IV; control RNAi	144	58,46	0,98	n/a	n/a
daf-2(e1370)III; eri-1(mg366)IV; daf-16 RNAi	65	24,19	0,45	-58,6	< 0.001
daf-2(e1370)III; eri-1(mg366)IV; smk-1 RNAi	76	60,73	1,40	3,9	0,240
daf-2(e1370)III; eri-1(mg366)IV; pph-4.1/pph-4.2 RNAi	107	53,98	1,40	-7,7	0,277

Figure 3f, heat stress survival

Conditions	n	mean survival time [hours]	Std.Error	change vs. control RNAi [%]	log-rank test [corr. *p*-value]
daf-16(mgDf47)I; daf-2(e1370)III; eri-1(mg366)IV; control RNAi	127	24,52	0,38	n/a	n/a
daf-16(mgDf47)I; daf-2(e1370)III; eri-1(mg366)IV; smk-1 RNAi	60	23,46	0,45	-4,3	0,022
daf-16(mgDf47)I; daf-2(e1370)III; eri-1(mg366)IV; pph-4.1/pph-4.2 RNAi	133	23,63	0,32	-3,6	0,197

Supplementary Figure 3a, oxidative stress survival

Conditions	n	mean survival time [hours]	Std.Error	change vs. control RNAi [%]	log-rank test [corr. *p*-value]
eri-1(mg366)IV; control RNAi	87	32,75	0,87	n/a	n/a
eri-1(mg366)IV; daf-16 RNAi	76	22,16	0,39	-32,3	< 0.001
eri-1(mg366)IV; smk-1 RNAi	82	31,81	1,39	-2,9	0,242
eri-1(mg366)IV; pph-4.1/pph-4.2 RNAi	88	32,09	0,68	-2,0	0,194

Supplementary Figure 3b, UV stress survival

Conditions	n	mean survival time [hours]	Std.Error	change vs. control RNAi [%]	log-rank test [corr. *p*-value]
eri-1(mg366)IV; control RNAi	104	105,66	3,23	n/a	n/a
eri-1(mg366)IV; daf-16 RNAi	104	79,85	3,67	-24,4	< 0.001
eri-1(mg366)IV; smk-1 RNAi	104	78,55	4,01	-25,7	< 0.001
eri-1(mg366)IV; pph-4.1/pph-4.2 RNAi	88	96,42	3,87	-8,7	0,061

Supplementary Figure 3c, heat stress survival

Conditions	n	mean survival time [hours]	Std.Error	change vs. control RNAi [%]	log-rank test [corr. *p*-value]
eri-1(mg366)IV; control RNAi	130	26,49	0,41	n/a	n/a
eri-1(mg366)IV; daf-16 RNAi	72	23,63	0,45	-10,8	< 0.001
eri-1(mg366)IV; smk-1 RNAi	84	25,30	0,71	-4,5	0,549
eri-1(mg366)IV; pph-4.1/pph-4.2 RNAi	100	25,26	0,61	-4,6	0,438

Figure 6c, lifespan

Conditions	n	mean survival time [days]	Std.Error	change vs. control RNAi [%]	log-rank test [corr. *p*-value]
eri-1(mg366ts); daf-2 (e1370ts); control RNAi	83	37,32	0,45	n/a	n/a
eri-1(mg366ts); daf-2 (e1370ts); spt-5 RNAi	92	30,51	0,64	-18,2	< 0.001
				change vs. control RNAi [%]	log-rank test [corr. *p*-value]
eri-1(mg366ts); control RNAi	126	14,76	0,19	n/a	n/a
eri-1(mg366ts); spt-5 RNAi	141	15,61	0,24	5,8	0,005
				change vs. control RNAi [%]	log-rank test [corr. *p*-value]
daf-16(mgDf47)I; daf-2(e1370)III; eri-1(mg366)IV; control RNAi	65	10,28	0,16	n/a	n/a
daf-16(mgDf47)I; daf-2(e1370)III; eri-1(mg366)IV; spt-5 RNAi	170	10,50	0,09	2,1	0,228

Figure 6d, oxidative stress survival

Conditions	n	mean survival time [days]	Std.Error	change vs. control RNAi [%]	log-rank test [corr. *p*-value]
eri-1(mg366ts); daf-2 (e1370ts); control RNAi	68	2,48	0,10	n/a	n/a
eri-1(mg366ts); daf-2 (e1370ts); daf-16 RNAi	81	1,22	0,03	-50,8	< 0.001
eri-1(mg366ts); daf-2 (e1370ts); spt-5 RNAi	66	1,86	0,06	-25,0	< 0.001
				change vs. control RNAi [%]	log-rank test [corr. *p*-value]
daf-16(mgDf47)I; daf-2(e1370)III; eri-1(mg366)IV; control RNAi	140	1,02	0,02	n/a	n/a
daf-16(mgDf47)I; daf-2(e1370)III; eri-1(mg366)IV; spt-5 RNAi	167	0,84	0,02	-17,6	< 0.001

Figure 6e, heat stress survival

Conditions	n	mean survival time [days]	Std.Error	change vs. control RNAi [%]	log-rank test [corr. *p*-value]
eri-1(mg366ts); daf-2 (e1370ts); control RNAi	69	2,90	0,07	n/a	n/a
eri-1(mg366ts); daf-2 (e1370ts); daf-16 RNAi	90	1,18	0,05	-59,5	< 0.001
eri-1(mg366ts); daf-2 (e1370ts); spt-5 RNAi	84	2,93	0,09	0,9	0,270
				change vs. control RNAi [%]	log-rank test [corr. *p*-value]
daf-16(mgDf47)I; daf-2(e1370)III; eri-1(mg366)IV; control RNAi	109	0,83	0,02	n/a	n/a
daf-16(mgDf47)I; daf-2(e1370)III; eri-1(mg366)IV; spt-5 RNAi	187	0,87	0,02	4,8	0,051

Supplementary Figure 8b, lifespan

Conditions	n	mean survival time [hours]	Std.Error	change vs. control RNAi [%]	log-rank test [corr. *p*-value]
eri-1(mg366ts); daf-2 (e1370ts); control RNAi	83	37,32	0,45	n/a	
eri-1(mg366ts); daf-2 (e1370ts); daf-16 RNAi	86	16,69	0,31	-55,3	< 0.001
eri-1(mg366ts); daf-2 (e1370ts); smk-1 RNAi	84	21,22	0,26	-43,1	< 0.001
eri-1(mg366ts); daf-2 (e1370ts); spt-4 RNAi	80	37,63	0,50	0.8*	1,000
eri-1(mg366ts); daf-2 (e1370ts); spt-5 RNAi	92	30,51	0,64	-18,2	< 0.001
eri-1(mg366ts); daf-2 (e1370ts); emb-5 RNAi	90	37,89	0,44	1.5*	1,000

*: This RNAi clone actually extended the lifespan when compared to control RNAi.

Sen et al., Supplementary Table 5

Basic quality metrics of our ChIP-seq samples:

Figure 5

RNA Pol II IP from *daf-2* animals on control RNAi			
Read length		50	single-ended
Illumina mean quality scores at all bases were above		25	
Number of total reads	input	19021564	
	ip	22972055	
Coverage [fold]	input	9,48	
	ip	11,45	
% unique reads	input	84,80	
	ip	78,32	
% unique reads in 10 Mio reads	input	92,01	
	ip	90,56	
Mapping rate for reads	input	>99%	
	ip	>99%	

RNA Pol II IP from *daf-2* animals on *smk-1* RNAi			
Read length		50	single-ended
Illumina mean quality scores at all bases were above		26	
Number of total reads	input	10412387	
	ip	13329835	
Coverage [fold]	input	5,19	
	ip	6,65	
% unique reads	input	89,96	
	ip	82,29	
% unique reads in 10 Mio reads	input	90,36	
	ip	86,71	
Uniquely mapped reads with no more than 1 mismatch	input	>99%	
	ip	>99%	

RNA Pol II Ser5Phos IP from *daf-2* animals on control RNAi			
Read length		50	single-ended
Illumina mean quality scores at all bases were above		26	
Number of total reads	input	19021564	
	ip	10745046	
Coverage [fold]	input	9,48	
	ip	5,36	
% unique reads	input	84,80	
	ip	89,28	
% unique reads in 10 Mio reads	input	92,01	
	ip	90,02	
Uniquely mapped reads with no more than 1 mismatch	input	>99%	
	ip	>99%	

RNA Pol II Ser5Phos IP from *daf-2* animals on *smk-1* RNAi			
Read length		50	single-ended
Illumina mean quality scores at all bases were above		26	
Number of total reads	input	10412387	
	ip	10465322	
Coverage [fold]	input	5,19	
	ip	5,22	

% unique reads	input	89,96	
	ip	89,37	
% unique reads in 10 Mio reads	input	90,36	
	ip	89,84	
Uniquely mapped reads with no more than 1 mismatch	input	>99%	
	ip	>99%	

Supplementary Figure 3

GFP IP from *daf-2; DAF-16::GFP* animals on control RNAi

Read length		50	single-ended
Illumina mean quality scores at all bases were above		25	
Number of total reads	input	16791633	
	ip	29509495	
Coverage [fold]	input	8,37	
	ip	14,71	
% unique reads	input	78,36	
	ip	38,66	
% unique reads in 10 Mio reads	input	87,11	
	ip	79,21	
Mapping rate for reads	input	>99%	
	ip	>99%	

GFP IP from *daf-2; DAF-16::GFP* animals on *smk-1* RNAi

Read length		50	single-ended
Illumina mean quality scores at all bases were above		25	
Number of total reads	input	19148696	
	ip	34218469	
Coverage [fold]	input	9,55	
	ip	17,06	
% unique reads	input	77,52	
	ip	29,95	
% unique reads in 10 Mio reads	input	88,26	
	ip	79,53	
Mapping rate for reads	input	>99%	
	ip	>99%	

Figure 7 and Supplementary Figure 7

RNA Pol II IP from *daf-2; SPT-5::GFP* animals on control RNAi

Read length		50	single-ended
Illumina mean quality scores at all bases were above		25	
Number of total reads	input	26814365	
	ip	24401878	
Coverage [fold]	input	13,37	
	ip	12,17	
% unique reads	input	64,07	
	ip	38,53	
% unique reads in 10 Mio reads	input	86,60	
	ip	74,81	
Mapping rate for reads	input	>99%	
	ip	>99%	

RNA Pol II IP from *daf-2; SPT-5::GFP* animals on *smk-1* RNAi

Read length		50	single-ended
Illumina mean quality scores at all bases were above		25	
Number of total reads	input	23275130	
	ip	22682876	
Coverage [fold]	input	11,60	
	ip	11,31	
% unique reads	input	60,42	
	ip	30,48	
% unique reads in 10 Mio reads	input	82,99	
	ip	69,35	
Mapping rate for reads	input	>99%	
	ip	>99%	

GFP IP from *daf-2; SPT-5::GFP* animals on control RNAi

Read length		50	single-ended
Illumina mean quality scores at all bases were above		25	
Number of total reads	input	26814365	
	ip	18438971	
Coverage [fold]	input	13,37	
	ip	9,19	
% unique reads	input	64,07	
	ip	56,22	
% unique reads in 10 Mio reads	input	86,60	
	ip	76,26	
Mapping rate for reads	input	>99%	
	ip	>99%	

GFP IP from *daf-2; SPT-5::GFP* animals on *smk-1* RNAi

Read length		50	single-ended
Illumina mean quality scores at all bases were above		25	
Number of total reads	input	23275130	
	ip	19128664	
Coverage [fold]	input	11,60	
	ip	9,54	
% unique reads	input	60,42	
	ip	46,89	
% unique reads in 10 Mio reads	input	82,99	
	ip	72,23	
Mapping rate for reads	input	>99%	
	ip	>99%	

GFP IP from *daf-2; SMK-1::GFP* animals

Read length		50	single-ended
Illumina mean quality scores at all bases were above		25	
Number of total reads	input	7893369	
	ip	10512400	
Coverage [fold]	input	3,94	
	ip	5,24	
% unique reads	input	95,18	
	ip	88,48	
% unique reads in 10 Mio reads	input	93,90	
	ip	89,04	
Mapping rate for reads	input	>99%	
	ip	>99%	

Sen et al., Supplementary Table 6

Pausing indices for the different gene sets in Figure 5:

Two pausing indices were calculated, resembling the average read density ratios of either a "relaxed" (between -500 and +500 from the TSS) or a "stringent" (between 0 and +500 from the TSS) window for paused RNA Pol II to elongating (between +500 and the TES) RNA Pol II. Indices are shown for the indicated conditions and gene sets.

condition	pausing index	all genes (n=20389)	genes activated by DAF-16 (n=960)	genes co-activated by DAF-16 and SMK-1 (n=150)
daf-2	stringent	1,31	1,21*	1,21 [a1]
daf-2	relaxed	1,41	1,31*	1,46 [b1]
daf-2; daf-16 RNAi	stringent	1,34	1,26*	1,25
daf-2; daf-16 RNAi	relaxed	1,44	1,36	1,45
daf-2; smk-1 RNAi	stringent	1,35	1,22*	1,16* [a2]
daf-2; smk-1 RNAi	relaxed	1,44	1,31*	1,41 [b2]

*: significantly different from the value for "all genes", looking at the same type of index under the same conditions (p<0.05, t-test)

[#]: significantly different from the value for "genes activated by DAF-16", looking at the same type of index under the same conditions (p<0.05, t-test)

[a1,a2]: There is no significant difference between these two values (p<0.05, test).

[b1,b2]: There is no significant difference between these two values (p<0.05, test).

Sen et al., Supplementary Table 7

MS/MS results of the unbiased phosphoproteomics in *daf-2(e1370ts)* animals:

only significantly phosphorylation differences are shown (p<0.05)
data is based on triplicate experiments
§: Fold change in phosphorylation of the site mentioned under "Phosphosite", when comparing *smk-1(RNAi)* -treated animals to *control(RNAi)* -treated animals
*: Describes the significance of the phosphorylation change observed for the "Phosphosite".
#: The site mentioned in the column "Phosphosite" is shown in BOLD letters.

Peptides containing phosphorylations that were more abundant in the absence of *smk-1* (under *smk-1(RNAi)*):

Sequence name	Gene Name	Protein Name	Annotation	Protein ID	Phosphosite	Fold change [log2]§	p value*	Phospho(STY) probabilities#
T11B7.4	alp-1	ALP-1	LIM domain-binding protein	G5EFL5	319	2,933	0,00018211	AAYHPQVNT(0.001)ARPV**S(0.998)**VS(0.995)PAPS(0.005)AGSK
C14F11.5	hsp-43	HSP-43	Heat shock protein	H2KYS1	300	1,179	0,00058028	ITTSG**S(1)**YNTAGNANLHEER
K08E4.1	spt-5	SPT-5	Transcription elongation factor, DSIF complex subunit	Q21338	671	1,119	0,00088262	MA**S(1)**PNPMAS(1)PR
C02F5.8	tsp-1	TSP-1	Tetraspanin-1	P34285	227	1,953	0,00196769	TYKY(0.001)**S(0.995)**Y(0.004)EPR
C43E11.1	acin-1	ACIN-1	Apoptotic chromatin condensation inducer	P91156	182	1,287	0,00218349	VDAETAQE**S(0.978)**DS(0.022)PQEAEK
K08E4.1	spt-5	SPT-5	Transcription elongation factor, DSIF complex subunit	Q21338	882	2,356	0,00248500	TPAYG**S(1)**ADGAR
T12D8.8	hip-1	HIP-1	Hsp-70 Interacting Protein homolog	G5EE04	68	5,790	0,00261989	ETPAAEA**T(1)**PEPEIPKPEEIPFPK
W03D8.6	itx-1	ITX-1	InTestinal NeureXin-like	A0A078BPJ4	1422	2,414	0,00265960	D**S(0.992)**PLY(0.007)S(0.042)NIRPPT(0.477)S(0.438)PIHAS(0.043)VPVPPPR
K08E4.1	spt-5	SPT-5	Transcription elongation factor, DSIF complex subunit	Q21338	677	1,127	0,00268686	MAS(1)PNPMA**S(1)**PR
C14F11.5	hsp-43	HSP-43	Heat Shock Protein	H2KYS1	241	1,596	0,00272190	GPNS(0.999)PIHVQT(0.506)EHDGRS(0.432)VS(0.399)**S(0.663)**R
C14F11.5	hsp-43	HSP-43	Heat Shock Protein	H2KYS1	232	1,596	0,00272190	GPNSPIHVQ**T(0.545)**EHDGRS(0.433)VS(0.511)S(0.511)R
Y53G8AR.6	Y53G8AR.6	Y53G8AR.6	Protein of unknown function	Q9N3G0	89	1,081	0,00295559	LLG**S(1)**VDGAEEHK
F42C5.10	ifo-1	IFO-1	Intermediate Filament Organize	Q20330	805	0,860	0,00305455	AN**S(0.997)**RPT(0.254)T(0.749)PGLEIK
W03F9.10	sftb-2	SFTB-2	Splicing Factor ThreeB (3b) Subunit homolog	O16997	473	1,708	0,00313417	ES(0.001)**S(0.999)**VLGTDTPAAAYHIIPEK
C39H7.4	C39H7.4	C39H7.4	Protein of unknown function	Q18550	94	0,861	0,00313799	NYAPI**S(0.905)**GY(0.001)S(0.042)T(0.042)T(0.01)PLR
B0563.4	tmbi-4	TMBI-4	Transmembrane BAX inhibitor motif-containing protein 4	Q11080	23	0,813	0,00336674	VNLLS(1)DHDD**S(1)**DDDEVHVK
F10G7.1	tag-151	TAG-151	Pre-rRNA-processing protein TSR1 homolog	Q19329	774	0,985	0,00336839	S(0.139)RVE**S(0.85)**IS(0.011)LVNEDAMEE
F42C5.10	ifo-1	IFO-1	Intermediate Filament Organize	Q20330	809	0,790	0,00381335	ANS(0.995)RPT(0.053)**T(0.952)**PGLEIK
F40F11.2	mig-38	MIG-38	Protein of unknown function	A6ZJ71	1173	0,962	0,00419619	IKA**S(1)**PAET(0.204)T(0.771)PT(0.024)K
F40F11.2	mig-38	MIG-38	Protein of unknown function	A6ZJ71	1178	0,962	0,00419619	IKAS(1)PAET(0.204)**T(0.771)**PT(0.024)K
W06H8.8	ttn-1	TTN-1	Ortholog of titin	G4SLH0-3	13852	1,503	0,00477620	S(0.459)S(0.541)AIF**S(0.997)**DDEQS(0.002)ISSK
ZK617.1	unc-22	UNC-22	Twitchin	Q23551	584	4,162	0,00500978	ELLGQVGES(0.003)DDEV**S(0.79)**ES(0.194)IS(0.012)ELPSFAGGK
M01F1.4	M01F1.4	M01F1.4	Protein of unknown function	Q5CZ47	798	1,171	0,00519973	PTALYT(1)PAP**S(1)**PR
T07F8.4	T07F8.4	T07F8.4	Protein of unknown function	P91437	55	0,660	0,00524782	DLEDEGEM**S(1)**IEELQR
T24D11.1	eat-17	EAT-17	Ortholog of human Evi5 (ecotropic viral integration site 5)	U4PBB2	367	0,667	0,00525169	IPTLDDLAEEG**S(0.998)**AT(0.002)ETDELRPK
W02A2.6	rec-8	REC-8	Meiotic recombination protein rec-8	Q9XUB3-2	404	0,754	0,00543410	**T(0.501)**ART(0.501)PT(0.998)PIK
W02A2.6	rec-8	REC-8	Meiotic recombination protein rec-8	Q9XUB3-2	407	0,754	0,00543410	T(0.501)AR**T(0.501)**PT(0.998)PIK
W02A2.6	rec-8	REC-8	Meiotic recombination protein rec-8	Q9XUB3-2	409	0,754	0,00543410	T(0.501)ART(0.501)P**T(0.998)**PIK
T04A8.14	emb-5	EMB-5	Transcription elongation factor	P34703	139	3,783	0,00603960	RPNHEDDDLL**S(1)**ERGS(1)DDGDR
T04A8.14	emb-5	EMB-5	Transcription elongation factor	P34703	143	3,783	0,00603960	RPNHEDDDLLS(1)ERG**S(1)**DDGDR
C25A1.10	dao-5	DAO-5	Dauer or Aging adult Overexpression	Q564W7	283	3,746	0,00615745	KADL**S(0.658)**S(0.345)DFS(0.997)DDEAPAK
JC8.2	JC8.2	JC8.2	Protein of unknown function	Q9NAQ2	56	3,721	0,00623854	ELIEGAIPESTDNETELDDDEE**S(1)**DNEK
ZK617.1	unc-22	UNC-22	Twitchin	Q23551	923	3,624	0,00657006	RG**S(1)**VFGELR
F13E6.1	F13E6.1	F13E6.1	Uncharacterized protein F13E6.1	P55326	181	0,669	0,00707836	GP**S(1)**QTGTPVAEEAK
C14F11.5	hsp-43	HSP-43	Heat Shock Protein	H2KYS1	334	0,663	0,00733374	VE**S(1)**PVS(0.788)T(0.167)T(0.037)T(0.008)GILR
F08F1.8	tth-1	TTH-1	Tetra THymosin (four thymosin repeat protein)	O17389	113	0,684	0,00738607	NVLP**S(0.999)**PT(0.001)DVAR
C14F11.5	hsp-43	HSP-43	Heat Shock Protein	H2KYS1	288	0,939	0,00781663	T(0.003)**Y(0.997)**SPVTPR
C14F11.5	hsp-43	HSP-43	Heat Shock Protein	H2KYS1	238	1,184	0,00829315	GPNSPIHVQTEHDGR**S(0.995)**VS(0.62)S(0.385)R
C53A5.3	hda-1	HDA-1	Histone deacetylase 1	O17695	400	0,909	0,00831887	PIPEDALSALNDD**S(1)**LIADQANPDK
Y48B6A.6	efhd-1	EFHD-1	EF HanD calcium binding protein	Q9U2A6	223	0,713	0,00842420	SDE**S(1)**IEEEER
T27A3.4;C10G11.9	spch-3;spch-2	SPCH-3;SPCH-2	SPerm CHromatin enriched	P91497	81	0,836	0,00901552	S(0.207)PS(0.793)LVLVIS(0.021)G**S(0.845)**PS(0.133)PR
K11E8.1	unc-43	UNC-43	Calcium/calmodulin-dependent protein kinase type II	G5EDZ5	498	2,953	0,00922233	ESSES(0.007)**S(0.991)**QT(0.002)IDDNDSEK
F52E1.13	lmd-3	LMD-3	LysM Domain (peptidoglycan binding) protein	Q3Y409	251	3,021	0,00936618	NEEVAFEAGSVDS(0.018)NH**S(0.982)**PK
B0334.15	emc-5	EMC-5	EMC Endoplasmic Membrane protein Complex (yeast EMC) homolog	E1B6R8	142	2,987	0,00957847	VSEVIQT(0.001)EEDS(0.007)ADD**S(0.993)**QE
F01G4.1	swsn-4	SWSN-4	SWI/SNF nucleosome remodeling complex component	G5EF53	1108	1,029	0,00980659	EVVDQ**T(1)**PNQR
F08F1.8	tth-1	TTH-1	Tetra THymosin (four thymosin repeat protein)	O17389	128	0,846	0,01008254	TLQMAA**S(1)**FDK
K12D12.1	top-2	TOP-2	Probable DNA topoisomerase 2	Q23670	1477	1,608	0,01077819	AAGS(1)DDEDDE**S(1)**FVVAPR

F15G9.1	F15G9.1	F15G9.1	Protein of unknown function	Q10033	70	1,003	0,01131911	YSTDLGK**S(0.788)**T(0.212)DK
W06H8.8	ttn-1	TTN-1	Ortholog of titin	G4SLH0-3	1377	1,068	0,01145988	VN**S(1)**HEAMEPIEATNLLLNTALQLK
Y37E11AL.3	Y37E11AL.3	Y37E11AL.3	Protein of unknown function	H2KZH7	272	0,818	0,01175447	IIENYQLEVA**S(1)**EIR
Y53G8AR.9	Y53G8AR.9	Y53G8AR.9	Protein of unknown function	Q9N3F7	242	0,931	0,01253226	YG**S(1)**DDRLMELEK
Y69A2AR.21	Y69A2AR.21	Y69A2AR.21	Protein of unknown function	Q95XJ1	9	0,895	0,01286263	NNS(1)D**S(1)**DEDIESLTNHETSLEAAAK
K08E4.1	spt-5	SPT-5	Transcription elongation factor, DSIF complex subunit	Q21338	904	0,889	0,01300936	TPAY(0.002)G**S(0.789)**MDNS(0.209)R
C25A1.10	dao-5	DAO-5	Dauer or Aging adult Overexpression	Q564W7	287	2,537	0,01315335	KADLS(0.658)S(0.345)DF**S(0.997)**DDEAPAK
W09G12.7	W09G12.7	W09G12.7	Protein of unknown function	O45198	112	2,521	0,01331580	RKPAE**S(1)**S(1)DDEVVVVVVK
W09G12.7	W09G12.7	W09G12.7	Protein of unknown function	O45198	113	2,521	0,01331580	RKPAES(1)**S(1)**DDEVVVVVVK
T05C3.5	dnj-19	DNJ-19	DNaJ domain (prokaryotic heat shock protein)	O16303	421	2,465	0,01390817	GGDAYNED**S(1)**DEEQHGGHHGQGVR
M03C11.8	M03C11.8	M03C11.8	Chromatin remodeller	G5EDG2	133	0,569	0,01396957	REDS(1)E**T(1)**PEPEMK
H28G03.2	H28G03.2	H28G03.2	Protein of unknown function	H2L0D2	131	0,531	0,01460697	T(1)PPEEPE**S(1)**PK
M03C11.8	M03C11.8	M03C11.8	Chromatin remodeller	G5EDG2	131	0,566	0,01462019	RED**S(1)**ET(1)PEPEMK
Y48C3A.12	Y48C3A.12	Y48C3A.12	Protein of unknown function	Q9U290	137	0,535	0,01482813	KT(0.051)**S(0.919)**NS(0.03)LLLPDEK
C18C4.10	klc-2	KLC-2	Kinesin light chain	P46822	524	0,542	0,01529739	NSMTTST(0.029)**S(0.956)**QT(0.015)GLK
T24B8.7	T24B8.7	T24B8.7	Ubiquitin carboxyl-terminal hydrolase	N1NVC2	34	2,337	0,01541273	KR**S(0.85)**AS(0.85)PAT(0.299)ANQS(0.001)SWGDNPAK
Y43C5A.5	thk-1	THK-1	Thymidine kinase	F3Y5P8	238	2,328	0,01552523	SDG**S(1)**VS(1)PPRK
F42C5.10	ifo-1	IFO-1	Intermediate Filament Organize	Q20330	409	0,517	0,01563800	LP**S(1)**PAPPKT(1)PEPAYTVLNR
F37E3.1	ncbp-1	NCBP-1	Nuclear cap-binding protein subunit 1	O01763	673	0,817	0,01578326	IKEEDDEE**S(1)**DIK
C50B6.2	nasp-2	NASP-2	NASP (human Nuclear Autoantigenic Sperm Protein) homolog	O17687	544	0,866	0,01606581	RPA**S(1)**EEAPEETK
Y48A6B.3	Y48A6B.3	Y48A6B.3	Putative H/ACA ribonucleoprotein complex subunit 2-like protein	Q9XXD4	16	0,831	0,01614942	RNLDET(0.005)MNES(0.787)T(0.27)V**S(0.938)**EANGDATAPTTEK
M03A1.6	ipla-1	IPLA-1	Intracelllar PhosphoLipase A family	G5EEM9	131	0,782	0,01657549	S(1)GPVAPPRPSG**T(1)**PVAPQR
F42C5.10	ifo-1	IFO-1	Intermediate Filament Organize	Q20330	415	0,499	0,01661658	**T(1)**PEPAYTVLNR
F57B10.9	spg-20	SPG-20	Human SPG (spastic paraplegia)	O44735	461	0,461	0,01727666	KG**S(1)**RLDQEVPALELK
C29A12.3	lig-1	LIG-1	DNA ligase 1	Q27474	732	1,069	0,01736228	VE**S(1)**PS(1)PIRK
C14F11.5	hsp-43	HSP-43	Heat Shock Protein	H2KYS1	274	0,922	0,01769223	NRL**S(1)**PNDVNITR
W03F9.10	sftb-2	SFTB-2	Splicing Factor ThreeB (3b) Subunit homolog	O16997	472	2,167	0,01783588	GKE**S(0.914)**S(0.887)VLGT(0.134)DT(0.063)PAAAY(0.002)HIIPEK
F32B4.4	F32B4.4	F32B4.4	Protein of unknown function	F5GUF6	301	1,182	0,01825648	VEAS(0.178)T(0.822)PQA**T(0.822)**PS(0.178)K
T09A5.10	lin-5	LIN-5	Spindle apparatus protein lin-5	P45970	694	0,452	0,01855539	SIAP**S(1)**VDDNEFDK
M03A1.6	ipla-1	IPLA-1	Intracelllar PhosphoLipase A family	G5EEM9	120	0,695	0,01869285	**S(1)**GPVAPPRPSGT(1)PVAPQR
M106.2	M106.2	M106.2	Protein of unknown function	Q09592	465	2,111	0,01874567	GAAIDEP**S(1)**IDTNQR
T26A5.5	jhdm-1	JHDM-1	JmjC domain-containing histone demethylation protein 1	A0A0K3AUW2	354	2,101	0,01891816	EY(0.056)RP**S(0.944)**PNAAPT(0.006)PS(0.814)PS(0.179)HHQK
C09G5.6	bli-1	BLI-1	Cuticle collagen	Q09457	623	0,506	0,01894572	RPNY(0.003)EP**S(0.997)**AEVAPPR
F07A5.7	unc-15	UNC-15	Paramyosin	P10567	30	0,875	0,01915837	**S(0.562)**PS(0.438)QAAFGAPFGSMSVADLGSLTR
F10B5.8	F10B5.8	F10B5.8	Protein of unknown function	Q9U3K2	470	0,828	0,01948964	S(0.001)L**S(0.999)**LDPNPAK
T05F1.6	hsr-9	HSR-9	Protein of unknown function	Q7JKP6	901	0,895	0,01971781	GVDLVS(0.295)**S(0.675)**RGGS(0.675)AS(0.355)PAEEEEK
C25A1.10	dao-5	DAO-5	Dauer or Aging adult Overexpression	Q564W7	692	0,779	0,02090745	KGSYGGGPINQSINS(0.002)IKF**S(0.999)**DS(0.999)DD
C25A1.10	dao-5	DAO-5	Dauer or Aging adult Overexpression	Q564W7	694	0,779	0,02090745	KGSYGGGPINQSINS(0.002)IKFS(0.999)D**S(0.999)**DD
C07E3.3	C07E3.3	C07E3.3	Protein of unknown function	Q17786	150	0,641	0,02140067	RYDQVPIDP**S(1)**PELQR
Y71F9B.16	dnj-30	DNJ-30	DNaJ domain (prokaryotic heat shock protein)	A0A078BS22	58	1,966	0,02149553	RR**S(0.992)**T(0.008)EALVQAAEK
F10C1.2	ifb-1	IFB-1	Intermediate filament protein ifb-1	Q19289	31	1,962	0,02157449	Q**S(0.992)**GNY(0.007)VSGGNGAGSGGR
C43C3.1	ifp-1	IFP-1	Intermediate filament protein	Q09501	454	0,486	0,02192055	DA**S(1)**PIRPSYTPYQQESR
F09G2.9	attf-2	ATTF-2	AT hook Transcription Factor family	O17406	240	0,788	0,02236258	GRPPGPNA**T(1)**PK
F08D12.1	F08D12.1	F08D12.1	Signal recognition particle subunit SRP72	P91240	555	0,479	0,02238550	NYNSAV**T(1)**PDPER
C39H7.4	C39H7.4	C39H7.4	Protein of unknown function	Q18550	117	0,674	0,02333828	FVDT(0.003)**S(0.813)**T(0.184)DYGNIGR
M04B2.1	mep-1	MEP-1	MOG interacting and ectopic P-granules protein 1	Q21502	33	0,428	0,02403931	SAGES(0.001)S(0.048)**S(0.905)**DS(0.045)EPDTIEQLK
T05F1.6	hsr-9	HSR-9	Protein of unknown function	Q7JKP6	316	1,844	0,02427655	RVSATVSST(0.001)PS(0.003)S(0.045)N**T(0.95)**PR
T03F1.9	hcp-4	HCP-4	HoloCentric chromosome binding Protein	G5EDA3	312	1,822	0,02485480	NQ**S(0.925)**S(0.075)EMQGTVTVAK
Y38E10A.17	Y38E10A.17	Y38E10A.17	Protein of unknown function	Q9NAJ4	504	0,472	0,02485487	**S(1)**VGFGAQQYGGSVFAK
F45G2.3	exo-1	EXO-1	EXOnuclease	O62245	330	0,699	0,02507801	EVLMTGVDDLEEVIDLD**S(1)**PKK
F47B10.2	haly-1	HALY-1	Histidine ammonia-lyase	Q20502	675	1,807	0,02523813	S(0.008)MIPIS(0.997)DDEE**S(0.996)**IE
K03A1.2	lron-7	LRON-7	eLRR (extracellular Leucine-Rich Repeat) Only	H2L0D5	56	0,441	0,02537016	KAELTAAQDEDDEG**S(1)**K
Y71D11A.2	smr-1	SMR-1	SMN (Survival of Motor Neuron protein) Related	Q95Y51	223	0,567	0,02575068	**S(0.896)**S(0.104)QFIFK
T08B2.5	T08B2.5	T08B2.5	Protein of unknown function	Q86NJ9	473	0,684	0,02629433	GRR**S(0.988)**EEPS(0.005)T(0.005)S(0.001)SSFSSVPLAPR
R08D7.1	R08D7.1	R08D7.1	BUD13 homolog	P30640	99	0,499	0,02708681	VIKPEPL**S(1)**PDNS(1)PPRGK
F29G6.3	hpo-34	HPO-34	Protein of unknown function	K8FDY0	1081	0,726	0,02715922	KD**S(1)**FAVR
R05D3.7	unc-116	UNC-116	Kinesin heavy chain	P34540	408	1,146	0,02730604	**S(0.999)**IAPAPPMLT(0.45)S(0.402)T(0.113)T(0.034)GPIT(0.001)DEEK
C47B2.4	pbs-2	PBS-2	Proteasome subunit beta type	O62102	276	0,560	0,02731618	YDVVESMDI**T(1)**H
C04C11.2	arrd-25	ARRD-25	ARRestin Domain protein	Q17624	299	0,423	0,02732707	LLE**S(1)**PST(0.003)AT(0.996)LR
C04C11.2	arrd-25	ARRD-25	ARRestin Domain protein	Q17624	304	0,423	0,02732707	LLES(1)PST(0.003)A**T(0.996)**LR
Y17G7A.1	hmg-12	HMG-12	High mobility group	G5EDQ2	91	1,963	0,02754773	RENS(0.905)ANDS(0.095)PANTNDVDIV**S(0.663)**S(0.337)PVK

C15C7.5	C15C7.5	C15C7.5	Protein of unknown function	Q18012	101	0,574	0,02790829	MSQNQ**S(1)**EDEEFKK
F27C1.6	F27C1.6	F27C1.6	Protein of unknown function	P91280	5	0,678	0,02826847	S(1)DD**S(1)**DLDDVAHQK
C50B6.2	nasp-2	NASP-2	NASP (human Nuclear Autoantigenic Sperm Protein) homolog	O17687	475	0,644	0,02844152	SDIQ**S(1)**EIK
F32E10.4	ima-3	IMA-3	Importin subunit alpha-3	Q19969	54	1,440	0,02848246	NINTQIEDDS(0.387)E**T(0.546)**S(0.356)T(0.356)T(0.356)PPGPFDANLLR
F18E2.3	scc-3	SCC-3	Cohesin subunit	Q19555	9	1,868	0,02864504	SETPTDQ**S(1)**PQR
D2045.1	atx-2	ATX-2	human ATX (ataxin) related	G5ED29	474	0,467	0,02872849	HQEDDNV**S(1)**VT(0.003)S(0.997)ENDSVITSK
Y43H11AL.3	pqn-85	PQN-85	Nipped-B-like protein pqn-85	Q95XZ5	632	0,659	0,02899239	DR**S(1)**PT(0.992)PEDVIES(0.008)R
Y43H11AL.3	pqn-85	PQN-85	Nipped-B-like protein pqn-85	Q95XZ5	634	0,659	0,02899239	DRS(0.999)P**T(0.999)**PEDVIES(0.002)R
B0228.4	cpna-2	CPNA-2	Copine family protein 2	Q09221	774	0,426	0,02947775	LHYEQTADKT(1)P**S(1)**PIPLEK
B0228.4	cpna-2	CPNA-2	Copine family protein 2	Q09221	772	0,426	0,02947775	LHYEQTADK**T(1)**PS(1)PIPLEK
Y62H9A.4	Y62H9A.4	Y62H9A.4	Protein of unknown function	Q9XWT4	40	0,763	0,02967905	Y(0.002)S(0.002)**T(0.536)**S(0.452)DS(0.007)S(0.002)DSSENELK
F54C1.8	F54C1.8	F54C1.8	Protein of unknown function	P91325	24	0,668	0,02984591	HEN**S(1)**NVS(1)VLEIVDEHNIGPNQK
F27C8.5	F27C8.5	F27C8.5	Protein of unknown function	Q19838	295	0,712	0,03019451	T(0.245)R**S(0.746)**EIS(0.009)AIDK
C14F11.5	hsp-43	HSP-43	Heat Shock Protein	H2KYS1	240	2,478	0,03047391	GPNSPIHVQTEHDGRS(0.995)V**S(0.62)**S(0.385)R
C14F11.5	hsp-43	HSP-43	Heat Shock Protein	H2KYS1	210	0,648	0,03047627	MIPIEGAGHH**S(1)**PR
F37C4.5	F37C4.5	F37C4.5	Protein F37C4.5	O44400	331	0,664	0,03053970	M**S(1)**YAAVK
H19M22.2	let-805	LET-805	Protein of unknown function	Q9UB28	4318	0,459	0,03135464	ELDS(0.003)L**S(0.579)**RS(0.419)PDVR
K08A2.4	K08A2.4	K08A2.4	Protein of unknown function	Q9N5J4	207	0,809	0,03137266	HAAEINEMER**S(1)**PEGS(0.994)EGGS(0.006)VEK
K08A2.5	K08A2.5	K08A2.5	Protein of unknown function	Q9N5J4	211	0,809	0,03137266	HAAEINEMERS(1)PEG**S(0.994)**EGGS(0.006)VEK
W02C12.3	hlh-30	HLH-30	Helix Loop Helix	H2KZZ3	157	1,239	0,03189877	AGS(0.052)GHS(0.19)G**S(0.734)**PIT(0.025)IPNAMSNNFR
K10D2.3	cid-1	CID-1	Caffeine Induced Death (S. pombe Cid) homolog	Q09409	799	0,818	0,03196438	ANVIIL**S(1)**EDS(0.061)S(0.939)EIDEK
Y46G5A.31	gsy-1	GSY-1	Glycogen [starch] synthase	Q9U2D9	658	0,912	0,03211765	DNEGKVPS(0.533)AAT(0.592)**S(0.875)**R
F54D11.4	F54D11.4	F54D11.4	Protein of unknown function	J3K004	95	0,394	0,03216211	RFS(1)P**S(0.999)**PVT(0.001)K
D2092.2	ppfr-2	PPFR-2	Protein Phosphatase Four Regulatory subunit	P91198	320	0,906	0,03277797	AA**S(1)**PVS(0.001)S(0.003)PKS(0.997)PVIQDQS(1)PKK
F44B9.6	lin-36	LIN-36	Protein lin-36	P34427	507	0,876	0,03304214	LAT(0.006)S(0.994)A**T(0.705)**NS(0.295)PIKK
F01G4.3	skih-2	SKIH-2	SKI (yeast SuperKIller) Helicase homolog	Q19103	217	0,379	0,03310694	KS(0.011)ES(0.99)IE**S(1)**EETPEDDKK
F56G4.4	F56G4.4	F56G4.4	Protein of unknown function	Q9XUY1	352	1,555	0,03354954	EH**S(0.656)**S(0.689)T(0.656)PPPEELQEQLEEPPK
F56G4.5	F56G4.5	F56G4.5	Protein of unknown function	Q9XUY1	353	1,555	0,03354954	EHS(0.651)**S(0.699)**T(0.651)PPPEELQEQLEEPPK
F56G4.6	F56G4.6	F56G4.6	Protein of unknown function	Q9XUY1	354	1,555	0,03354954	EHS(0.656)S(0.689)**T(0.656)**PPPEELQEQLEEPPK
T05B11.3	clic-1	CLIC-1	Clathrin LIght Chain	P90961	2	0,680	0,03383105	**S(1)**DPVADFLAR
E01H11.1	pkc-2	PKC-2	Protein kinase C-like 2	P90980	8	0,621	0,03394007	S(0.996)LS(0.004)TNS(0.002)**S(0.998)**VKEDEAQR
F56D5.6	F56D5.6	F56D5.6	Protein of unknown function	I2HAJ8	162	0,793	0,03449137	LDYTD**S(1)**GES(1)DEDLK
F56D5.6	F56D5.6	F56D5.6	Protein of unknown function	I2HAJ8	165	0,793	0,03449137	LDYTDS(1)GE**S(1)**DEDLK
M110.4	ifg-1	IFG-1	Initiation Factor 4G (eIF4G) family	B5BM23	807	0,619	0,03463734	RS(0.805)**T(0.616)**S(0.115)QNS(0.314)NT(0.15)DKT(0.001)DDELTEEEK
M110.4	ifg-1	IFG-1	Initiation Factor 4G (eIF4G) family	B5BM23	806	0,619	0,03463734	R**S(0.805)**T(0.616)S(0.115)QNS(0.314)NT(0.15)DKT(0.001)DDELTEEEK
F42G8.4	pmk-3	PMK-3	Mitogen-activated protein kinase	W6RTQ3	113	0,422	0,03490804	DTSLTQ**Y(0.994)**VQT(0.006)R
F25E2.4	ifd-2	IFD-2	Intermediate filament protein ifd-2	Q19782	424	0,925	0,03519184	TTVQTHVTYNAPPPPLPQ**S(1)**GPR
M03D4.1	zen-4	ZEN-4	Kinesin-like protein	G5EC43	740	0,626	0,03557330	LTHQEVDDEGNI**S(0.821)**T(0.179)NIVK
F42C5.10	ifo-1	IFO-1	Intermediate Filament Organize	Q20330	271	0,385	0,03596069	**S(0.999)**LS(0.001)NLFSVDTR
T24B8.7	T24B8.7	T24B8.7	Ubiquitin carboxyl-terminal hydrolase	N1NVC2	36	0,628	0,03597157	S(0.028)A**S(0.972)**PATANQSSWGDNPAK
T05F1.6	hsr-9	HSR-9	Protein of unknown function	Q7JKP6	907	0,410	0,03601676	GGS(1)A**S(1)**PAEEEEK
Y67D2.7	Y67D2.7	Y67D2.7	Protein of unknown function	Q9BKQ7	118	1,482	0,03669265	ISGAT(1)PI**S(1)**EIREPAPPPR
Y67D2.8	Y67D2.8	Y67D2.8	Protein of unknown function	Q9BKQ7	115	1,482	0,03669265	ISGA**T(1)**PIS(1)EIREPAPPPR
Y40B1B.8	slc-25A46	SLC-25A46	SLC (SoLute Carrier) homolog	Q9U2J0	399	0,701	0,03673023	ATQGHPVVQSSQI**T(1)**PPIS(1)AK
C04G2.8	spch-1	SPCH-1	SPerm CHromatin enriched	Q17626	70	0,613	0,03712203	S(0.059)P**S(0.941)**LVVVISPSPR
R08D7.1	R08D7.1	R08D7.1	BUD13 homolog	P30640	103	0,377	0,03732032	VIKPEPLS(1)PDN**S(1)**PPRGK
Y116A8C.36	itsn-1	ITSN-1	ITSN (intersectin) family	Q9U2T9	268	0,770	0,03764712	**S(1)**ANNT(1)PELEPGAEPPQK
C08B11.3	swsn-7	SWSN-7	SWI/SNF nucleosome remodeling complex component	Q09441	1230	0,705	0,03830678	HQPIQQHIPS(0.026)QP**S(0.945)**PLVQT(0.541)T(0.488)PVR
C08B11.3	swsn-7	SWSN-7	SWI/SNF nucleosome remodeling complex component	Q09441	1235	0,705	0,03830678	HQPIQQHIPS(0.026)QPS(0.945)PLVQ**T(0.541)**T(0.488)PVR
C24H11.7	gbf-1	GBF-1	Protein of unknown function	G5EGS5	1322	0,923	0,03835137	VD**S(1)**AGS(1)LLGAQK
Y48B6A.6	efhd-1	EFHD-1	EF HanD calcium binding protein	Q9U2A6	355	0,565	0,03981607	VEA**S(0.984)**PT(0.016)PLPK
F15G9.1	F15G9.1	F15G9.1	Uncharacterized protein F15G9.1	Q10033	186	1,415	0,04000962	EVS(0.465)S(0.556)AA**S(0.979)**LFANDNGNETENR
ZK512.8	ZK512.8	ZK512.8	Uncharacterized protein	P34646	101	0,538	0,04010258	KEIS(0.997)VT(0.002)S(0.001)AH**S(0.994)**NS(0.005)S(0.001)LPVR
C04G2.8	spch-1	SPCH-1	SPerm CHromatin enriched	Q17626	68	0,621	0,04037081	**S(0.997)**PS(0.003)LVVVISPSPR
H28G03.2	H28G03.2	H28G03.2	Protein of unknown function	H2L0D2	124	0,357	0,04084194	**T(1)**PPEEPES(1)PK
C25A1.3	tag-72	TAG-72	mRNA cap guanine-N7 methyltransferase	Q9XVS1	355	1,399	0,04088537	TEEEPAT(0.001)T(0.001)KPVAES(0.411)E**S(0.588)**EQK
D2089.1	rsp-7	RSP-7	Probable splicing factor, arginine/serine-rich 7	O01159	451	0,680	0,04112618	NEEIGNGDVVMA**S(1)**E
Y32F6A.3	pap-1	PAP-1	Poly-A Polymerase	Q9U2P3	538	0,573	0,04173038	T(0.116)T(0.132)S(0.154)T(0.05)S(0.234)S(0.314)VPT(0.084)**T(0.74)**PT(0.176)GLAAPK
Y62H9A.4	Y62H9A.4	Y62H9A.4	Protein of unknown function	Q9XWT4	43	1,371	0,04242319	YST(0.001)SD**S(0.927)**S(0.07)DS(0.002)SENELK
R06F6.1	cdl-1	CDL-1	Histone RNA hairpin-binding protein	Q09599	183	0,664	0,04265323	LFT(0.001)NGG**S(0.905)**DS(0.068)S(0.02)S(0.006)VASSPSR
F27C1.6	F27C1.6	F27C1.6	Protein of unknown function	P91280	2	0,565	0,04271601	**S(1)**DDS(1)DLDDVAHQK

F09G2.9	attf-2	ATTF-2	AT hook Transcription Factor family	O17406	204	0,367	0,04284243	KDAAEAPAADEE**S(1)**EAAEEK
Y17G7A.1	hmg-12	HMG-12	High mobility group	G5EDQ2	76	0,476	0,04284690	REN**S(1)**ANDS(1)PANTNDVDIVS(0.254)S(0.746)PVK
F54E12.2	F54E12.2	F54E12.2	Protein of unknown function	G5EEW5	176	0,439	0,04324875	**S(0.995)**PEVS(0.004)T(0.001)GLFETALSPIAK
Y75B8A.22	tim-1	TIM-1	Protein timeless homolog	G5EDN3	1286	0,472	0,04337303	KLPAAEDD**S(1)**DLEEDAVIYK
F53G12.1	rab-11.1	RAB-11.1	Ras-related protein	O01803	54	1,353	0,04348533	SI**S(1)**VEGK
F11C3.3	unc-54	UNC-54	Myosin-4	P02566	1936	0,543	0,04372562	S(0.999)RA**S(0.998)**AS(0.003)VAPGLQS(0.165)S(0.152)AS(0.422)AAVIRS(0.253)PS(0.007)R
C18B2.5	C18B2.5	C18B2.5	Protein of unknown function	Q18076	4	1,329	0,04495248	**S(0.529)**T(0.529)AVS(0.942)INLDDVFGNLPALTSATNVK
C18B2.6	C18B2.6	C18B2.6	Protein of unknown function	Q18076	5	1,329	0,04495248	S(0.529)**T(0.529)**AVS(0.942)INLDDVFGNLPALTSATNVK
W02B8.2	W02B8.2	W02B8.2	Protein of unknown function	Q9XVH4	1246	0,374	0,04580057	GI**S(1)**PFNTLK
F29G6.3	hpo-34	HPO-34	Protein of unknown function	Q93636	497	0,804	0,04612891	ADNSSSVDIIGVS(0.177)**S(0.823)**LR
Y55B1AR.1	lec-6	LEC-6	Galectin	Q9N384	42	1,309	0,04619671	LVLLPT(0.04)**S(0.921)**ADS(0.04)R
C09E8.1	C09E8.1	C09E8.1	Protein of unknown function	H2KYM8	751	0,377	0,04624920	EA**S(1)**EVDEAELPR
F11D5.1	F11D5.1	F11D5.1	Protein of unknown function	Q27GT9	391	0,547	0,04631505	YDNL**S(1)**PAVK
F15G9.1	F15G9.1	F15G9.1	Uncharacterized protein F15G9.1	Q10033	172	0,682	0,04775396	ES(0.002)**S(0.998)**PVS(0.166)S(0.834)PVK
Y75B7AL.4	rga-4	RGA-4	Rho GTPase Activating protein	Q965S4	959	0,428	0,04796407	**S(1)**IDETPTALR
C50B6.2	nasp-2	NASP-2	NASP (human Nuclear Autoantigenic Sperm Protein) homolog	O17687	40	0,723	0,04811669	VAMT(0.002)NVAEET(0.232)**T(0.724)**PS(0.052)T(0.093)DS(0.724)AT(0.133)T(0.039)NEK
F15G9.1	F15G9.1	F15G9.1	Uncharacterized protein F15G9.1	Q10033	119	1,279	0,04823919	EVLQESQI**S(1)**DFVEEDDDQQTEVEEVK
H20J04.8	mog-2	MOG-2	Probable U2 small nuclear ribonucleoprotein A'	Q9BLB6	192	0,454	0,04853753	SVHTEDPSEIEPNEN**S(0.801)**S(0.199)GGGAR
C02C6.1	dyn-1	DYN-1	Dynamin GTPase	P39055-2	819	0,537	0,04914213	VP**T(0.987)**PS(0.013)NGAPEIPARPQVPK
F18E2.3	scc-3	SCC-3	Cohesin subunit	Q19555	32	1,262	0,04946003	VNYTDMAAGNN**S(1)**VEK

Peptides containing phosphorylations that were observed only in the absence of *smk-1* (under *smk-1(RNAi)*)

Sequence name	Gene Name	Protein Name	Annotation	Protein ID	Phosphosite	Phospho(STY) probabilities[#]
C04C11.2	arrd-25	ARRD-25	ARRestin Domain protein	Q17624	301	LLES(0.007)P**S(0.922)**T(0.646)AT(0.425)LR
C18B2.5	C18B2.5	C18B2.5	Protein of unknown function	Q18076	45	TTASS(0.007)V**S(0.725)**T(0.268)VR
C23F12.1	fln-2	FLN-2	FiLamiN (actin binding protein) homolog	A0A0K3ASN4	421	GVS(0.001)NT(0.003)S(0.016)FG**S(0.98)**VR
C44C10.9	C44C10.9	C44C10.9	Protein of unknown function	J7SF86	116	E**Y(1)**AVSQNYLR
C47E8.7	unc-112	UNC-112	Protein unc-112	Q18685	199	GQS(1)PAL**S(0.56)**QS(0.44)GHIFNAHEMGTLPR
C53C9.2	C53C9.2	C53C9.2	Protein of unknown function	Q09936	308	EWEET(0.089)KPPG**S(0.824)**AS(0.066)S(0.022)VDPFGHYK
F01G4.3	skih-2	SKIH-2	Ski2 like RNA helicase	Q19103	212	KS(0.576)ES(0.442)IE**S(0.981)**EET(0.001)PEDDKK
F19F10.9	F19F10.9	F19F10.9	Protein of unknown function	O01524	792	T(0.005)N**S(0.995)**YDTPLGTLDK
F53A2.7	acaa-2	ACAA-2	ACetyl-CoA Acyltransferase 2 homolog	O45552	215	GEE**S(1)**FEIDEHPR
F53B3.3	F53B3.3	F53B3.3	Protein of unknown function	Q20712	418	ENREEY(0.001)END**S(0.999)**PEMAPR
F53H1.1	F53H1.1	F53H1.1	Protein of unknown function	U4PBN2	128	KKEELDLQSEQ**S(1)**DEK
F54D11.2	sumv-2	SUMV-2	SUppressor of synthetic MultiVulva	Q22992	466	ARE**S(0.973)**IS(0.027)HLFDAEMDASIK
F59C12.3	F59C12.3	F59C12.3	Protein of unknown function	Q21036	110	RAS(0.431)A**S(0.569)**GDQALDVPTLSQTNR
R166.5	mnk-1	MNK-1	MAP kinase iNtegrating Kinase (MNK) homolog	Q22005	578	**S(1)**VGIAIEK
T05F1.6	hsr-9	HSR-9	Protein of unknown function	Q7JKP6	420	SAASS(0.042)**S(0.944)**AT(0.01)S(0.003)S(0.001)AVPTPR
T10F2.4	prp-19	PRP-19	pre-mRNA processing factor 19	Q10051	148	VDDDV**S(0.984)**IDES(0.016)EDQQGLSEAILAK
T19A5.2	gck-1	GCK-1	Germinal Center Kinase family	A0A0K3ASW2	455	VSS(0.009)QV**S(0.819)**PS(0.173)K
Y40B1A.3	Y40B1A.3	Y40B1A.3	Protein of unknown function	E1NZ12	318	**S(1)**MDGRPS(1)PEHGAIIGENR
Y48B6A.6	efhd-1	EFHD-1	EF HanD calcium binding protein	Q9U2A6	402	DS(0.073)**S(0.927)**LPPPPPPKPETPLAIR

Sen et al., Supplementary Table 8

Enrichments of TATA-boxes and initiator elements at various gene sets:

Gene set	Number of genes	genes w/ motif information (Maxwell et al., Cell Reports 2014)	Overlap with TATA motif containing genes	fraction [%]	*p*-value (hypergeometric test)
all genes	20389	5063	1161	22,9	
genes co-activated by DAF-16 and SMK-1	150	66	28	42,4	2.47×10^{-4}
genes activated by DAF-16 but not activated by SMK-1	810	234	6	2,6	not significant

Gene set	Number of genes	genes w/ motif information (Maxwell et al., Cell Reports 2014)	Overlap with Inr motif containing genes	fraction [%]	*p*-value (hypergeometric test)
all genes	20389	5063	1582	31,2	
genes co-activated by DAF-16 and SMK-1	150	66	18	27,3	not significant
genes activated by DAF-16 but not activated by SMK-1	810	234	4	1,7	not significant

Sen et al., Supplementary Table 9

Comparisons of our differential gene expression data to published datasets:

published dataset	published in	our dataset	fraction of the genes in the published dataset that overlaps with our dataset	significant overlap? (*p* -value < 0.05; hypergeometric test)
genes upregulated in *daf-2(e1370)* vs wild type (N2)	ref. 2	genes upregulated in *eri-1(mg366); daf-2(1370)* vs *eri-1(mg366)*	55%	yes
genes downregulated in *daf-2(e1370)* vs wild type (N2)	ref. 2	genes downregulated in *eri-1(mg366); daf-2(1370)* vs *eri-1(mg366)*	52%	yes
genes upregulated in *daf-2(e1370)* vs *daf-2(e1370); daf-16(mgDf47)*	ref. 2	genes upregulated in *eri-1(mg366); daf-2(1370)* vs *eri-1(mg366); daf-2(1370); daf-16(RNAi)*	58%	yes
genes downregulated in *daf-2(e1370)* vs *daf-2(e1370); daf-16(mgDf47)*	ref. 2	genes downregulated in *eri-1(mg366); daf-2(1370)* vs *eri-1(mg366); daf-2(1370); daf-16(RNAi)*	57%	yes
genes upregulated in *daf-2* vs *daf-2; daf-16*	ref. 3	genes upregulated in *eri-1(mg366); daf-2(1370)* vs *eri-1(mg366); daf-2(1370); daf-16(RNAi)*	45%	yes
genes downregulated in *daf-2* vs *daf-2; daf-16*	ref. 3	genes downregulated in *eri-1(mg366); daf-2(1370)* vs *eri-1(mg366); daf-2(1370); daf-16(RNAi)*	36%	yes
genes upregulated in *daf-2* vs *daf-2; skn-1*	ref. 4	genes upregulated in *eri-1(mg366); daf-2(1370)* vs *eri-1(mg366); daf-2(1370); smk-1(RNAi)*	11%	yes
genes downregulated in *daf-2* vs *daf-2; skn-1*	ref. 4	genes downregulated in *eri-1(mg366); daf-2(1370)* vs *eri-1(mg366); daf-2(1370); smk-1(RNAi)*	9%	no
genes upregulated in *daf-2(e1370)* vs *daf-2(e1370); hlh-30(tm1978)*	ref. 5	genes upregulated in *eri-1(mg366); daf-2(1370)* vs *eri-1(mg366); daf-2(1370); smk-1(RNAi)*	44%	yes
genes downregulated in *daf-2(e1370)* vs *daf-2(e1370); hlh-30(tm1978)*	ref. 5	genes downregulated in *eri-1(mg366); daf-2(1370)* vs *eri-1(mg366); daf-2(1370); smk-1(RNAi)*	57%	yes

Sen et al., Supplementary Figure 1

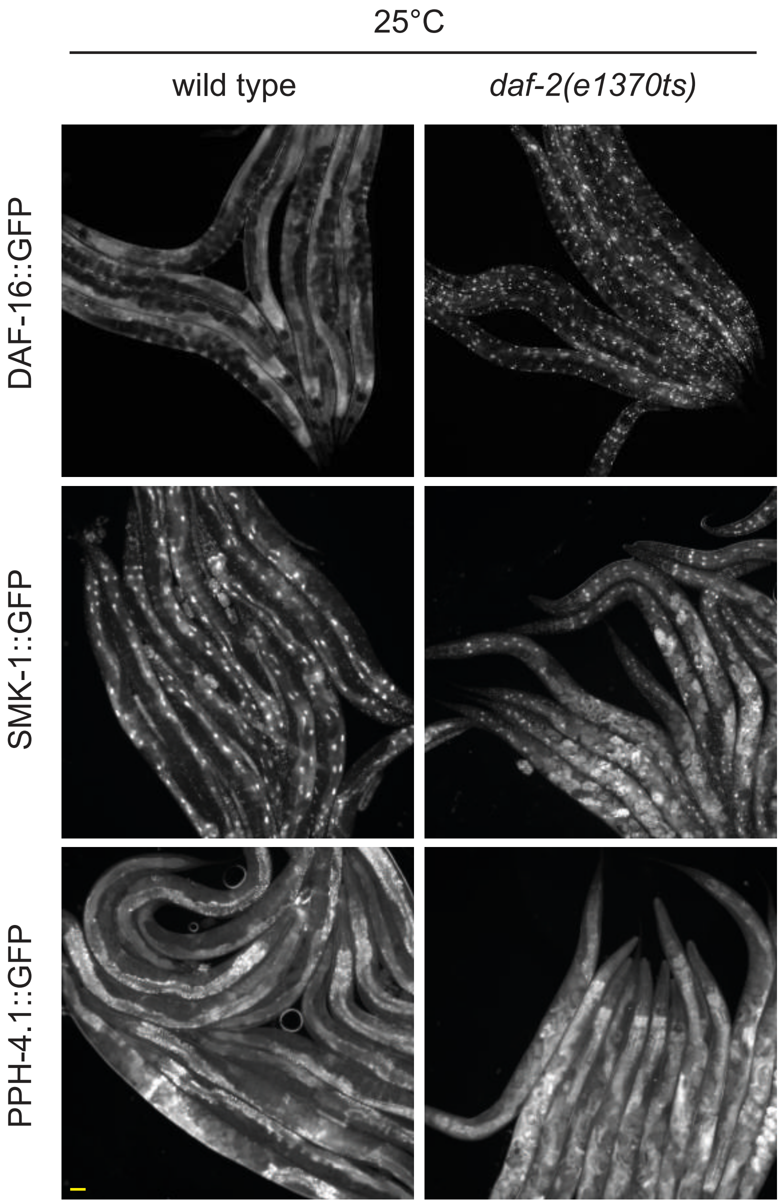

Supplementary Fig. 1 - Localization of PP4^{SMK-1} in adult animals

Localization studies of DAF-16::GFP, SMK-1::GFP and PPH-4.1::GFP in wild type and in *daf-2(e1370ts)* mutant day 1 adult animals. Worms were synchronized by bleaching, grown from the L1 stage at 15°C, and then shifted at the L4 stage to 25°C. After 16-24 hours the GFP signal was recorded in day 1 adult animals (yellow scale bar: 50 µm).

Sen et al., Supplementary Figure 2

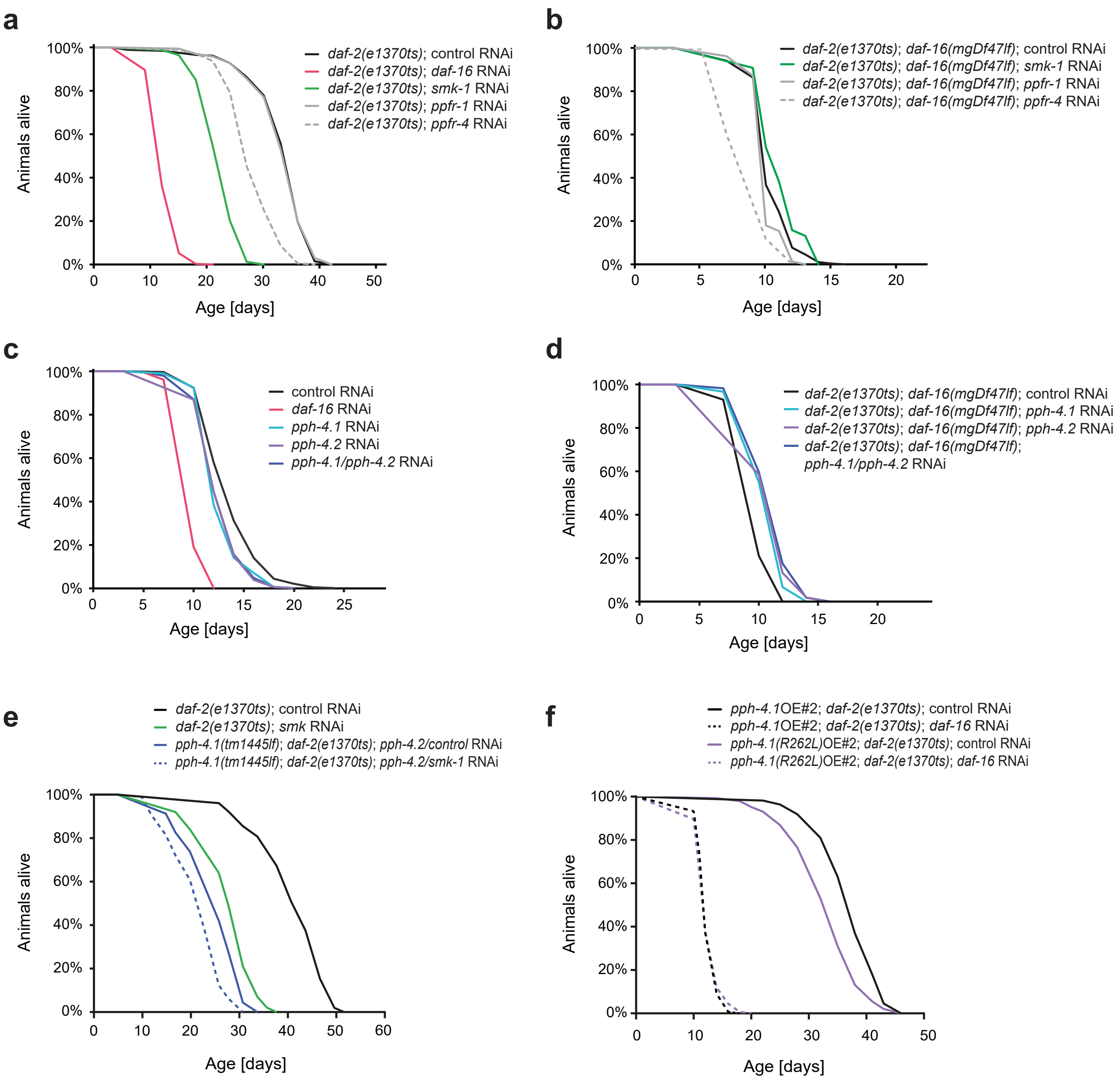

Supplementary Fig. 2 - Additional lifespan data exploring the role of PP4 subunits and the complex's catalytic activity

Animals of indicated genotypes were grown from the L1 stage on the indicated RNAi bacteria at 15°C. At the L4 stage the animals were shifted to 25°C to fully inactivate *daf-2(e1370ts)* and their lifespan was monitored. All strains used in this figure harbored the *eri-1(mg366ts)* mutation to yield better knockdown efficiencies[1]. **a, b** Lifespan phenotypes caused by PP4 subunits that were not found to be part of PP4^{SMK-1} in Figure 1a,b, namely *ppfr-1* and *ppfr-4*, in various genetic backgrounds. Knockdown of *ppfr-4* but not *ppfr-1* shortened the lifespan of *daf-2(e1370ts)* mutant animals (a). However, the lifespan-shortening effect of *ppfr-4* RNAi was still present in *daf-2(e1370ts); daf-16(mgDf47lf)* double mutant animals, showing that this phenotype is DAF-16-independent (b). **c, d** The lifespan-shortening effects of individual or combined loss of the PP4^{SMK-1} catalytic subunit paralogs *pph-4.1* and *pph-4.2* in wild type animals were only mild (c), and they were entirely absent in *daf-2(e1370ts); daf-16(mgDf47lf)* double mutant animals (d), showing clear correlation with the level of DAF-16 activity in these genetic backgrounds. **e** Lifespan phenotypes caused either by RNAi of *smk-1* in *daf-2(e1370ts)* animals, by *pph-4.2* RNAi in *daf-2(e1370ts); pph-4.1(tm1445lf)* animals, or by combined RNAi of *smk-1* and *pph-4.2* in *daf-2(e1370ts); pph-4.1(tm1445lf)* animals, to test for epista-sis between the catalytic subunits PPH-4.1/PPH-4.2 and the regulatory subunit SMK-1 of PP4^{SMK-1}. **f** Lifespan analy-ses of the second independent transgenic lines for *daf-2(e1370ts)* mutant animals ectopically expressing either cata-lytically active PPH-4.1 or catalytically dead PPH-4.1(R262L). For detailed statistics see Supplementary Table 4. Source data are provided as a Source Data file.

Sen et al., Supplementary Figure 3

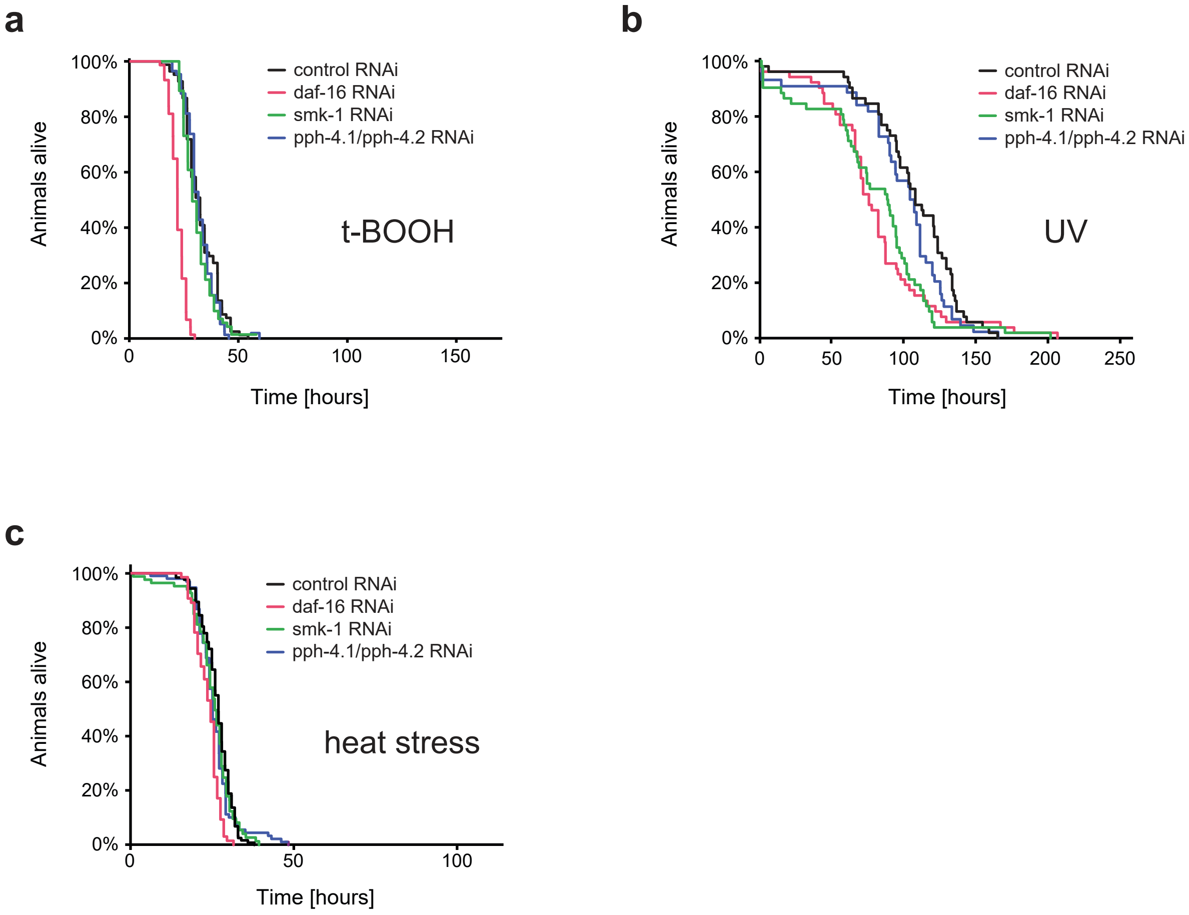

Supplementary Fig. 3 - In wild type animals, knockdown of PP4^{SMK-1} moderately impairs resistance to UV but not to oxidative stress or heat stress

Stress survival phenotypes caused by loss of *daf-16* or PP4^{SMK-1} in wild type animals. Animals were grown at 15°C from the L1 stage on the indicated RNAi bacteria. At the L4 stage the temperature was shifted to 25°C to fully inactivate *daf-2(e1370ts)*. On day 1 adulthood, animals were then transferred to 6 mM tBOOH containing RNAi plates (oxidative stress), exposed to 1500 J/m2 UV light (UV stress), or shifted to 32°C (heat stress). All strains used in in this figure harbored the *eri-1(mg366ts)* mutation to yield better knockdown efficiencies[1]. Loss of PP4^{SMK-1}, either by knockdown of *smk-1* or double-knockdown of *pph-4.1/pph-4.2*, impaired survival upon UV stress (b) but did not affect survival upon oxidative stress (a) or heat stress (c). For detailed statistics see Supplementary Table 4. Source data are provided as a Source Data file.

Sen et al., Supplementary Figure 4

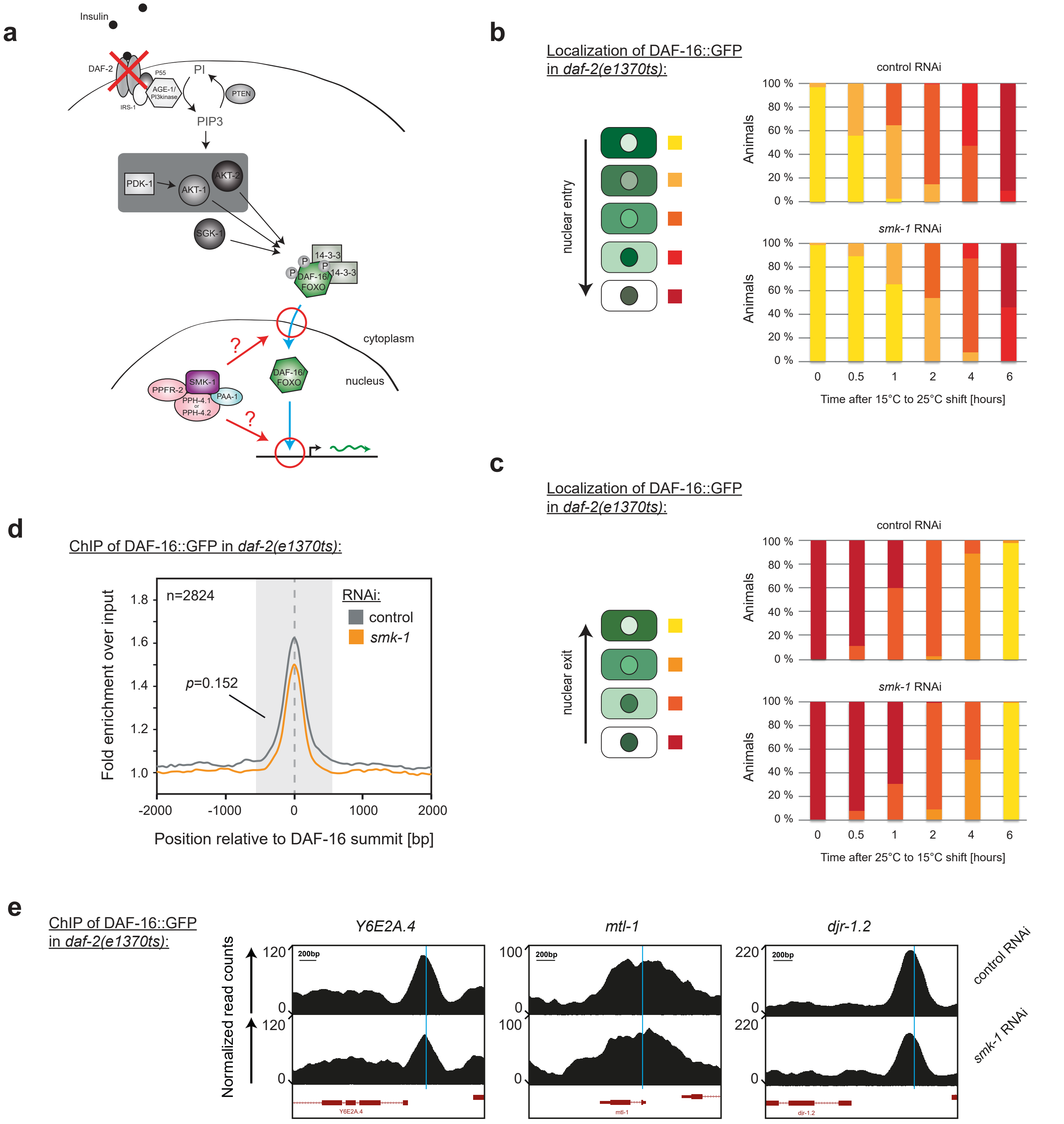

Supplementary Fig. 4 - PP4^{SMK-1} has only a mild effect on DAF-16 nuclear entry kinetics and does not affect DAF-16's binding to target promoters

a Schematic of the steps that lead from a low insulin/IGF signal to the activation of DAF-16, its nuclear entry, and eventual binding to its target promoters. **b, c** Kinetics of the nuclear entry (b) or nuclear exit (c) of DAF-16 upon inactivation or activation of the temperature-sensitive *daf-2(e1370ts)* allele, respectively. DAF-16::GFP expressing *daf-2(e1370ts)* mutant animals were synchronized and grown from the L1 stage on either control or *smk-1* RNAi bacteria. For nuclear entry (b), animals were shifted on day 2 of adulthood to 25°C and the localization of DAF-16::GFP was monitored in the intestine at the indicated timepoints. For nuclear exit (c), animals were shifted on day 1 of adulthood for 24h to 25°C, to achieve full nuclear entry of DAF-16::GFP, then shifted back to 15°C and the localization of DAF-16::GFP was monitored in the intestine at the indicated timepoints. For each timepoint, at least 100 animals were scored. See Supplementary Fig. 5 for representative images of animals in the different stages of nuclear translocation. **d** ChIP-seq analysis illustrating that there is no significant difference in DAF-16 binding to its target regions. *daf-2(e1370ts)* mutant animals expressing DAF-16::GFP were synchronized and grown from the L1 stage on either control or *smk-1* RNAi bacteria. At the L4 stage, animals were shifted to 25°C. After an additional 16 hours, animals were harvested and ChIP-seq analysis was conducted, using antibodies against GFP. Average read densities across the regions bound by DAF-16 under low IIS are shown. p-values indicate the significance of read density differences in the greyed region (-600 to +600 around the DAF-16 peak summits) between control and *smk-1* RNAi treated animals. **e** Examples of DAF-16 binding to different promoter regions in the UCSC genome browser. Source data underlying Supplementary Figures 4b-d are provided as a Source Data file.

Sen et al., Supplementary Figure 5

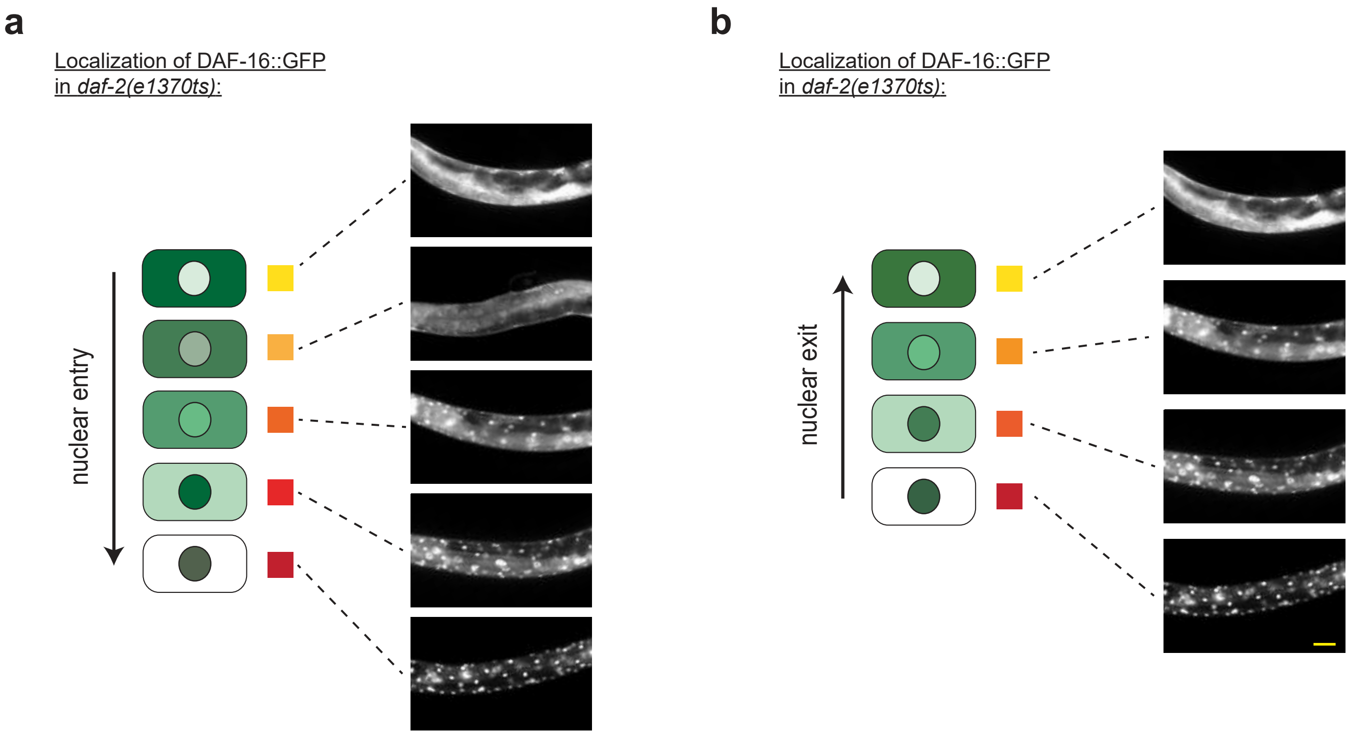

Supplementary Fig. 5 - Representative images of DAF-16::GFP in different stages of nuclear translocation

Representative images of *daf-2(e1370ts)* animals expressing DAF-16::GFP, resembling the different stages of nuclear entry and exit scored in Supplementary Figures 4b, c. (yellow scale bar: 50 µm)

Sen et al., Supplementary Figure 6

<u>ChIP of RNA Pol II in *daf-2(e1370ts)*:</u>

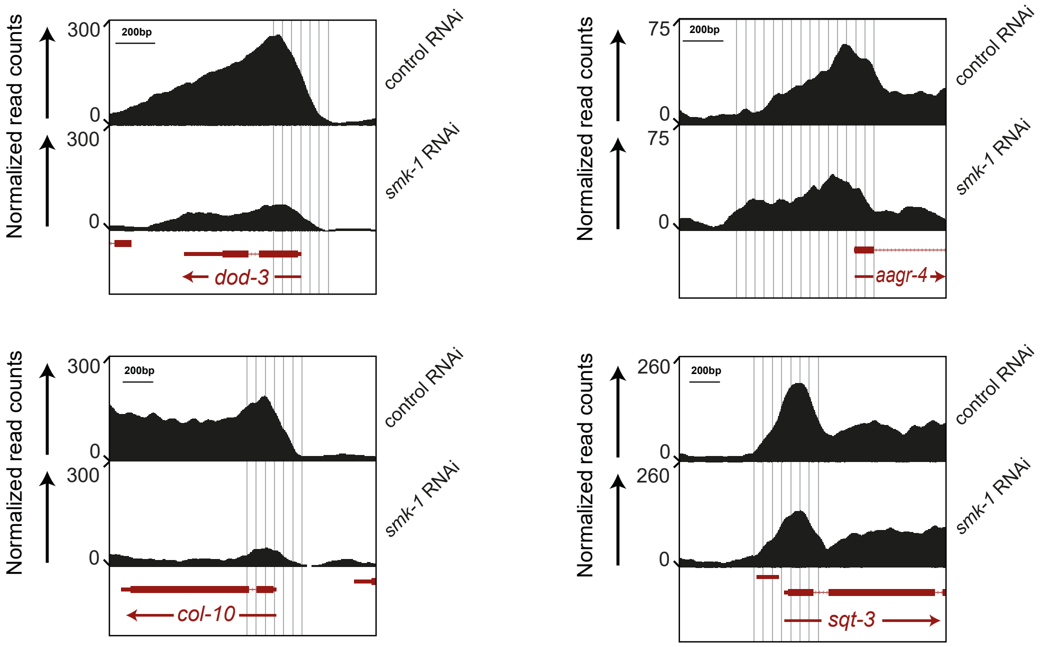

Supplementary Fig. 6 - Examples of RNA Pol II binding/distribution at individual genes in the presence or absence of PP4^{SMK-1}

Examples of RNA Pol II binding/distribution at individual TSS regions of genes co-activated by DAF-16 and SMK-1. Plots were obtained from the UCSC genome browser and are based on the ChIP-seq data used in Figure 5b.

Sen et al., Supplementary Figure 7

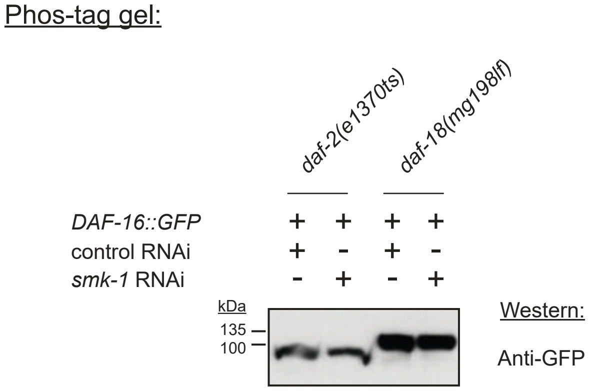

Supplementary Fig. 7 - Phos-tag SDS-PAGE shows no PP4^{SMK-1}-dependent change in the phosphorylation status of DAF-16

daf-2(e1370ts) (low IIS) and *daf-18(mg198lf)* (high IIS) mutant *C. elegans* were grown from the L1 stage on either control or *smk-1* RNAi bacteria. At the L4 stage, animals were shifted for 16 hours to 25°C, then harvested, lysed, and analyzed by phos-tag SDS-PAGE. DAF-16::GFP was detected using an anti-GFP antibody. Although under high IIS we can observe the expected upshift of DAF-16 due to phosphorylation of DAF-16 by AKT/SGK kinases, loss of PP4^{SMK-1} did not influence the migratory behavior of DAF-16 – neither under low nor high IIS.

Sen et al., Supplementary Figure 8

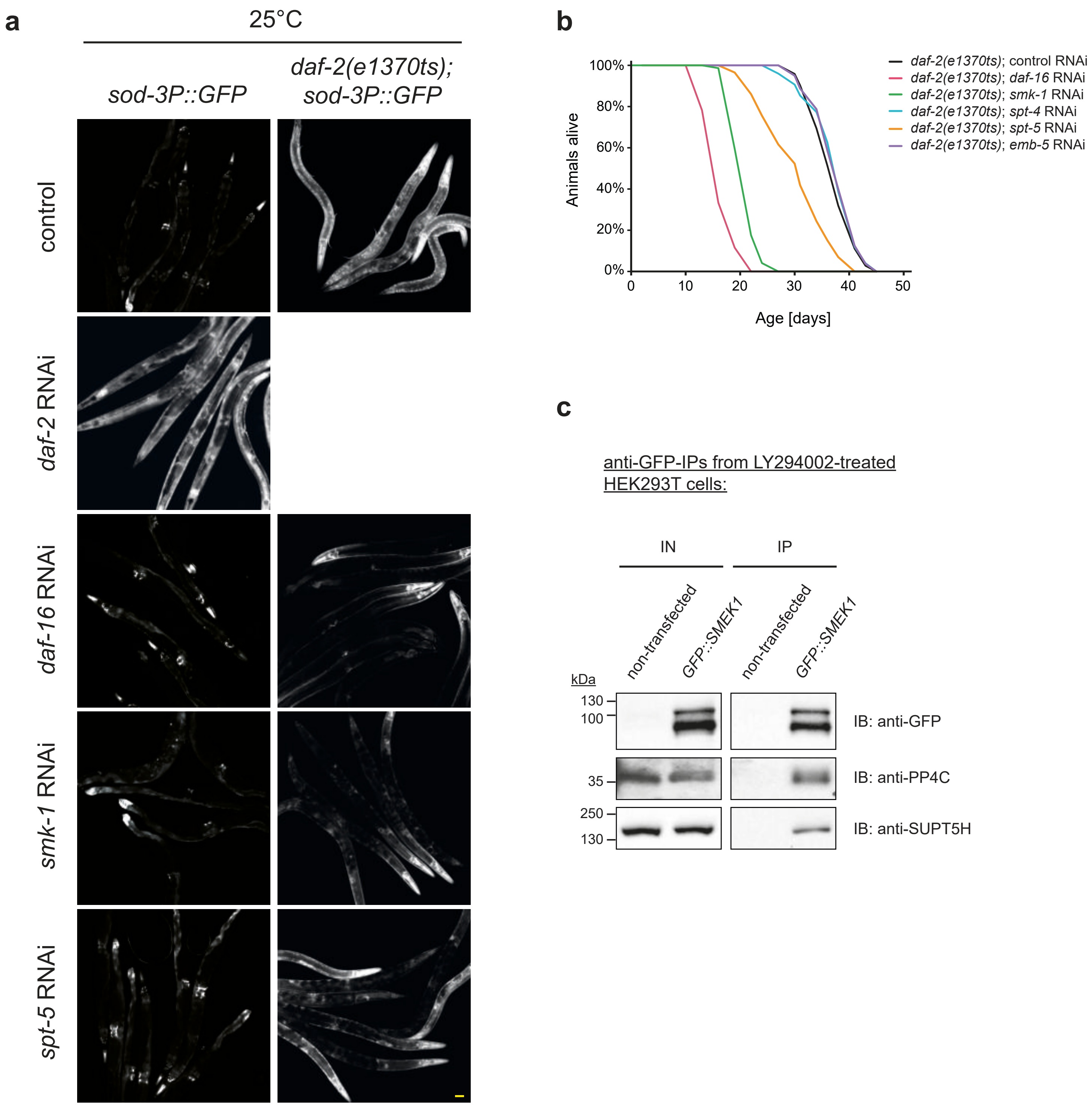

Supplementary Fig. 8 – Additional data related to SPT-5, SPT-4, and EMB-5

a Worm pictures show induction or suppression of *sod-3*, the canonical downstream target of DAF-16, upon treatment with control, *daf-2*, *daf-16*, *smk-1* or *spt-5* RNAi in wild type or with control, *daf-16*, *smk-1* or *spt-5* RNAi in *daf-2* mutant animals carrying *Psod-3::GFP*. Animals were synchronized by bleaching and were grown from the L4 stage on the indicated RNAi bacteria. They were kept at 25°C to fully inactivate *daf-2(e1370ts)* and FUDR (50 µM final concentration) was added to prevent production of progeny. (yellow scale bar: 50 µm) **b** Lifespan phenotypes caused by loss of the individual members of the SPT family of transcription elongation factors under low IIS. *daf-2(e1370ts); eri-1(mg366ts)* mutant animals were grown from the L4 stage on the indicated RNAi bacteria. Animals were kept at 25°C to fully inactivate *daf-2(e1370ts),* and FUDR (50 µM final concentration) was added to prevent production of progeny. The animal's survival was monitored. For detailed statistics see Supplementary Table 4. **c** Immuno-precipiation of the closest human ortholog to *C. elegans* SMK-1, SMEK1, form HEK293T cells under low IIS. HEK293T cells expressing SMEK1::GFP or non-transfected controls were treated with the PI3 kinase inhibitor LY-294002, lysed and the tagged proteins immunoprecipitated using GFP-Trap resin. Inputs (IN) and eluates (IP) of the IPs were analyzed by SDS-PAGE and western blotting, using antibodies against GFP, the only catalytic subunit of human PP4, PP4C, or the human ortholog of SPT-5, SUPT5H. For the inputs (IN), only fractions were loaded: 50% for the anti-GFP and anti-PP4C western blots and 4% for the anti-SUPT5H western blot. IB: antibody used for immunoblot. Source data underlying Supplementary Figure 8b is provided as a Source Data file.

Sen et al., Supplementary Figure 9

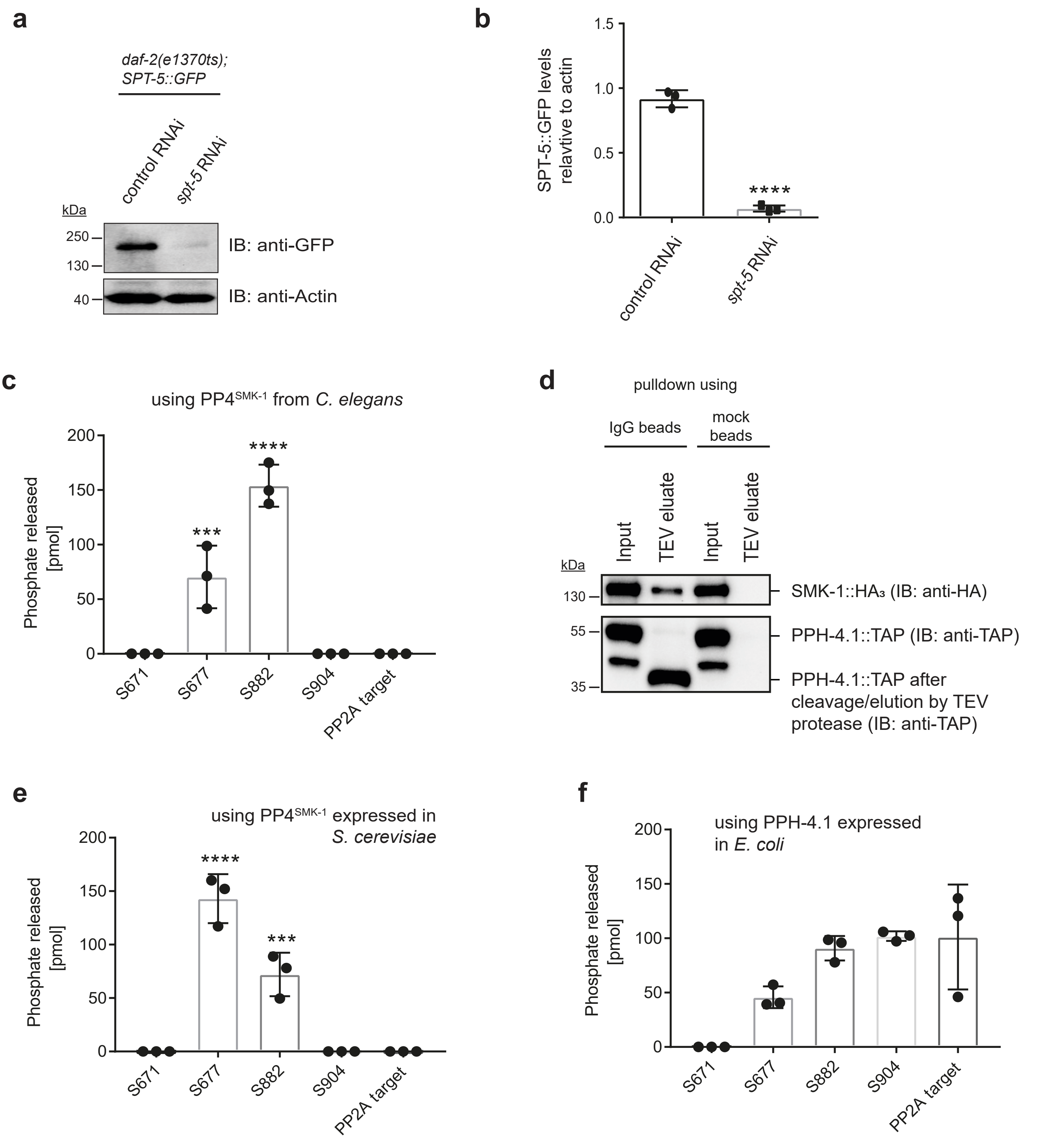

Supplementary Fig. 9 - SPT-5 is a direct target of PP4^{SMK-1}

a A representative western blot result, illustrating the knockdown efficiency of *spt-5* RNAi, as used in Figures 6 c-e. **b** Quantification of western blot results from 3 biological replicates as shown under (a) (****: p<0.0001, two-tailed T-test, error bars indicate s.d.). **c** *In vitro* phosphatase assay using purified PP4^{SMK-1} from *C. elegans*. Four different phosphopeptides containing the phosphosites S671, S677, S882, or S904 found on SPT-5 in our quantitative phosphoproteomics data were tested as substrates. A known PP2A target was used as negative control. Released phosphate amounts were determined by a Malachit assay and compared to the amounts released from the PP2A target (****: p<0.0001, ***: p<0.001, One-way ANOVA, error bars indicate s.d.). **d** The *C. elegans* PP4^{SMK-1} complex forms also when expressing its subunits recombinantly in *S. cerevisiae*. *S. cerevisiae* cells harboring Gal-inducible expression constructs for PPH-4.1::TAP, SMK-1::HA3 and PPFR-2 were grown to an OD_{600} of 1.0. Then Galactose was added and the cells harvested after 4 hours of induction. Lysates were generated and proteins purified using IgG beads or mock beads. Input and eluate samples were analyzed by SDS-PAGE and western blotting. **e, f** *In vitro* phosphatase assays identical to (c), but using either PP4^{SMK-1} recombinantly expressed and purified from *S. cerevisiae* (e) or PPH-4.1 recombinantly expressed and purified from *E. coli* (f) as the phosphatases. Source data underlying Supplementary Figures 9b, 9c and 9e-f are provided as a Source Data file.

Sen et al., Supplementary Figure 10

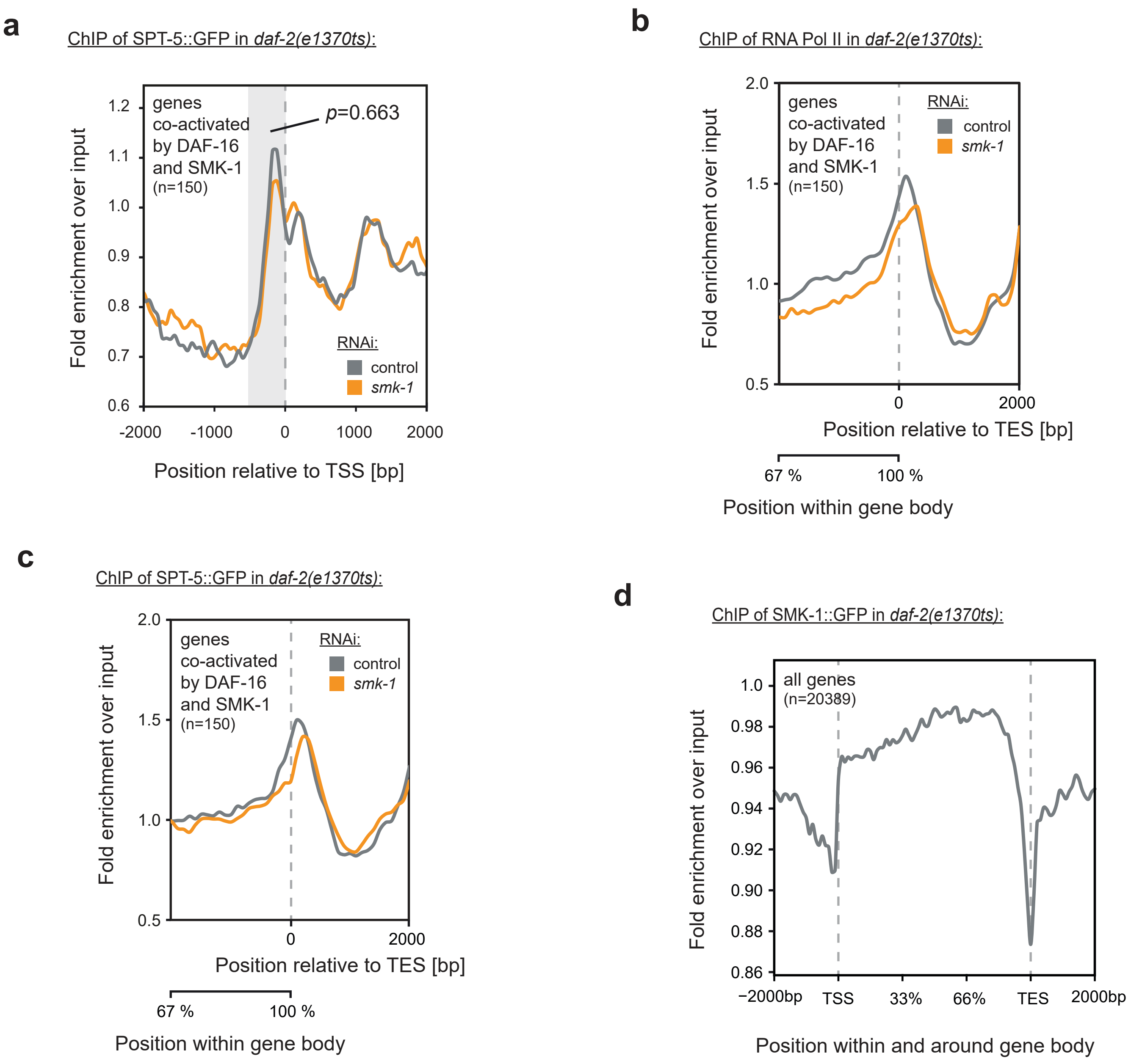

Supplementary Fig. 10 – Additional ChIP-seq analyses of SPT-5, Pol II, or SMK-1 binding to the genome

a ChIP-seq analysis of SPT-5 under low IIS shows that in the absence of PP4^{SMK-1} the binding of SPT-5 to promoter regions is not significantly affected – at genes co-activated by DAF-16 and SMK-1. *daf-2(e1370ts)* mutant animals expressing SPT-5::GFP were synchronized and grown from the L1 stage on either control or *smk-1* RNAi bacteria. At the L4 stage, animals were shifted to 25°C. After an additional 16 hours, animals were harvested and ChIP-seq analysis was conducted, using an antibody recognizing GFP. Average read densities across TSS regions of the indicated gene set are shown. The p-value indicates the significance of the read density difference in the greyed region (-480 to 0 bp before the TSSs) between control and *smk-1* RNAi treated animals. **b, c** ChIP-seq analyses of Pol II and SPT-5 under low IIS show that in the absence of PP4^{SMK-1}, the release of Pol II and SPT-5 at TES regions of genes co-activated by DAF-16 and SMK-1 may be mildly delayed. *daf-2(e1370ts)* mutant animals expressing SPT-5::GFP were synchronized and grown from the L1 stage on either control or *smk-1* RNAi bacteria. At the L4 stage, animals were shifted to 25°C. After an additional 16 hours, animals were harvested and ChIP-seq analysis was conducted, using antibodies recognizing either GFP or Pol II. Average read densities across TES regions of the indicated gene sets are shown. **d** SMK-1 may associate with gene bodies and possibly SPT-5 during transcriptional elongation but is released prior to transcriptional termination. ChIP-seq analyses. *daf-2(e1370ts)* mutant animals expressing SMK-1::GFP were grown asynchronously at 15°C. Then animals were shifted to 25°C for 16 hours, harvested, and ChIP-seq analysis was conducted, using antibody recognizing GFP. Average read densities across gene bodies of the indicated gene set are shown. Source data underlying Supplementary Figure 10a is provided as a Source Data file.

Sen et al., Supplementary Figure 11

Figure S7) Western Blot result of DAF-16 phosphorylation test on a Phos-tag gel

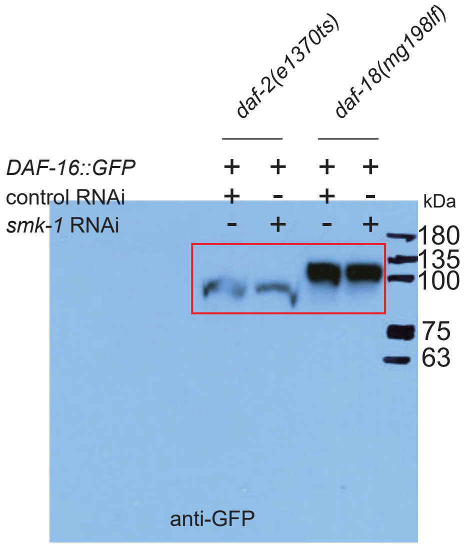

Figure S8 c) Western Blot results of PPP4C/SMEK1/SUPT5H Co-IP experiment

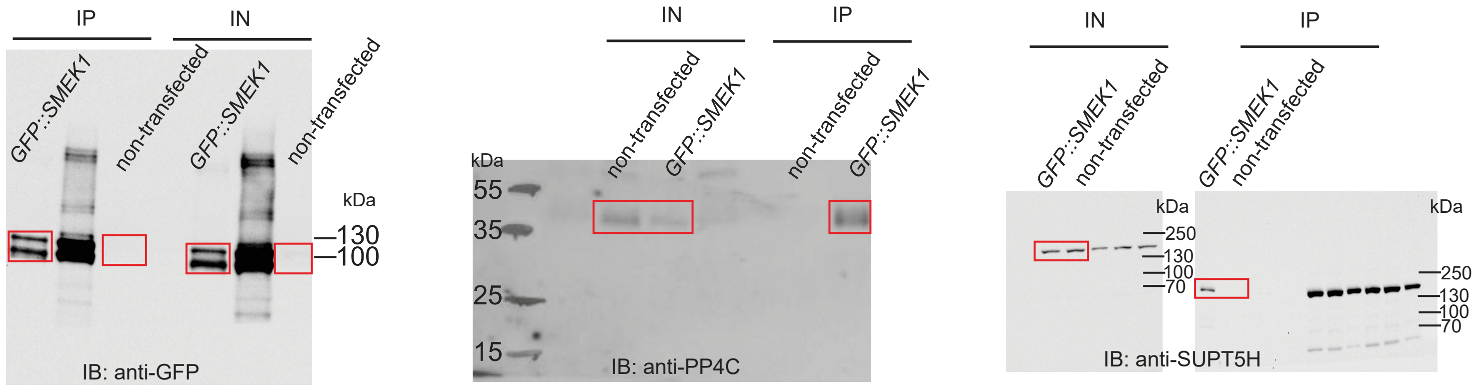

Figure S9 d) Western Blot result of PPH-4.1/SMK-1 Co-IP in yeast

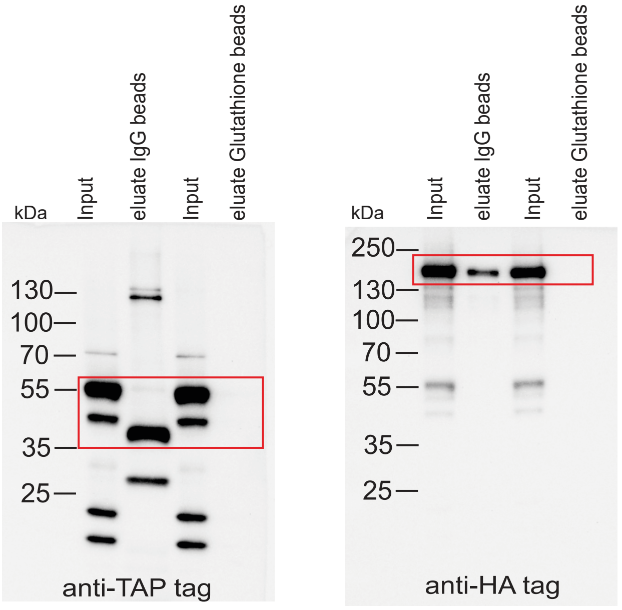

Figure S9 a) Western Blot result of *spt-5* knockdown

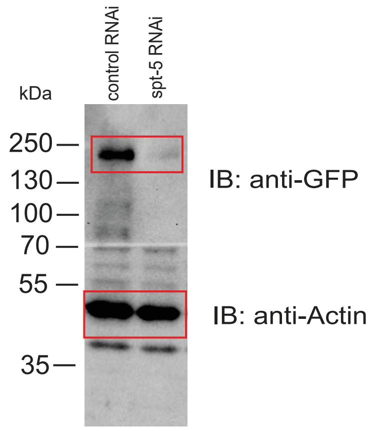

Supplementary Fig. 11 - Non-cropped Western Blot data

References:

1. Kennedy, S., Wang, D. & Ruvkun, G. A conserved siRNA-degrading RNase negatively regulates RNA interference in C. elegans. *Nature* **427**, 645–649 (2004).
2. Riedel, C. G. et al. DAF-16 employs the chromatin remodeller SWI/SNF to promote stress resistance and longevity. *Nat. Cell Biol.* **15**, 491–501 (2013).
3. Murphy, C. T. et al. Genes that act downstream of DAF-16 to influence the lifespan of *Caenorhabditis elegans*. *Nature* **424**, 277–283 (2003).
4. Ewald, C. Y., Landis, J. N., Porter Abate, J., Murphy, C. T. & Blackwell, T. K. Dauer-independent insulin/IGF-1-signalling implicates collagen remodelling in longevity. *Nature* **519**, 97–101 (2015).
5. Lin, X.-X. et al. DAF-16/FOXO and HLH-30/TFEB function as combinatorial transcription factors to promote stress resistance and longevity. *Nat. Commun.* **9**, 4400 (2018).
