# Supplementary material for: DAF-16/FOXO requires Protein Phosphatase 4 to initiate transcription of stress resistance and longevity promoting genes
Source: Nat Commun. 2020 Jan 9;11:138. doi: 10.1038/s41467-019-13931-7 (PMC6952425; doi:10.1038/s41467-019-13931-7)
Supplement: Supplementary file 3 — Reporting Summary [file 41467_2019_13931_MOESM3_ESM.pdf]

nature research

Corresponding author(s): Christian G. Riedel

Last updated by author(s): Nov 22, 2019

Reporting Summary

Nature Research wishes to improve the reproducibility of the work that we publish. This form provides structure for consistency and transparency in reporting. For further information on Nature Research policies, see Authors & Referees and the Editorial Policy Checklist.

Statistics

For all statistical analyses, confirm that the following items are present in the figure legend, table legend, main text, or Methods section.

n/a	Confirmed	
☐	☒	The exact sample size (*n*) for each experimental group/condition, given as a discrete number and unit of measurement
☐	☒	A statement on whether measurements were taken from distinct samples or whether the same sample was measured repeatedly
☐	☒	The statistical test(s) used AND whether they are one- or two-sided<br>*Only common tests should be described solely by name; describe more complex techniques in the Methods section.*
☒	☐	A description of all covariates tested
☒	☐	A description of any assumptions or corrections, such as tests of normality and adjustment for multiple comparisons
☐	☒	A full description of the statistical parameters including central tendency (e.g. means) or other basic estimates (e.g. regression coefficient) AND variation (e.g. standard deviation) or associated estimates of uncertainty (e.g. confidence intervals)
☒	☐	For null hypothesis testing, the test statistic (e.g. *F*, *t*, *r*) with confidence intervals, effect sizes, degrees of freedom and *P* value noted<br>*Give P values as exact values whenever suitable.*
☒	☐	For Bayesian analysis, information on the choice of priors and Markov chain Monte Carlo settings
☒	☐	For hierarchical and complex designs, identification of the appropriate level for tests and full reporting of outcomes
☒	☐	Estimates of effect sizes (e.g. Cohen's *d*, Pearson's *r*), indicating how they were calculated

Our web collection on statistics for biologists contains articles on many of the points above.

Software and code

Policy information about availability of computer code

Data collection	Acquisition and analysis software of the "lifespan scoring machine" (Stroustrup et al., Nature Methods 2013)
Data analysis	Online application for survival analysis (OASIS 2)<br>DAVID Bioinformatics Resources versions 6.7<br>IBM SPSS Statistics software versions 23 and 24<br>GraphPad Prism 7.0<br>Excel<br>Fiji, ImageJ 1.51s<br>MaxQuant<br>R

For manuscripts utilizing custom algorithms or software that are central to the research but not yet described in published literature, software must be made available to editors/reviewers. We strongly encourage code deposition in a community repository (e.g. GitHub). See the Nature Research guidelines for submitting code & software for further information.

Data

Policy information about availability of data

All manuscripts must include a data availability statement. This statement should provide the following information, where applicable:

- Accession codes, unique identifiers, or web links for publicly available datasets
- A list of figures that have associated raw data
- A description of any restrictions on data availability

Data availability:
The high-throughput sequencing data generated and analyzed during this study are available from the authors upon reasonable request as well as from the Sequence Read Archive at NCBI at the following accession code: PRJNA560378. The mass spectrometry data generated and analyzed during this study are available

from the authors upon reasonable request as well as from the PeptideAtlas at the following accession code: PASS01428.
The source data underlying Figures 2a-f, 3a-f, 5b, d, 6c-e and Supplementary Figures 2a-f, 3a-c, 4b-d, 8b, 9b, c, e, f and 10a are provided as a Source Data file. Non-cropped western blot data underlying Supplementary Figures 7, 8c and 9a, d are provided in Supplementary Figure 11.

Field-specific reporting

Please select the one below that is the best fit for your research. If you are not sure, read the appropriate sections before making your selection.

☒ Life sciences ☐ Behavioural & social sciences ☐ Ecological, evolutionary & environmental sciences

For a reference copy of the document with all sections, see nature.com/documents/nr-reporting-summary-flat.pdf

Life sciences study design

All studies must disclose on these points even when the disclosure is negative.

Sample size	Sample sizes for each experiment were chosen in accordance with the general standards and prior publications in the respecitve fields.
Data exclusions	There are no data exclusions.
Replication	All the data shown in the manuscript were reproduced at least once.
Randomization	For all experiments, animals were randomly allocated to the experimental groups.
Blinding	During data collection, researchers were blinded to group allocation.

Reporting for specific materials, systems and methods

We require information from authors about some types of materials, experimental systems and methods used in many studies. Here, indicate whether each material, system or method listed is relevant to your study. If you are not sure if a list item applies to your research, read the appropriate section before selecting a response.

Materials & experimental systems

n/a	Involved in the study	
☐	☒	Antibodies
☐	☒	Eukaryotic cell lines
☒	☐	Palaeontology
☐	☒	Animals and other organisms
☒	☐	Human research participants
☒	☐	Clinical data

Methods

n/a	Involved in the study	
☐	☒	ChIP-seq
☒	☐	Flow cytometry
☒	☐	MRI-based neuroimaging

Antibodies

Antibodies used

Takara Living Colors® Full-Length GFP Polyclonal Antibody, Cat# 632592 Lot# 1510024
Abcam Anti-RNA polymerase II CTD repeat YSPTSPS (phospho S5) antibody [4H8] - ChIP Grade (Cat# ab5408), Lot#GR205997-2
BioLegend Anti-RNA Polymerase II Antibody [8WG16]; Monoclonal, Cat# 920102, Lot# B200433
Roche anti-GFP antibody, monoclonal, Cat # 11814460001
Abcam Anti-PPP4C antibody, Polyclonal, Cat# Ab70623, Lot# GR258985-4
Abcam Anti-SPT5 antibody, Polyclonal, Cat# ab26259, Lot# GR3193762-1
Abcam Anti-HA antibody, Polyclonal, Cat#ab9110 Lot#31995553-3
Antibodies online.com, Anti-TAP antibody, Polyclonal, Cat#ABIN398491
Milipore Anti-Actin monoclonal (clone C4) , Cat#MAB1501 Lot#3018859

Validation

All the validations and citations can be found on the manufacturers' websites.

Eukaryotic cell lines

Policy information about cell lines

Cell line source(s)

HEK293T cells from ATCC.

Authentication	Authentication was done by ATCC.
Mycoplasma contamination	All cell lines were tested to be negative for mycoplasma contamination.
Commonly misidentified lines (See ICLAC register)	*Name any commonly misidentified cell lines used in the study and provide a rationale for their use.*

Animals and other organisms

Policy information about studies involving animals; ARRIVE guidelines recommended for reporting animal research

Laboratory animals	The nematode Caenorhabditis elegans was used. All the strains used are described in Supplementary Table 1. Animals were hermaphrodites.
Wild animals	No wild animals were used in this study.
Field-collected samples	This study did not involve samples collected from the field.
Ethics oversight	In Sweden, no ethical approval is needed for experiments using C. elegans or HEK293T cells.

Note that full information on the approval of the study protocol must also be provided in the manuscript.

ChIP-seq

Data deposition

☒ Confirm that both raw and final processed data have been deposited in a public database such as GEO.

☒ Confirm that you have deposited or provided access to graph files (e.g. BED files) for the called peaks.

Data access links *May remain private before publication.*	The high-throughput sequencing data generated and analyzed during this study are available from the Sequence Read Archive at NCBI at the following accession code: PRJNA560378.<br>The mass spectrometry data generated and analyzed during this study are available from the PeptideAtlas at the following accession code: PASS01428.
Files in database submission	We provide demultiplexed FASTQ files and BigWig files. All files are clearly labelled/annotated. Furthermore, a BED file of DAF-16 peaks that has been used in Supplementary Figure 4d is provided.
Genome browser session (e.g. UCSC)	At the above data access link, we also provide BigWig (.bw) files. We suggest to download these and view them offline by using the software IGV.

Methodology

Replicates	Results were confirmed at least once in independent experiments.
Sequencing depth	All sequencing was single-ended to a length of 50 bases. Each sample was sequenced to a depth of 7.8 to 34 Million mapped reads.
Antibodies	Takara Living Colors® Full-Length GFP Polyclonal Antibody, Cat# 632592, Lot# 1510024<br>Abcam Anti-RNA polymerase II CTD repeat YSPTSPS (phospho S5) antibody [4H8] - ChIP Grade (Cat# ab5408), Lot#GR205997-2<br>BioLegend Anti-RNA Polymerase II Antibody [8WG16]; Monoclonal, Cat# 920102, Lot# B200433
Peak calling parameters	Peak calling was not conducted as part of this study. The peaks used in Supplementary Figure 4 were taken from the literature.
Data quality	As stated in the previous point, no peak calling was conducted as part of this study.
Software	We used FastQC, Bowtie 2, MACS 2, ngs.plot, and DeepTools.
