# Supplementary material for: DAF-16/FOXO requires Protein Phosphatase 4 to initiate transcription of stress resistance and longevity promoting genes
Source: Nat Commun. 2020 Jan 9;11:138. doi: 10.1038/s41467-019-13931-7 (PMC6952425; doi:10.1038/s41467-019-13931-7)
Supplement: Supplementary file 2 — Peer Review [file 41467_2019_13931_MOESM2_ESM.pdf]

Reviewers' comments:

Reviewer #1 (Remarks to the Author):

In this manuscript entitled “DAF-16/FOXO requires Protein Phosphatase 4 to initiate the transcription of stress resistance and longevity promoting genes”, Sen et al., set to identify how SMK-1 regulates low insulin/IGF signaling (IIS). They use C. elegans and identify that SMK-1 is a component of a specific PP4 complex isoform, and this complex is essential for DAF-16-driven gene expression. Additionally, the authors show that loss of PP4(SMK-1) impairs PolII recruitment and transcriptional initiation & termination. Furthermore, they propose that SPT-5, a conserved transcriptional regulator, is a probable substrate for SMK-1. In sum, this is very interesting and, in most areas, extremely well executed work. However, the mass spectrometry part is not adequately described and, in many parts, contain contains possible mistakes, requiring revision. Also illustrating that SPT-5 is directly substrate for PP4(SMK-1) and if the dephosphorylation all the four sites (671, 677, 882 and 904) is needed for the effect.

Major quibbles

- The mass spectrometry parameters, especially the database search parameters are not adequately described, as well as the tables contain very little actual information and do not fulfill the minimum information needed to be reported with this type of analyses.

More specifically:

o What does the authors mean with large-scale IP?

o Who many replicate analyses were used in Fig.1b? And how were the data specific interactors filtered using the untagged control?

o Figure 1b, with how many unique peptides the proteins were identified?

o Supplementary Table 2 and 3 state that “Only proteins identified by at least five unique peptides are shown.”. Is this correct or are the authors mixing up “unique peptides” and “spectral counts”. These are not the same thing.

o The Supplementary Table 4 contains again very little information. Are all the four STP-5 phosphosites (671, 677, 882 and 904) detected from different peptides or from the same? The peptide sequences and site probabilities for the modification would be needed.

o Additionally Supplementary Table 4 (Proteins containing phosphorylations that were seen only in the absence of smk-1) has mistakes in the last column (Phos site within isoforms).

Minor quibbles

- To convincingly illustrate their proposed mechanism(s), the authors that should show which of the SPT-5 phosphosites are direct targets for PP4(SMK-1) and which of these are needed for SPT-5 activity.

Reviewer #2 (Remarks to the Author):

This manuscript explores the mechanism by which SMK-1 modulates output of the transcription factor

DAF-16 to promote longevity and confer stress resistance in C. elegans. It has long been recognized that DAF-16 is a key effector of the increased lifespan and enhanced stress tolerance of animals maintained under conditions of low insulin/IGF signaling (IIS). SMK-1 was identified as a potent upstream regulator of this signaling, but the underlying mechanism was unknown. Here the authors perform mass spectrometry with a tagged version of SMK-1, and identify subunits of the protein phosphatase 4 (PP4) complex as interaction partners. By genetic and RNAi-based manipulations, they make the case that PP4 and SMK-1 are in a common pathway, upstream of DAF-16, which regulates lifespan under low IIS conditions (experimentally induced by a mutation in DAF-2, the IGF receptor). Moreover, as is the case with SMK-1, RNAi targeting PP4 partially ablates DAF-16-dependent resistance to oxidative stress and UV, but has no effect on the response to heat stress, consistent with a specific role of both SMK-1 and PP4 upstream of DAF-16. To identify potential downstream targets of SMK-1/PP4, the authors perform a phosphoproteomic analysis after SMK-4 knockdown, and identify the conserved transcription elongation factor SPT-5 as a putative PP4 target; SPT-5-derived phosphopeptides are enriched in SMK-1-depleted animals, relative to non-targeted controls. Supporting SPT-5's participation in a DAF-2-PP4-DAF-16 pathway, RNAi targeting SPT-5 had similar, selective effects on lifespan and both oxidative and genotoxic stress responses. SMK-1 knockdown, moreover, had effects on RNA polymerase II chromatin distribution (measured by ChIP-seq) that are at least broadly consistent with derangement of known functions of SPT-5 in pausing and elongation. This is a novel and in many ways attractive mechanism, and the genetic data suggesting functional links among these players are mostly convincing, but there are significant gaps and omissions, and some dubious interpretations, which would need to be corrected before I could recommend publication. These might best be addressed, in most cases, by softening or removing some of the mechanistic conclusions and discussing alternative interpretations. In at least one case (point 1), however, additional experimental support will be needed.

My specific concerns are:

1. The purported association of SMK-1 with PP4 catalytic and regulatory subunits in C. elegans is based almost entirely on the mass spectrometry (Fig. 1a, b); the only independent "validation" of this interaction is done in human HEK293 cells overexpressing a GFP-tagged version of the human ortholog SMEK1 (Fig. S5). The model in Fig. 1c posits SMK-1 as an intrinsic component of a functional PP4 complex, so it is critical to have some additional support for this idea. Can this complex be reconstituted from recombinant subunits? What is the stoichiometry of the interaction? How does SMK-1 association affect PP4 activity? I realize these are not trivial issues to address in C. elegans, where (I presume) antibodies to the relevant proteins are not widely available, but some additional characterization of the putative PPH-4/PPFR-2/SMK-1/PAA-1 complex is needed. Do these proteins co-purify (e.g. by size exclusion chromatography or sedimentation)? If no further biochemical analysis is feasible, can the genetic argument for their concerted function be strengthened (i.e., by testing for epistasis between SMK-1 and PPH-4.1/PPH-4.2 RNAi)?

2. Of the four sites within SPT-5 that become hyperphosphorylated upon SMK-1 knockdown, two are within the conserved P-TEFb target region (CTR/CTD), but neither is a canonical, "proline-directed" (S/T-P) site, of which there are many (mostly T-P) within the C. elegans protein. This is important context for trying to explain the effects of SMK-1 versus SPT-5 depletion, which the authors don't acknowledge. In my opinion there is a strong likelihood that the phosphorylation events apparently being reversed by PP4/SMK-1 are playing a different role from that of the classical CTR/CTD phosphorylations described in previous papers. (Presumably, SPT-5 is still being phosphorylated on all those other sites.)

3. I found the interpretation of SMK-1 RNAi effects on Pol II distribution (Fig. 5b-d) to be generally unpersuasive. The authors suggest the biphasic distribution of ChIP-seq signals around the TSS of DAF-16-/SMK-1-dependent genes is evidence of Pol II in two different stages of transcription: paused and pre-initiated. Is the latter analogous to the "docked" Pol II described in ref. 19? The effect of SMK-1 RNAi on this pattern is a general reduction in Pol II cross-linking, with maybe some skewing

towards more Pol II upstream of the TSS, which the authors interpret as an effect on initiation. Whether or not Pol II has initiated cannot be deduced from ChIP-seq analysis alone, nor is Ser5 phosphorylation of the Pol II CTD (5d) a reliable marker of initiation. Other interpretations need to be considered as the basis for this distribution, e.g. divergent transcription.

4. Similarly, the authors argue that SMK-1 RNAi leads to impaired termination, based on a very modest shift of the Pol II and SPT-5::GFP ChIP-seq peaks downstream of the "TES" (which I presume is actually the polyadenylation signal, i.e., not where Pol II is actually terminating). This is another case of trying to fit the data into a pre-existing model, which was based on measurement of actively transcribing Pol II, as opposed to simple occupancy (ref. 33). I note that SMK-1 RNAi is having little or no effect on the position where Pol II or SPT-5 ChIP signals are returning to baseline downstream of the "TES," suggesting there might be little effect on termination per se.

5. There is an apparent (slightly counterintuitive) loss of SPT-5 recovery in SMK-1 IPs from low IIS (Supp. Table 2) relative to high IIS mutants (Supp. Table 3). Do the authors believe this to be significant? If so, could they say something about it? (If not, explain why it is not.)

6. Why is the effect of the ppfr-4 RNAi on lifespan of the daf-2 daf-16 mutant biphasic (Supp. Fig. 1b)? (This is a minor point.)

7. There are a number of places in the manuscript where figure call-outs are omitted, making it harder to follow, for example, in line 129 (should be Fig. 2c, I think).

8. The abbreviation "t-BOOH" first appears in line 154 but is not defined here (or anywhere).

9. Although I am skeptical about the mechanistic interpretations of Pol II distributions in Fig. 5 (see point 3), the difference between SMK-1-dependent and –independent, DAF-16-dependent genes is very interesting. It should be quantified, e.g. by comparing the pausing index (PI) of these two classes (to each other and to all genes). Is SMK-1-dependence significantly correlated with low PI?

10. To assess whether putative substrates of PP4 are contributing to its regulation of DAF-16-dependent gene expression, the authors use a reporter assay, results of which are presented in semi-quantitative form in Fig. 6b ("+" vs. "++" vs. "blank"). I would recommend showing the actual data, at least for "positive" targets.

11. As the authors point out in the discussion, SPT-5 is an ancient, highly conserved and universally essential elongation factor engaged at most or all Pol II genes, yet its knockdown "had no effect in wild-type or daf-16 null mutants" (line 300, Fig. 6c-f). A likely explanation is residual SPT-5 protein; is there any way to assess the efficiency of protein (or, failing that, mRNA) depletion?

12. Can the authors cite a reference for the statement that "SPT-5's CTD is unphosphorylated…until it has traveled with Pol II to the pause site" (lines 319-20)? This seems plausible by analogy with the Pol II CTD, but I am not aware of any data to that effect.

13. There are other, similar instances in which the authors' mechanistic statements and inferences go well beyond what has been shown in the literature or by their data, for example, saying that SPT-5 CTD dephosphorylation is needed for Pol II and DSIF recycling and reinitiation (lines 325-7), that SPT-5 phosphorylation needs to be low during promoter clearance (lines 332-3), and so on. The last two paragraphs of Results (lines 328-66) can (and probably should) be re-written to say, simply, that "perturbations of SMK-1 alter Pol II and SPT-5 distribution on DAF-16/SMK-1-responsive genes, possibly through SMK-1 association with PP4, in a manner broadly consistent with known roles of SPT-5 in transcription elongation and termination."

14. In the Discussion, the authors speculate (their word) that the proposed mode of DAF-16/SMK-1

gene regulation at the initiation step might allow rapid induction in response to stimuli (lines 418-9). It is worth pointing out that the exact same rationale has been proposed for why certain genes are pause-regulated. I suspect that neither generalization is entirely accurate, so this is probably best left unsaid at this point.

Reviewer #3 (Remarks to the Author):

In their manuscript entitled "DAF-16/FOXO requires Protein Phosphatase 4 to initiate the transcription of stress resistance and longevity promoting genes", Sen et al. propose a model that explains how a long-known regulator of lifespan and of the longevity-promoting transcription factor DAF-16, SMK-1, actually exerts its effects on DAF-16 target gene expression and thus, longevity in the nematode C. elegans. Using a combination of mostly high throughput biochemical/proteomics and molecular biological/genomics-transcriptomics approaches, as well as genetic approaches in C. elegans, the authors (1) provide evidence for SMK-1 indeed being a subunit of PP4-complex(es); (2) show that PP4-subunits are required for full longevity of long-lived C. elegans daf-2 mutant/RNAi worms and that they act in a common pathway with daf-16; (3) define the stress resistance pattern of PP4/SMK-1 compromised worms; (4) provide evidence for PP4/SMK-1 being required for efficient RNA Pol II promoter recruitment of DAF-16/SMK-1 coregulated genes and (5) provide evidence for SPT-5 being the critical target of PP4/SMK-1 whose dephosphorylation is important for proper transcription at a subset of DAF-16 target genes. These findings are novel, of interest to experts in the field of C. elegans aging research, and may have implications for other organisms as the molecular regulators under investigation are conserved in humans and the authors even provide evidence for physical interaction between the human orthologs of SMK-1 and SPT-5. The wealth of experiments conducted collectively supports all conclusions drawn, although suboptimal controls were used in some cases and low-throughput validation of high throughput proteomics/RNAseq-data is not provided. Moreover, the methods section lacks important experimental information that would be of interest to experts in the field. Thus, the manuscript requires some revision to warrant publication in Nature Communications.

Specific issues:

Introduction
Line 47: "…where it [DAF-16] eventually binds to the promoters of many stress resistance and longevity promoting genes and drives their transcription". Misleading. DAF-16 appears to also repress the expression of certain lifespan-limiting genes (Murphy et al., Nature volume 424, pages 277–283 (17 July 2003)) → rephrase
Section SMK-1 is part of a nuclear Protein Phosphatase 4 complex (PP4SMK-1)
Interaction of SMK-1 with PP4-subunits was detected by large-scale IP/mass spec and confirmed by another large-scale IP/mass spec experiment using a different tag on SMK-1. A low throughput IP/Western Blot experiment would be more standard as a confirmation. Why was this not done? In principle, the authors have SMK-1 and PPH4-4.1 with different affinity tags.
Line 85: "At the same time, given that SMK-1 is required for DAF-16 functions specifically when IIS activity is low,…" Misleading: Wolff et al. (ref. 6 cited by the authors) also show that smk-1 is required for the longevity of germline-ablated animals. Even though germline-longevity is also dependent on daf-16 (Hsin and Kenyon, 1999), several lines of evidence (esp. Lin et al., 2001, Berman and Kenyon 2006) suggest that regulation and/or function of daf-16 by germline signals differs from that in/by reduced IIS → rephrase
Line 94-96: The detection of PAA-1 in SMK-1 IPs is not necessarily a hint towards PAA-1 being part of a PP4-complex. Could be the other way round, namely SMK-1 being part of a PP2A-complex. Generally, from IP/MS-experiments, the authors cannot conclude which co-precipitating proteins are part of which out of potentially several SMK-1 complexes → phrase more carefully

Fig. 1a. Labeling a worm strain not expressing the SMK-1::mCherry transgene as "untagged" is

misleading as it in the first place suggests that it expresses a transgene containing plain SMK-1. Simply call it "control". The more appropriate IP-control would have been a strain expressing mCherry (without the SMK-1 ORF) from the same promoter. Thus, it is absolutely necessary that the authors validate their IP by another approach. Unfortunately, the chosen one (different tags but also IP/MS) lacks the information of what the "control purification" was in this case. As indicated above, a low throughput IP/Western Blot experiment would be desirable.

Fig. 1d: only 1 worm shown, i.e. it is not possible to estimate how robust the localization pattern is across the population ↳ would be great if images with multiple worms were provided; moreover, IIS is known to regulate longevity during adulthood (Dillin et al, Science Vol. 298, Issue 5594, pp. 830-834) ↳ images from day 1 adults would be more informative than L4 larvae.

Supplementary table 2/3: As the results are presented in different tables, it is difficult to compare the lists and see that the spectral count numbers are very similar for the highlighted proteins ↳ combine in one table.

Section PP4SMK-1 is required for DAF-16 to promote longevity
Line 132: "Next, we validated the phenotypes obtained from knockdown of the PP4SMK-1 catalytic subunits by use of a null mutant allele of pph-4.1." What is the evidence for the allele used being a null (i.e. complete loss of function)?
Line 139: "Consistent with the catalytic activity of PP4SMK-1 being required, overexpression of the catalytic dead form of PPH-4.1 led to lifespan shortening in daf-2 mutant animals". Phrase more carefully in the light of Fig. 2F, which shows that daf-2(-) worms overexpressing the R262L-mutant are still substantially long-lived (by inference; a wt control was not included in this experiment), although not as long-lived as daf-2(-) worms overexpressing wildtype PPH-4.1. This would mean that catalytic activity contributes, but is not absolutely required for daf-2 longevity, although it could be a dose effect as both overexpression lines still harbor endogenous pph-4.1 activity. ↳ To address this possibility, include daf-2(e1370) animals not overexpressing any of the two constructs! Generally, to better assess the requirement of PPH-4.1 catalytic activity, expression of R262L and wt PPH-4.1 needs to be done in the pph-4.1 mutant background!

Fig. 2 and supplementary table 5: It seems as if the data in the tables (and by extension, the graphs) show pooled results from multiple experiments (Figure legend says n>100, but table contains e.g. n = 529, which would be rather unusual for a single experiment). This needs to be clarified. If the data is pooled, from how many trials? Why are worm numbers vastly different in some cases (e.g. 2A: n = 175-529)? What about censoring? In many tables, there are n < 100, contrary to what the figure legend suggests.

Fig. 2C: smk-1 RNAi should have been included in these particular experiments, to be really able to compare the combined knockdown of the two catalytic subunits to smk-1

Fig. 2F: How many independent transgenic lines were examined? Generally, the field requires at least two independent lines.
Section PP4SMK-1 is required for DAF-16 to promote resistance to oxidative stress and UV irradiation but not to heat stress
Line 147: "Much of the DAF-16-driven longevity in daf-2 mutant animals can be attributed to improved stress responses". Include a reference for this statement.

Fig. 3 and supplementary table 5. Similar to Fig.2. In some tables it seems as if only one single experiment was conducted (e.g. Fig. 3D, n ~ 50!). Clarify whether this is one single experiment/pooled data from multiple experiments (n?)/representative experiment (n?). Again, why is n in some cases so different in some panels?

Section PP4SMK-1 is required for a significant part of the DAF-16-mediated gene expression changes

in low IIS mutants
Line 172: "Using the resulting data, we first determined the genes differentially expressed by the reduced IIS in daf-2 mutant animals, identifying 1093 upregulated and 2054 downregulated genes (Fig. 4a)." How well do the authors' lists of up/downregulated genes overlap with published RNAseq/microarray studies comparing wt and daf-2 animals or daf-2 and daf-2; daf-16 animals? Comment on this, to validate your sequencing data. Also, how is up/downregulation defined? Specify here and/or in Methods section! What about targets of other transcription factors involved in daf-2 longevity (e.g. skn-1)?
Fig. 4. D, e: The GO-term "Aging" is very superficial. The authors extensively described the stress resistance phenotypes of PP4-ko…look specifically for co-regulation of genes that could explain this phenotype as well!

Section PP4SMK-1 only mildly affects nuclear entry of DAF-16 and its binding to chromatin
Fig. S3: Not clear how many animals were scored per strain and how many experiments were conducted. Provide representative images of the 5 different states distinguished during scoring.

Section PP4SMK-1 promotes DAF-16-driven longevity and stress resistance by dephosphorylating SPT-5
Fig. 6b: Provide images of SPT-5 influencing expression of the sod-3 reporter

Section PP4SMK-1 promotes DAF-16-driven longevity and stress resistance by dephosphorylating SPT-5
Line 301: "Overall, our data is consistent with SPT-5 being the relevant substrate, by which PP4SMK-1 promotes Pol II recruitment and transcriptional initiation at many DAF-16 target genes and thus fulfills its lifespan and stress resistance regulatory roles under low IIS" Correct, the data are consistent with this conclusion. To further substantiate this conclusion that PP4SMK-1 promotes longevity by dephosphorylating SPT-5, the authors need to show that expression of a constitutively dephosphorylated SPT-5 can overcome the lifespan-shortening effect of pp4-1/2 RNAi in daf-2 mutant animals. Moreover, the data do not provide convincing evidence for SPT-5 being a direct PP4SMK-1 substrate. This requires an in vitro phosphatase assay.
Dephosphorylation of SPT-5 by PP4SMK-1 promotes multiple transcriptional events, including Pol II recruitment, transcriptional initiation, and transcriptional termination
Cf. comment above. The text (here and also in the discussion) as it is written implies that SPT-5 is a direct substrate. Perform an in vitro phosphatase assay!

Discussion

Line 376: "This complex contains only some of the possible PP4 subunits, namely PPH-4.1/PPH-4.2, providing the catalytic activity, as well as PPFR-2 and SMK-1, two regulatory subunits. Notably, we found also PAA-1 in this complex, normally a structural subunit of PP2A complexes11. It will be interesting to explore in follow-up studies, why this subunit is present in PP4SMK-1." This is all based on IP/mass spec studies, which, as indicated above, cannot distinguish whether proteins interacting with a bait protein do so as part of a common complex or of separate complexes. Answering this question would require reciprocal co-IP studies on all proteins in question. Perform these experiments or phrase more carefully!

Materials and Methods

This section lacks many important details that may unnecessarily complicate potential attempts to reproduce the data by other labs
- The authors use strains from several labs. Where alleles obtained from other labs (CB, KW, AS, CF-strains) outcrossed to the author lab's N2-strain?
- State precisely which RNAi-clones were from which library and which ones were generated in house
- Also state in methods that generally, an RNAi-sensitive strain was used and knockdown was initiated

in L1
- How was the pph-4.1. promoter for the transgene defined?
- Specify the amounts of transgene/coinjection/filler DNA used for injection
- Stating that "large scale immunoprecipitation…" was performed as previously described is not sufficient. It should at least be stated whether worms lysed for IP/mass spec were also from a mixed stage population or synchronized and harvested as L4/adults etc
- Methods for stress survival assays state that animals were transferred to tBOOH at day 2 of adulthood, but legend for figure 3 says day 1. Clarify!
- How was transgene expression scored? Visually or by some image analysis method?
- Which concentration of FUDR was used?
- Which lysis buffer was used in the PhosTag SDS-PAGE and how much total protein was loaded?
- Instead of stating how many worms were bleached before mRNA isolation, it is more informative to mention how many L1s were plated and subsequently harvested for mRNA isolation
- Which RNA quality control steps were performed before library synthesis?
- How many worms were grown for Chip-Seq?
- How precisely are up/downregulated genes defined?

Reviewer #4 (Remarks to the Author):

The manuscript by Sen et al describes a mechanism through which SMK-1 may regulate the activity of DAF16/FOXO-dependent genes, which it appears to do through dephosphorylation of Spt5, a subunit of DSIF factor that in turn is involved in regulation of transcription initiation, Pol II pausing and elongation.

The work touches upon an important signaling pathway and appears to be quite novel and of general interest. The novelty of this study, in my opinion, is demonstration that a Pol II pausing factor that participates in transcription of all genes is a regulatory target for modulating only a specific pathway. The hypothesis proposed here could be quite compelling and has to do with PP4 complex dynamically regulating Spt5 phosphorylation levels, thereby maintaining Spt5 functionality. I am not entirely sure about the arguments made in support of this idea: they ether need to be clarified or the idea needs to be toned down.

The methodology proposed and used is for the most part adequate, and tests are rigorous. I have a question about quality of ChIP-seq datasets. Based on what's presented, it is impossible to determine if these ChIP-seq datasets are good or not. At the very least, some quality metrics should be presented, plus browser shots of relevant genes should be shown to show signal to noise ratio. It is not even clear what the sequencing coverage was. ChIP-sequencing presentation is definitely a weak point of this manuscript and needs to be remedied, hopefully with tools and information that authors already should have at hand.

On a related note, the shift of ChIP-seq peaks shown on metagene plots is not convincing and needs to be demonstrated through independent experimentation or analysis. For example, are there individual genes where this shift can be seen?

Another issue notable even without having access to additional data is normalization between different ChIP seq samples such as for figure 7. I am not sure it was adequately done as currently described, and certainly requires additional explanation.

1. Quote. "This phosphatase complex binds and dephosphorylates the transcription elongation factor SPT-5, and in
turn this supports efficient RNA polymerase II (Pol II) binding to promoter regions, transcriptional initiation, and eventually transcriptional termination and the release of Pol II and SPT-5 from

transcriptional end sites (TESs) under low IIS." If, as stated in Reference 34, SPT5 is phosphorylated to release paused Pol II into elongation, then what is the above statement (that dephosphorylation promotes these steps rather than inhibits them) based on? There seems to be a contradiction here unless I am misunderstanding the authors' explanation.

If PP4 complex dephosphorylates SPT5, this should in theory lead to inactivation of transcription. How come depletion of one, as stated in the abstract, phenocopies rather than antagonizes depletion of the other? I understand that a mechanism is possible wherein timely dephosphorylation of Spt5 is needed to get the turnover of SPT5 going. Is this is true, then Pol II should get stuck somewhere at the ends of genes, not at their promoter regions, no? As written, I fail to see a connection between the argument and the experimental outcome of PP4 depletion on a specific step of Pol II transcription. This may be a presentation issue, not a problem with experiments.

2. As I mentioned above ChIP-seq experiments need to be presented better. This is one of the most difficult molecular biology methods to get right and showing a metagene squiggle is not enough, and example genes should be shown at least in the supplement. Also, the tiny shift shown on these metagenes is not convincing because the shift distance is well within technical variations of DNA fragmentation and therefore must be presented better. Was the ChIP done in single-end or paired end format? If it is single end for a shorter sequencing run, then it is practically a nonstarter for this type of resolution even with opposite strand tag peak center approximation. Also, it may be my fault, but I may have missed the antibody that was used for ChIP of total Pol II (not S5P).

3. Do authors see docking of Pol II described by Baugh R et al, in 2013 and, if so, are there any changes on these genes?

4. I do not fully understand the section where PP4 complex is required for DAF-16 to promote resistance to one type of stress, but not the other (heat shock). This is an interesting observation, but it is not placed well in the context of the manuscript flow. The way I interpret this section is that DAF-16 is not at all the point of this manuscript. Rather, the point of this manuscript is PP4 complex' effect on what is essentially a general factor (SPT5) leading, surprisingly, to defects in specific genes rather than general effect. In other words, this result is certainly worth presenting, but it needs to be incorporated into the flow of the manuscript better.

5. Line 198 Supplement Fig 3b, c should be in the main figure, not in supplement. This is an important piece of data.

6. The last Results section "Dephosphorylation of SPT5" appears to be a standalone discussion with a mini introduction to boot. This verbiage may be hiding a crucial, and maybe the most important point of the manuscript, namely, the mechanistic consequence of SPT5 dephosphorylation on Pol II transcription. Authors suggest that this leads to changes on all steps of transcription. It is more likely that only one step is perturbed directly, and the rest are secondary, not direct, effects. In order for me (and reader) to see the data and arguments, this section needs to be shortened and clarified. There are strong conclusions made here, but the evidence is hidden behind too much wording and again squiggles of ChIP-seq traces.

7. On a related technical note, how was normalization made between different specimens In 7a, b, etc. ChIP normalization is a difficult task because differences in background will completely skew the data.

Minor issues.
1. In the abstract, loss of Spt5 is mentioned. Isn't this factor essential so its loss is hard to interpret. Did authors mean partial depletion?

2. “Phosphorylation of SPT-5 is crucial for efficient transcriptional elongation but otherwise absent during other stages of transcription. We propose that this absence is important and assured by PP4SMK-1. “ The importance of absence is a very confusing construct that makes little sense and muddles understanding of the abstract and the manuscript. How can the absence be required for transcription initiation, and termination?

3. In the abstract, a statement is made of genes that are strongly transcription initiation dependent. This statement makes no sense because all genes are absolutely and unequivocally transcription initiation dependent. Genes may have stronger or weaker promoters though, for example.

4. What does this mean: “significant part of DAF-16 mediated gene expression changes”?

5. The statement that PP4 confers its phenotype by” licensing the transcriptional regulation of a subset ” of genes… appears to carry no useful information and essentially reads as if it regulates a subset of genes by regulating a subset of genes.

6. In Figure 4A, if I am understanding it correctly, an argument is made about compensatory restoration of gene expression. This is impressive, but what should be clarified is how many genes (what %) restored is the threshold for a reader to get excited. I thought that 18.7% is a low number, but maybe not.

Letter to the referees:

To begin, we would like to thank the reviewers for their efforts in assessing our manuscript, for appreciating the value of our work, and for their constructive feedback. In the following, we have responded to all the reviewers' comments and have also referred to any changes or additions that we made to the manuscript. We hope that the reviewers will be pleased with the revisions we made.

Comments by the reviewers:

Reviewer #1:

> *In this manuscript entitled "DAF-16/FOXO requires Protein Phosphatase 4 to initiate the transcription of stress resistance and longevity promoting genes", Sen et al., set to identify how SMK-1 regulates low insulin/IGF signaling (IIS). They use C. elegans and identify that SMK-1 is a component of a specific PP4 complex isoform, and this complex is essential for DAF-16-driven gene expression. Additionally, the authors show that loss of PP4(SMK-1) impairs PolII recruitment and transcriptional initiation & termination. Furthermore, they propose that SPT-5, a conserved transcriptional regulator, is a probable substrate for SMK-1. In sum, this is very interesting and, in most areas, extremely well executed work. However, the mass spectrometry part is not adequately described and, in many parts, contain contains possible mistakes, requiring revision. Also illustrating that SPT-5 is directly substrate for PP4(SMK-1) and if the dephosphorylation all the four sites (671, 677, 882 and 904) is needed for the effect.*
>
> *Major quibbles*
>
> - *The mass spectrometry parameters, especially the database search parameters are not adequately described, as well as the tables contain very little actual information and do not fulfill the minimum information needed to be reported with this type of analyses.*

Our response: We have now substantially extended our description of the mass spectrometry parameters used for protein ID and for the phosphoproteomic analyses in the respective methods sections, including also better information about the conducted database searches. Further, we have added additional content to the results tables of the protein ID experiments in Figure 1b, and the Supplementary Tables 2, 3, and 7 (formerly Supplementary Tables 2, 3, and 4), so that the tables now display not only spectral counts and molecular weight of the identified proteins but also the number of unique peptides and the protein coverage. Finally, we have updated Supplementary Table 7 (formerly Supplementary Table S4 in the original manuscript) according to the reviewer's request (see later comment below), to now state the actual identified phosphopeptides and phosphorylation probabilities for the contained sites.

> *More specifically:*
>
> - *What does the authors mean with large-scale IP?*

Our response: We refer here to IP experiments that were conducted from unusually large amounts of input material, typically 20-50 grams of *C. elegans*, to facilitate the identification of low abundant co-purifying proteins by mass spectrometry. In contrast, we would use only 1-2 grams of *C. elegans* for a normal co-IP experiment in which we detect the proteins by western blotting. Nevertheless, we understand the reviewer's concern that the term "large-scale" may be ambiguous in this context, and thus we removed it from the manuscript.

- *Who many replicate analyses were used in Fig.1b?*

Our response: We conducted here two independent experiments that gave consistent results – meaning they both revealed SMK-1 as part of the same PP4 complex. Figure 1b shows the results for only one of these experiments. We have now added the results of the second experiment as Table S2 and also refer to this table in the Figure legend to Figure 1b.

And how were the data specific interactors filtered using the untagged control?

Our response: To remove contaminating proteins from the lists of co-purifying proteins, we conducted control purifications from wild-type (untagged) *C. elegans* and considered any proteins identified in these control purifications as contaminants. We removed them from the lists of co-purifying proteins using the software Contrast (Tabb et al., 2002). We also added this information now to the methods section.

- *Figure 1b, with how many unique peptides the proteins were identified?*

Our response: We now added this information to Figure 1b.

o Supplementary Table 2 and 3 state that "Only proteins identified by at least five unique peptides are shown.". Is this correct or are the authors mixing up "unique peptides" and "spectral counts". These are not the same thing.

Our response: This actual statement is correct. We indeed only show proteins identified by at least five unique peptides. However, we did a mistake here and would like to thank the reviewer for pointing it out to us: We accidentally presented the unique peptide count data as the spectral count data in the submitted table. We have now corrected both, Supplementary Tables S2 and S3. The tables (in the new manuscript they are now Supplementary Table 3) now correctly display the spectral counts, the unique peptide counts, and we also newly added protein coverage information.

o The Supplementary Table 4 contains again very little information. Are all the four STP-5 phosphosites (671, 677, 882 and 904) detected from different peptides or from the same? The peptide sequences and site probabilities for the modification would be needed.

Our response: We have substantially updated this table (it is now Supplementary Table 7), so that it now states the actual phosphopeptides (including sequences) and site probabilities for the modifications. From

this improved presentation of the data it now is also apparent that the SPT-5 phosphosites 671 and 677 were detected from the same peptide, while phosphosites 882 and 904 were detected from different peptides.

o Additionally Supplementary Table 4 (Proteins containing phosphorylations that were seen only in the absence of smk-1) has mistakes in the last column (Phos site within isoforms).

Our response: As stated already before, we have now substantially updated this table (it is now Supplementary Table 7), so that it states the actual phosphopeptides that were increasingly phosphorylated in the absence of $PP4^{SMK-1}$. With this new information provided, we don't see a need for this last column any longer and thus have entirely removed it.

Minor quibbles

- To convincingly illustrate their proposed mechanism(s), the authors that should show which of the SPT-5 phosphosites are direct targets for PP4(SMK-1) and which of these are needed for SPT-5 activity.

Our response: To address whether any of the phosphosites that we had identified are direct targets of $PP4^{SMK-1}$, we conducted *in vitro* phosphatase assays: First, we purified either the endogenous $PP4^{SMK-1}$ complex from *C. elegans* or purified a recombinantly expressed *C. elegans* PPH-4.1–PPFR-2–SMK-1 complex from *S. cerevisiae.* Then we tested the ability of these complexes to dephosphorylate a variety of synthetic phosphopeptides harboring the different $PP4^{SMK-1}$-dependent SPT-5 phosphorylation marks that we identified in our phosphoproeomic analyses. As negative control we used a PP2A substrate peptide that should not be dephosphorylated by $PP4^{SMK-1}$. We found that out of S671, S677, S882, and S904, only S677 and S882 were efficiently dephosphorylated by $PP4^{SMK-1}$ *in vitro* and thus likely to be direct targets. We have added this new data to the manuscript as Supplementary Figure 9c, e, f.

To address whether any of the $PP4^{SMK-1}$-dependent phosphorylation sites are important for SPT-5 function, we tried to make individual S/T to A mutations of the four sites in SPT-5. We generated expression constructs of *spt-5* with the respective mutations under the endogenous *spt-5* promoter and injected them into wild-type animals. Unfortunately, we were unable to obtain stable transgenic lines. Given that *spt-5* is an essential gene, this observation would though be consistent with those phosphorylation sites and their regulation being crucial for SPT-5 function.

Reviewer #2:

This manuscript explores the mechanism by which SMK-1 modulates output of the transcription factor DAF-16 to promote longevity and confer stress resistance in C. elegans. It has long been recognized that DAF-16 is a key effector of the increased lifespan and enhanced stress tolerance of animals maintained under conditions of low insulin/IGF signaling (IIS). SMK-1 was identified as a potent upstream regulator of this signaling, but the underlying mechanism was unknown. Here the authors perform mass spectrometry with a tagged version of SMK-1, and identify subunits of

the protein phosphatase 4 (PP4) complex as interaction partners. By genetic and RNAi-based manipulations, they make the case that PP4 and SMK-1 are in a common pathway, upstream of DAF-16, which regulates lifespan under low IIS conditions (experimentally induced by a mutation in DAF-2, the IGF receptor). Moreover, as is the case with SMK-1, RNAi targeting PP4 partially ablates DAF-16-dependent resistance to oxidative stress and UV, but has no effect on the response to heat stress, consistent with a specific role of both SMK-1 and PP4 upstream of DAF-16. To identify potential downstream targets of SMK-1/PP4, the authors perform a phosphoproteomic analysis after SMK-4 knockdown, and identify the conserved transcription elongation factor SPT-5 as a putative PP4 target; SPT-5-derived phosphopeptides are enriched in SMK-1-depleted animals, relative to non-targeted controls. Supporting SPT-5's participation in a DAF-2-PP4-DAF-16 pathway, RNAi targeting SPT-5 had similar, selective effects on lifespan and both oxidative and genotoxic stress responses. SMK-1 knockdown, moreover, had effects on RNA polymerase II chromatin distribution (measured by ChIP-seq) that are at least broadly consistent with derangement of known functions of SPT-5 in pausing and elongation. This is a novel and in many ways attractive mechanism, and the genetic data suggesting functional links among these players are mostly convincing, but there are significant gaps and omissions, and some dubious interpretations, which would need to be corrected before I could recommend publication. These might best be addressed, in most cases, by softening or removing some of the mechanistic conclusions and discussing alternative interpretations. In at least one case (point 1), however, additional experimental support will be needed.

My specific concerns are:

1. The purported association of SMK-1 with PP4 catalytic and regulatory subunits in C. elegans is based almost entirely on the mass spectrometry (Fig. 1a, b); the only independent "validation" of this interaction is done in human HEK293 cells overexpressing a GFP-tagged version of the human ortholog SMEK1 (Fig. S5). The model in Fig. 1c posits SMK-1 as an intrinsic component of a functional PP4 complex, so it is critical to have some additional support for this idea.

Can this complex be reconstituted from recombinant subunits?

Our response: We addressed this by the following experiment: First we recombinantly co-expressed the core subunits of the *C. elegans* $PP4^{SMK-1}$ complex, PPH-4.1, PPFR-2 and SMK-1, in *S. cerevisiae*. Next, we purified PPH-4.1 by TAP-purification and looked whether the complex had assembled in this heterologous system, i.e. by testing through western blotting, if SMK-1 had co-purified with PPH-4.1. Our new Supplementary Figure 9d shows that this was indeed the case. We further then used this purified recombinantly expressed complex for *in vitro* phosphatase assays, which yielded results identical to the purified endogenous complex from *C. elegans.* The results of these phosphatase assays can be found in Supplementary Figures 9c-e.

We conclude that also recombinantly expressed PPH-4.1, PPFR-2, and SMK-1 can assemble into a phosphatase complex, at least in *S. cerevisiae*, which is catalytically active and sufficient to dephosphorylate SPT-5 at multiple sites, with an activity and specificity similar to the endogenous $PP4^{SMK-1}$ complex from *C. elegans*.

What is the stoichiometry of the interaction?

Our response: Determining the subunit stoichiometry of the *C. elegans* PP4^{SMK-1} complex would be a very laborious task, and we believe that knowledge about this stoichiometry would not drastically enhance the current manuscript. Thus, we decided to refrain from experimentally addressing this point. Nevertheless, numerous studies on orthologs of the PP4^{SMK-1} complex, in particular in human, suggest that PPH-4.1, PPFR-2 and SMK-1 associate in a 1:1:1 ratio (Hastie et al., 2000; Lee et al., 2010). Since the subunit stoichiometries within related phosphatase complexes, e.g. of PP2A, tend to be conserved across species, it may well be that in *C. elegans* PPH-4.1, PPFR-2 and SMK-1 associate in a 1:1:1 ratio, too. However, future work will have to evaluate this.

How does SMK-1 association affect PP4 activity?

Our response: We have addressed this again by *in vitro* phosphatase assays. We recombinantly expressed the catalytic subunit of the *C. elegans* PP4^{SMK-1} complex, PPH-4.1, alone in *E. coli*, purified it, and compared its substrate specificity to the PP4^{SMK-1} holocomplex which we purified from *C. elegans* and to the PP4^{SMK-1} holocomplex that we had expressed recombinantly and purified from *S. cerevisiae*. As substrates, we used a variety of synthetic phosphopeptides harboring the different PP4^{SMK-1}-dependent SPT-5 phosphorylation marks that we identified in our phosphoproteomic analyses as well as a PP2A substrate peptide that should not be dephosphorylated by PP4^{SMK-1}. As can be seen in Supplementary Figures 9c, e and f, the holocomplex of PP4^{SMK-1} that contains SMK-1 has a clear substrate specificity, dephosphorylating only S677 and S882 of SPT-5, while leaving the other two SPT-5 sites as well as the PP2A model substrate untouched. In contrast, PPH-4.1 alone lacks the substrate specificity of PP4^{SMK-1}: It significantly dephosphorylates not only SPT-5's S677 and S882 positions but also S904 and it dephosphorylated the PP2A model substrate. From this we conclude that the non-catalytic subunits of the PP4^{SMK-1} complex, including SMK-1, add substrate specificity to the complex, which would explain also their importance *in vivo*.

I realize these are not trivial issues to address in C. elegans, where (I presume) antibodies to the relevant proteins are not widely available, but some additional characterization of the putative PPH-4/PPFR-2/SMK-1/PAA-1 complex is needed. Do these proteins co-purify (e.g. by size exclusion chromatography or sedimentation)?

Our response: We first would like to point out that we observed the co-purification of SMK-1 with PP4 subunits many times, in multiple IP-mass spec experiments from *C. elegans*, using also two different tags on SMK-1 and resins for the IPs, namely an mCherry tag with anti-mCherry-antibody resin or a GFP tag with GFP-trap resin. Thus, the co-purification of SMK-1 with other PP4 subunits in *C. elegans* is unlikely to be an artifact. Further, the presence of SMK-1 in PP4 complexes could repeatedly be shown in other species. As already mentioned by the reviewer, we were able to co-IP human SMEK1 with the PP4 catalytic subunit from HEK293 cells. Similarly, the labs of Ruedi Aebersold and Judy Lieberman both found human SMK-1 to be part of PP4 complexes, and the Aebersold lab further confirmed the presence of the SMK-1 ortholog Psy2p in PP4 complexes in yeast (Chowdhury et al., 2008; Gingras et al., 2005).

Nevertheless, to provide further evidence that SMK-1 incorporates into PP4 complexes, we also conducted a new experiment that we already mentioned above: We expressed *C. elegans* SMK-1, PPFR-2, and PPH-4.1 recombinantly in *S. cerevisiae* and purified PPH-4.1 by use of a TAP-tag, observing that also under such heterologous expression conditions and when pulling now on the catalytic subunit instead of SMK-1,

SMK-1 co-purifies with PPH-4.1. Taken together, our and published work shows that a $PP4^{SMK-1}$ complex exists in many species and circumstances.

If no further biochemical analysis is feasible, can the genetic argument for their concerted function be strengthened (i.e., by testing for epistasis between SMK-1 and PPH-4.1/PPH-4.2 RNAi)?

Our response: In addition to our new biochemical confirmation of the $PP4^{SMK-1}$ complex, we also conducted lifespan assays, testing for epistasis between *smk-1* and *pph-4.1/pph-4.2*. Such analysis could not be conducted in a perfect manner, given that only for *pph-4.1* we had a null allele and had to rely on RNAi for the loss of *pph-4.2* and *smk-1*. Nevertheless, as can be seen in the new Supplementary Figure 2e, the phenotypes of *smk-1* and *pph-4.1/pph-4.2* loss are mostly non-additive. This data is consistent with *smk-1* being epistatic to *pph-4.1/pph-4.2*, functioning in the same genetic pathway.

2. Of the four sites within SPT-5 that become hyperphosphorylated upon SMK-1 knockdown, two are within the conserved P-TEFb target region (CTR/CTD), but neither is a canonical, "proline-directed" (S/T-P) site, of which there are many (mostly T-P) within the C. elegans protein. This is important context for trying to explain the effects of SMK-1 versus SPT-5 depletion, which the authors don't acknowledge. In my opinion there is a strong likelihood that the phosphorylation events apparently being reversed by PP4/SMK-1 are playing a different role from that of the classical CTR/CTD phosphorylations described in previous papers. (Presumably, SPT-5 is still being phosphorylated on all those other sites.)

Our response: We agree with the reviewer that P-TEFb has previously been described as a "proline-directed" kinase, preferentially targeting S/T-P sites (Garriga et al., 1996). Consistently, it has been shown to phosphorylate SPT-5 predominantly at T-P sites in the repeats of its CTD – an event that promotes transcriptional elongation (Ivanov et al., 2000; Yamada et al., 2006). Unfortunately, the phosphorylation state of these T-P sites could not be quantified in our mass spec analyses, making it impossible for us to determine, if $PP4^{SMK-1}$ counteracts these phosphorylation events. Instead we observed $PP4^{SMK-1}$-dependent dephosphorylation of two other "not-proline-directed" sites (positions 882 and 904) of the CTD, and of two sites (positions 671 and 677) that are "proline-directed" (S-P) but located upstream of the CTD. The phosphorylating kinases for these other sites are not known and their physiological roles are not clear.

Taken together, we agree with the reviewer that it remains somewhat uncertain, to what extent $PP4^{SMK-1}$ acts by directly reverting P-TEFb-mediated phosphorylation of SPT-5's CTD and its transcriptional elongation promoting effects, or if it acts by different phosphoregulatory mechanisms. We still view the idea of $PP4^{SMK-1}$ counteracting P-TEFb as the most likely scenario but need to consider other mechanisms, too. Thus, we now adjusted the manuscript, to accommodate for the possibility of such alternative mechanisms but refrain from speculating about their exact nature. Future studies will have to explore this matter in more detail.

3. I found the interpretation of SMK-1 RNAi effects on Pol II distribution (Fig. 5b-d) to be generally unpersuasive. The authors suggest the biphasic distribution of ChIP-seq signals around the TSS of DAF-16-/SMK-1-dependent genes is evidence of Pol II in two different stages of transcription: paused and pre-initiated. Is the latter analogous to the "docked" Pol II described in ref. 19? The

effect of SMK-1 RNAi on this pattern is a general reduction in Pol II cross-linking, with maybe some skewing towards more Pol II upstream of the TSS, which the authors interpret as an effect on initiation. Whether or not Pol II has initiated cannot be deduced from ChIP-seq analysis alone, nor is Ser5 phosphorylation of the Pol II CTD (5d) a reliable marker of initiation. Other interpretations need to be considered as the basis for this distribution, e.g. divergent transcription.

Our response: We are unsure, if the "pre-initiated" RNA Pol II that we describe is analogous to the "docked" RNA Pol II, as described in ref. 19 (Maxwell et al., 2014). In this prior study, mostly growth-promoting genes showed this docking; and they are distinct from the genes that are co-activated by DAF-16 and SMK-1.

We also agree with the reviewer that there can be other interpretations of our Pol II-ChIP-seq data besides a role for PP4^{SMK-1} in Pol II recruitment and transcriptional initiation. Indeed, one of these alternative mechanistic explanations could lie in a previously observed role of Spt-5 in preventing antisense/divergent transcription (Core et al., 2008; Neil et al., 2009; Shetty et al., 2017). Such divergent transcription could lead to impaired gene expression and more Pol II localization upstream of the TSS. We now mention the possibility of such alternative mechanistic interpretation in the revised version of the manuscript. Nevertheless, we deem this explanation less likely than a defect in transcriptional initiation: The work by Shetty et al suggests that antisense/divergent transcription inferred by SPT-5 loss should also lead to accumulation of Pol II just downstream of the TSS and thus should yield a significantly higher pausing index, which we do not observe in *smk-1* RNAi conditions (Supplementary Table 6). Furthermore, we believe that divergent transcription should not interfere with phosphorylation of the RNA Pol II CTD at Ser5, which stands in conflict with our observations in Figure 5d.

4. Similarly, the authors argue that SMK-1 RNAi leads to impaired termination, based on a very modest shift of the Pol II and SPT-5::GFP ChIP-seq peaks downstream of the "TES" (which I presume is actually the polyadenylation signal, i.e., not where Pol II is actually terminating). This is another case of trying to fit the data into a pre-existing model, which was based on measurement of actively transcribing Pol II, as opposed to simple occupancy (ref. 33). I note that SMK-1 RNAi is having little or no effect on the position where Pol II or SPT-5 ChIP signals are returning to baseline downstream of the "TES," suggesting there might be little effect on termination per se.

Our response: The effect of PP4^{SMK-1} loss on transcriptional termination may not be tremendously strong, e.g. when compared to the observations made in fission yeast PP1 mutants (Parua et al., 2018), but we still think that it is very notable. The scale of Figure 7c may disguise this a bit, but the ChIP signals under *smk-1* RNAi return to baseline not at the same position but about 30-50bp downstream from where the control conditions return to baseline; and even more notably, there is a shift of more than 100 bp for the Pol II and SPT-5 summits at the TES. Taken together, we still see this as relevant phenotypes and believe that transcriptional termination is to some extent influenced by absence of PP4^{SMK-1}. Nevertheless, we have further softened our wording in the manuscript, clarifying that these phenotypes are "moderate" and "mild".

Regarding the definition of the "TES": This is indeed not the site where Pol II is terminating or dissociating from the DNA. It is the site where the transcribed region of the mRNA ends and polyadenylation begins – as annotated in the Ensembl genome annotation for *C. elegans*. We use this terminology, as it also is used by ngs.plot (Shen et al., 2014), the software that we used to generate the analyses displayed in Figures 7c,d. For further clarification, we have now added the definition of the "TESs" used in our manuscript to the respective methods section.

5. There is an apparent (slightly counterintuitive) loss of SPT-5 recovery in SMK-1 IPs from low IIS (Supp. Table 2) relative to high IIS mutants (Supp. Table 3). Do the authors believe this to be significant? If so, could they say something about it? (If not, explain why it is not.)

Our response: We actually doubt that this observation is significant; and we agree that the observation would be counterintuitive. Future studies would have to evaluate this further.

6. Why is the effect of the ppfr-4 RNAi on lifespan of the daf-2 daf-16 mutant biphasic (Supp. Fig. 1b)? (This is a minor point.)

Our response: We would like to thank the reviewer for pointing this out. This was a very large lifespan experiment that included many conditions. Upon closer inspection of the original data, we realized that specifically one of the *ppfr-4* RNAi replicate plates had an unusually high censoring rate, above 20% - mostly due to bursting. This indicates some type of experimental error on this plate that already impaired development of the animals and thus makes the resulting lifespan data unreliable. We therefore removed this replicate plate. In turn, this eliminated the biphasic effect of *ppfr-4* RNAi on lifespan, as can be seen in the updated version of the figure (now Supplementary Figure 2b).

7. There are a number of places in the manuscript where figure call-outs are omitted, making it harder to follow, for example, in line 129 (should be Fig. 2c, I think).

Our response: We apologize for this omission and have now added this figure call-out to the former line 129. Further, we have now carefully gone through the entire manuscript and made sure that figure call-outs are present wherever needed and appropriate.

8. The abbreviation "t-BOOH" first appears in line 154 but is not defined here (or anywhere).

Our response: "t-BOOH" stands for "tert-Butyl hydroperoxide". We have now added this definition at the first occurrence of "t-BOOH" in the manuscript.

9. Although I am skeptical about the mechanistic interpretations of Pol II distributions in Fig. 5 (see point 3), the difference between SMK-1-dependent and –independent, DAF-16-dependent genes is very interesting. It should be quantified, e.g. by comparing the pausing index (PI) of these two classes (to each other and to all genes). Is SMK-1-dependence significantly correlated with low PI?

Our response: We now have calculated the pausing index for the gene sets presented in Figure 5. Since different publications chose different windows for paused and elongating RNA Pol II on the DNA, we actually calculated two pausing indices: The first "relaxed" index is a more conventional index that considers all Pol II in a window from -500 to +500 around the TSS as paused Pol II and compares it to the elongating Pol II (Pol II within a window of +500 from the TSS to the TES). The second "stringent" index excludes Pol II that is localized upstream of the TSS and thus has not reached the pause site yet, by comparing Pol II within a narrower window of 0 to +500 from the TSS to the elongating Pol II (Pol II

within a window of +500 from the TSS to the TES). As can be seen in the new Supplementary Table 6, both indices behave quite similar:

We find that when investigating the bulk of DAF-16-activated genes, these genes show a significantly lower pausing index when compared to all genes in the genome ($p<0.05$, t-test). However, co-dependence on SMK-1 does not significantly affect the pausing index further ($p<0.05$, t-test).

10. To assess whether putative substrates of PP4 are contributing to its regulation of DAF-16-dependent gene expression, the authors use a reporter assay, results of which are presented in semi-quantitative form in Fig. 6b ("+" vs. "++" vs. "blank"). I would recommend showing the actual data, at least for "positive" targets.

Our response: We now provide actual images of the phenotypes observed with the *sod-3P::GFP* reporter in wild-type and *daf-2(e1370ts)* backgrounds. Even though we had to refrain from showing images for every single potential target that gave a positive result in the reporter assays, as this figure would have gotten too large in size for display on a single page, we present all conditions that matter for the conclusions of our manuscript: We show all control conditions as well as *spt-5* RNAi in both, the wild-type and *daf-2(e1370ts)* backgrounds. These images can now be found in the new Supplementary Figure 8a.

11. As the authors point out in the discussion, SPT-5 is an ancient, highly conserved and universally essential elongation factor engaged at most or all Pol II genes, yet its knockdown "had no effect in wild-type or daf-16 null mutants" (line 300, Fig. 6c-f). A likely explanation is residual SPT-5 protein; is there any way to assess the efficiency of protein (or, failing that, mRNA) depletion?

Our response: Indeed, SPT-5 is a highly conserved elongation factor that also has other functions beyond regulating DAF-16-targeted genes. Consistently, homozygous null mutants of SPT-5 do not exist, and we find that knockdown of SPT-5 by RNAi from the L1 stage results in larval arrest around the L2/L3 stages. In the shown experiments, we could deplete SPT-5 only from the L4 stage onwards, to avoid a general impact on the animals' viability and eventually be able to observe this DAF-16-specific phenotype; and even this was likely only possible due to a residual amount of SPT-5 remaining present in these animals throughout adulthood, which would suffice for the animals' viability and for wild-type and *daf-16* mutant animals to achieve a normal lifespan. To investigate this experimentally, we tested the effect of L4-initiated *spt-5* RNAi on the expression of a SPT-5::GFP transgene at day 2 of adulthood using Western blotting. As can be seen in Supplementary Figure 9a,b, we achieved an excellent knockdown efficiency of about 92%; but it also means that nearly 8% of the protein remain expressed, which likely is then insufficient to support DAF-16 target gene expression in *daf-2* mutants but sufficient to fulfill the more general functions of SPT-5, allowing for normal transcription throughout the rest of the genome.

12. Can the authors cite a reference for the statement that "SPT-5's CTD is unphosphorylated…until it has traveled with Pol II to the pause site" (lines 319-20)? This seems plausible by analogy with the Pol II CTD, but I am not aware of any data to that effect.

Our response: This has been shown by ChIP-seq in fission yeast (Parua et al., 2018). Furthermore, phosphorylation of SPT5's CTD is one of the steps triggering release of Pol II from the pause site (Adelman and Lis, 2012). Thus, it would make little sense for SPT5 to already arrive at the pause site with a phosphorylated CTD. SPT-5 would otherwise be insensitive to the elongation signals by P-TEFb.

13. There are other, similar instances in which the authors' mechanistic statements and inferences go well beyond what has been shown in the literature or by their data, for example, saying that SPT-5 CTD dephosphorylation is needed for Pol II and DSIF recycling and reinitiation (lines 325-7), that SPT-5 phosphorylation needs to be low during promoter clearance (lines 332-3), and so on. The last two paragraphs of Results (lines 328-66) can (and probably should) be re-written to say, simply, that "perturbations of SMK-1 alter Pol II and SPT-5 distribution on DAF-16/SMK-1-responsive genes, possibly through SMK-1 association with PP4, in a manner broadly consistent with known roles of SPT-5 in transcription elongation and termination."

Our response: We have now substantially adjusted the manuscript to correct or remove any mechanistic statements and inferences that may go beyond the data and literature, paying particular attention to the former lines 325-7, 332-3, and 328-66.

14. In the Discussion, the authors speculate (their word) that the proposed mode of DAF-16/SMK-1 gene regulation at the initiation step might allow rapid induction in response to stimuli (lines 418-9). It is worth pointing out that the exact same rationale has been proposed for why certain genes are pause-regulated. I suspect that neither generalization is entirely accurate, so this is probably best left unsaid at this point.

Our response: We very much respect the reviewer's opinion in this matter. However, our manuscript goes through extensive effort to identify a novel mechanism, by which DAF-16 and $PP4^{SMK-1}$ regulate expression of a specific set of genes in response to low IIS. Naturally, questions about the purpose of this novel mechanism will immediately arise for any reader of our work, and we find it important, even though we don't have a reliable answer for them, to at least mention our preferred hypothesis. This is what we do, by saying that this mechanism may allow for rapid transcriptional induction in response to stimuli; and it even is supported by published work, showing in a different context that SPT-5's impact on transcriptional induction can be important for expression of rapidly-induced immune response genes (Diamant et al., 2016). Knowing though about the uncertainty of our hypothesis, we clearly mark it as "speculative" in the text, and we further added a sentence, stating that this matter requires more detailed investigation in the future.

Reviewer #3:

In their manuscript entitled "DAF-16/FOXO requires Protein Phosphatase 4 to initiate the transcription of stress resistance and longevity promoting genes", Sen et al. propose a model that explains how a long-known regulator of lifespan and of the longevity-promoting transcription factor DAF-16, SMK-1, actually exerts its effects on DAF-16 target gene expression and thus, longevity in the nematode C. elegans. Using a combination of mostly high throughput biochemical/proteomics and molecular biological/genomics-transcriptomics approaches, as well as genetic approaches in C. elegans, the authors (1) provide evidence for SMK-1 indeed being a subunit of PP4-complex(es); (2) show that PP4-subunits are required for full longevity of long-lived C. elegans daf-2 mutant/RNAi worms and that they act in a common pathway with daf-16; (3) define the stress resistance pattern of PP4/SMK-1 compromised worms; (4) provide evidence for PP4/SMK-1 being required for efficient RNA Pol II promoter recruitment of DAF-16/SMK-1 coregulated genes and (5) provide evidence for SPT-5 being the critical target of PP4/SMK-1

whose dephosphorylation is important for proper transcription at a subset of DAF-16 target genes. These findings are novel, of interest to experts in the field of C. elegans aging research, and may have implications for other organisms as the molecular regulators under investigation are conserved in humans and the authors even provide evidence for physical interaction between the human orthologs of SMK-1 and SPT-5. The wealth of experiments conducted collectively supports all conclusions drawn, although suboptimal controls were used in some cases and low-throughput validation of high throughput proteomics/RNAseq-data is not provided. Moreover, the methods section lacks important experimental information that would be of interest to experts in the field. Thus, the manuscript requires some revision to warrant publication in Nature Communications.

Specific issues:

Introduction
Line 47: "...where it [DAF-16] eventually binds to the promoters of many stress resistance and longevity promoting genes and drives their transcription". Misleading. DAF-16 appears to also repress the expression of certain lifespan-limiting genes (Murphy et al., Nature volume 424, pages 277–283 (17 July 2003)) □ rephrase

Our response: While it has been shown that DAF-16 is predominantly a transcriptional activator (Riedel et al., 2013), we agree with the reviewer that DAF-16 can repress some target genes, too. Thus, we have rephrased now this sentence to say "…where it eventually binds to the promoters of stress resistance and longevity controlling genes and regulates their transcription."

Section SMK-1 is part of a nuclear Protein Phosphatase 4 complex (PP4SMK-1) Interaction of SMK-1 with PP4-subunits was detected by large-scale IP/mass spec and confirmed by another large-scale IP/mass spec experiment using a different tag on SMK-1. A low throughput IP/Western Blot experiment would be more standard as a confirmation. Why was this not done? In principle, the authors have SMK-1 and PPH4-4.1 with different affinity tags.

Our response: The only distinctly epitope-tagged versions of SMK-1 and PPH-4.1 that we have are SMK-1::mCherry and PPH-4.1::GFP. We never attempted co-IP/Western blot experiments to confirm their interaction, because mCherry and GFP are closely related proteins. This makes it difficult to conduct clean specific IPs of one tag without the affinity matrix showing some affinity also for the other tag. Thus, IP/mass spec experiments were the better choice for us.

Nevertheless, we meanwhile generated some co-IP/Western Blot data, although in a heterologous system, to further confirm the physical interaction between SMK-1 and PPH-4.1. We expressed *C. elegans* SMK-1-HA3, PPH-4.1-TAP, and PPFR-2 in *S. cerevisiae*, lysed these cells, and then purified the PPH-4.1-TAP using IgG resin. By Anti-HA Western Blotting, we observed that SMK-1-HA3 co-purified with PPH-4.1-TAP. This new data can now be found in Supplementary Figure 9d.

Line 85: "At the same time, given that SMK-1 is required for DAF-16 functions specifically when IIS activity is low,…" Misleading: Wolff et al. (ref. 6 cited by the authors) also show that smk-1 is required for the longevity of germline-ablated animals. Even though germline-longevity is also dependent on daf-16 (Hsin and Kenyon, 1999), several lines of evidence (esp. Lin et al., 2001, Berman and Kenyon 2006) suggest that regulation and/or function of daf-16 by germline signals differs from that in/by reduced IIS □ rephrase

Our response: We fully agree with the reviewer that SMK-1 is not only required for DAF-16 functions when IIS is low but also under other circumstances, e.g. for promotion of longevity by absence of a germline. However, in the given sentence we did not try to state otherwise. To better prevent misinterpretation, we now changed the sentence to say "At the same time, given that SMK-1 is required for DAF-16 functions when IIS activity is low but not when it is high,…"

Line 94-96: The detection of PAA-1 in SMK-1 IPs is not necessarily a hint towards PAA-1 being part of a PP4-complex. Could be the other way round, namely SMK-1 being part of a PP2A-complex. Generally, from IP/MS-experiments, the authors cannot conclude which co-precipitating proteins are part of which out of potentially several SMK-1 complexes phrase more carefully

Our response: We agree with the reviewer that PAA-1 does not necessarily have to be part of a PP4 complex but may be part of a distinct SMK-1 containing complex, i.e. a PP2A-related complex. We have now adjusted the manuscript accordingly and have also removed some of the manuscript's attention on PAA-1, e.g. by eliminating PAA-1 from Figure 1c and the discussion section. Future work will have to determine, why PAA-1 is found in SMK-1 purifications.

Fig. 1a. Labeling a worm strain not expressing the SMK-1::mCherry transgene as "untagged" is misleading as it in the first place suggests that it expresses a transgene containing plain SMK-1. Simply call it "control".

Our response: We corrected this now in the manuscript, calling the negative control sample "Control".

The more appropriate IP-control would have been a strain expressing mCherry (without the SMK-1 ORF) from the same promoter. Thus, it is absolutely necessary that the authors validate their IP by another approach. Unfortunately, the chosen one (different tags but also IP/MS) lacks the information of what the "control purification" was in this case. As indicated above, a low throughput IP/Western Blot experiment would be desirable.

Our response: The negative controls for all IP/mass spec experiments were purifications with the same affinity resin from lysates of animals that did not express a transgene.

To provide additional support for the existence of a $PP4^{SMK\text{-}1}$ complex, we first would like to point out that the presence of SMK-1 in PP4 complexes could repeatedly be shown in other species. In Supplementary Figure 8c, we were able to co-IP human SMEK1 with the PP4 catalytic subunit from HEK293 cells. Similarly, the labs of Ruedi Aebersold and Judy Lieberman both found human SMK-1 to be part of PP4 complexes, and the Aebersold lab further confirmed the presence of the SMK-1 ortholog Psy2p in PP4 complexes in yeast (Chowdhury et al., 2008; Gingras et al., 2005). To though further support that SMK-1 incorporates into PP4 complexes, we now conducted a low throughput IP/Western Blot experiment, which we already mentioned above: We expressed *C. elegans* SMK-1-HA_3, PPFR-2, and PPH-4.1-TAP recombinantly in *S. cerevisiae*, purified PPH-4.1 via its TAP-tag, and eventually evaluated this pulldown experiment by Western blotting against the TAP and HA_3 tags. We found that also under such heterologous expression conditions SMK-1 co-purifies with PPH-4.1, further confirming the interaction of SMK-1 and PPH-4.1 and thus supporting the existence of a $PP4^{SMK\text{-}1}$ complex.

Fig. 1d: only 1 worm shown, i.e. it is not possible to estimate how robust the localization pattern is across the population would be great if images with multiple worms were provided; moreover, IIS is known to regulate longevity during adulthood (Dillin et al, Science Vol. 298, Issue 5594, pp. 830-834) images from day 1 adults would be more informative than L4 larvae.

Our response: We now provide images showing multiple day 1 adult worms for each condition as a new Supplementary Figure 1.

Supplementary table 2/3: As the results are presented in different tables, it is difficult to compare the lists and see that the spectral count numbers are very similar for the highlighted proteins combine in one table.

Our response: We have now combined the former Supplementary tables 2/3 into one Supplementary table (now called Supplementary Table 3).

Section PP4SMK-1 is required for DAF-16 to promote longevity Line 132: "Next, we validated the phenotypes obtained from knockdown of the PP4SMK-1 catalytic subunits by use of a null mutant allele of pph-4.1." What is the evidence for the allele used being a null (i.e. complete loss of function)?

Our response: The reviewer is correct. Formally it has not been proven that *tm1445* is a complete null allele of *pph-4.1*. However, *tm1445* has been fully sequenced, lacking exons 3 and 4 of *pph-4.1*. Exon 4 contains the active site of this phosphatase. Thus, any potential protein encoded by *pph-4.1(tm1445)* would be void of phosphatase activity; and we assume that this makes PPH-4.1 unable to fulfill its physiological roles. Nevertheless, we have now changed the text for better accuracy, saying "Next, we validated the phenotypes obtained from knockdown of the PP4$^{\text{SMK-1}}$ catalytic subunits by use of a loss-of-function allele of *pph-4.1*, *pph-4.1(tm1445)*. This allele lacks exons 3 and 4, which contain the enzyme's active site."

Line 139: "Consistent with the catalytic activity of PP4SMK-1 being required, overexpression of the catalytic dead form of PPH-4.1 led to lifespan shortening in daf-2 mutant animals". Phrase more carefully in the light of Fig. 2F, which shows that daf-2(-) worms overexpressing the R262L-mutant are still substantially long-lived (by inference; a wt control was not included in this experiment), although not as long-lived as daf-2(-) worms overexpressing wildtype PPH-4.1. This would mean that catalytic activity contributes, but is not absolutely required for daf-2 longevity, although it could be a dose effect as both overexpression lines still harbor endogenous pph-4.1 activity.

Our response: Expression of the R262L-mutant PPH-4.1 has been extremely difficult. This mutant likely behaves like a null; and since entire loss of *pph-4.1* leads to sterility, the generation of transgenic lines that would predominantly or even exclusively express the R262L-mutant is difficult if not impossible. In the end, it was essential for the generation of transgenic lines that the endogenous *pph-4.1* was still present and that expression of the R262L-mutant occurred at a rather low level. This means that the animals used in Figure 2f surely retained significant expression of PP4$^{\text{SMK-1}}$ complexes containing wild-type PPH-4.1 that would still fulfill some of PP4$^{\text{SMK-1}}$'s functions. We therefore do not find it surprising that R262L-mutant expression only partially suppresses the longevity of *daf-2* mutant animals and do not see it as an indication for PP4$^{\text{SMK-1}}$ to only partially rely on its catalytic activity for its longevity-promoting effects. Nevertheless, we now rephrased this paragraph, clarifying that we observe here only a partial suppression of *daf-2* longevity.

□ To address this possibility, include daf-2(e1370) animals not overexpressing any of the two constructs!

Our response: Unfortunately, we did not fully understand the reviewer's reasoning. We believe that including these animals in the lifespan analysis would not lead to valuable new insight, i.e. regarding the importance of the catalytic activity of PPH-4.1 for the promotion of longevity by DAF-16 under low IIS. In any regard, we believe that Figure 2f is well controlled, by comparing the effects of the *pph-4.1(R262L)* transgene to an otherwise identical *pph-4.1* wild-type transgene and by showing that the observed effects are *daf-16*-dependent.

Generally, to better assess the requirement of PPH-4.1 catalytic activity, expression of R262L and wt PPH-4.1 needs to be done in the pph-4.1 mutant background!

Our response: We agree that overexpression of the catalytic dead PPH-4.1 in the *pph-4.1* mutant background would be the cleaner experiment. However, as we already stated above, generation of such lines was not achievable for us. Since *pph-4.1* mutants are sterile and since the catalytic dead PPH-4.1 does not rescue this phenotype, we would have to work with balanced strains carrying extrachromosomal PPH-4.1-expressing transgene arrays. And already in wild-type background, R262L-mutant expressing transgenic lines were extremely difficult to obtain.

Fig. 2 and supplementary table 5: It seems as if the data in the tables (and by extension, the graphs) show pooled results from multiple experiments (Figure legend says n>100, but table contains e.g. n = 529, which would be rather unusual for a single experiment). This needs to be clarified. If the data is pooled, from how many trials? Why are worm numbers vastly different in some cases (e.g. 2A: n = 175-529)? What about censoring? In many tables, there are n < 100, contrary to what the figure legend suggests.

Our response: We apologize for the incorrect statement of n>100 in the figure legend. Indeed, a few lifespan curves are based on less than 100 worms. We have now removed the statement of n>100 from the figure legend and only refer to the worm numbers stated in the Supplementary Table (now Supp Table 4). Regarding the pooling of experiments: We actually did not pool experiments. This means that even the 529 worms for the [daf-2(e1370)III; eri-1(mg366)IV; control RNAi] condition in Figure 2a have actually been scored in one experiment. However, we agree that 529 worms were an unnecessary large number of animals for the particular experiment.

Fig. 2C: smk-1 RNAi should have been included in these particular experiments, to be really able to compare the combined knockdown of the two catalytic subunits to smk-1

Our response: We actually had included *smk-1* RNAi in this experiment but just not shown this data in Figure 2c. We have now added this data to the figure.

Fig. 2F: How many independent transgenic lines were examined? Generally, the field requires at least two independent lines.

<u>Our response:</u> For each transgenic line used in Figure 2f, we examined two independent transgenic lines, with consistent results. Only one set of lines is shown in Figure 2f. The results for the second set of lines we have now added as a new Supplementary Figure 2f.

> *Section PP4SMK-1 is required for DAF-16 to promote resistance to oxidative stress and UV irradiation but not to heat stress. Line 147: "Much of the DAF-16-driven longevity in daf-2 mutant animals can be attributed to improved stress responses". Include a reference for this statement.*

<u>Our response:</u> We now have added such reference to the manuscript: (Murphy et al., 2003)

> *Fig. 3 and supplementary table 5. Similar to Fig.2. In some tables it seems as if only one single experiment was conducted (e.g. Fig. 3D, n ~ 50!). Clarify whether this is one single experiment/pooled data from multiple experiments (n?)/representative experiment (n?). Again, why is n in some cases so different in some panels?*

<u>Our response:</u> The shown data refer to only a single representative experiment. However, all results were confirmed by a second independent experiment. Regarding the question of why n can be quite different between conditions: This sometimes is caused by our experimental design, e.g. we may include additional control conditions on each scanner of the lifespan machine or on each multi-well plate used, which results in more control animals.

> *Section PP4SMK-1 is required for a significant part of the DAF-16-mediated gene expression changes in low IIS mutants Line 172: "Using the resulting data, we first determined the genes differentially expressed by the reduced IIS in daf-2 mutant animals, identifying 1093 upregulated and 2054 downregulated genes (Fig. 4a)." How well do the authors' lists of up/downregulated genes overlap with published RNAseq/microarray studies comparing wt and daf-2 animals or daf-2 and daf-2; daf-16 animals? Comment on this, to validate your sequencing data.*

<u>Our response:</u> Here it is important to note that no perfect overlap between our and other published work can be expected, since there exist always some minor differences between growth conditions, the investigated stage of the animals, the exact genotypes or RNAi used, and the mRNA quantification methods used (e.g. microarray vs mRNA-seq, differences in library construction methods, etc). Nevertheless, conducting comparisons with prior work, we find our mRNA-seq data for wild-type versus *daf-2* or for *daf-2* versus *daf-2; daf-16* to be very consistent:

For wild-type vs *daf-2* we compared our lists of up- and downregulated genes to published mRNA-seq data by (Riedel et al., 2013). Even though this prior study used wild-type background in contrast to our *eri-1(mg366)* background, 55% of the activated and 52% of the repressed genes were confirmed by our new study.

For *daf-2* versus *daf-2; daf-16* we compared our lists of up- and downregulated genes to published microarray data by (Murphy et al., 2003) and mRNA-seq data by (Riedel et al., 2013). Even though (Murphy et al., 2003) used early microarray methods from more than 15 years ago and they used *daf-16(mu86)* instead of *daf-16* RNAi as well as wild-type instead of an *eri-1(mg366)* background, 45% of the genes activated by DAF-16 and 36% of the genes repressed by DAF-16 could be confirmed by our new study. Similarly for (Riedel et al., 2013), even though they used *daf-16(mgDf47)* instead of *daf-16* RNAi and wild-type instead of an *eri-1(mg366)* background, 58% of the genes activated by DAF-16 and 57% of the genes repressed by DAF-16 could be confirmed by our new study.

Taken together, the observed overlaps meet our expectations, they are all highly significant (as judged by hypergeometric tests), and thus we see them as support for the validity of our analyses.

Also, how is up/downregulation defined? Specify here and/or in Methods section!

Our response: Significantly up/downregulated genes were identified by use of the software Cuffdiff, using a FDR of 5%. We have now adjusted the respective methods section "Processing of mRNA-seq reads, differential gene expression analysis, and evaluation of gene list overlaps", to better clarify this.

What about targets of other transcription factors involved in daf-2 longevity (e.g. skn-1)?

Our response: To determine, if SMK-1 is also influencing gene expression changes conferred by other transcription factors that contribute to the longevity of *daf-2* animals, we now investigated overlaps of SMK-1-regualted genes with those regulated by two prominent examples of such transcription factors, SKN-1 (Ewald et al., 2014) and HLH-30 (Lin et al., 2018). It turns out that 11% of the genes that are activated by SKN-1 under low IIS are also activated by SMK-1, and 9% of the genes that are repressed by SKN-1 under low IIS are also repressed by SMK-1. The overlap for the activated genes is on the borderline to significance (p=0.009), while the overlap for repressed genes is not significant (p=0.834). For HLH-30 we found that 44% of the genes that are activated by HLH-30 under low IIS are also activated by SMK-1, and 57% of the genes that are repressed by HLH-30 under low IIS are also repressed by SMK-1. These overlaps are both highly significant ($p=1.274 \times 10^{-218}$ and $p<1 \times 10^{-300}$, respectively).

Taken together, SMK-1 affects expression not only of a subset of DAF-16 target genes but it seems to be involved in the target gene regulation of other transcription factors that contribute to *daf-2* longevity, too. Even though target gene expression by SKN-1 appears to only marginally be affected by absence of SMK-1, SMK-1 appears to substantially influence target gene expression by HLH-30. This impact of SMK-1 on other transcription factors downstream of *daf-2* will be interesting to explore in the future.

Fig. 4. D, e: The GO-term "Aging" is very superficial. The authors extensively described the stress resistance phenotypes of PP4-ko…look specifically for co-regulation of genes that could explain this phenotype as well!

Our response: We agree that the GO-term enrichment analysis of Figure 4d does not explain the observed role of SMK-1 in supporting some stress resistance phenotypes of *daf-2* animals (e.g. resistance to ox stress or UV stress) but not others (i.e. resistance to heat stress). However, it displays only the GO-terms with a score above our chosen significance cutoff of 1.3. If we extend this list to lower scores, the next term down the list is actually "redox homeostasis", with a score of 0.9. We argue that these proteins contribute to oxidative stress responses. In contrast, our analysis did not reveal any enrichment at all for terms related to heat stress resistance or protein homeostasis. While these observations don't provide a robust explanation for why SMK-1 shows specificity for certain stress responses, they are still consistent with the idea that SMK-1 influences expression of some stress response genes (e.g. conferring redox homeostasis or oxidative stress resistance) while not influencing others (i.e. conferring protein homoestasis or heat stress response). We have now extended Figure 4d to also include this "redox homeostasis" GO-term, but we mark it as being below significance cutoff.

Section PP4SMK-1 only mildly affects nuclear entry of DAF-16 and its binding to chromatin Fig. S3: Not clear how many animals were scored per strain and how many experiments were conducted. Provide representative images of the 5 different states distinguished during scoring.

Our response: We scored at least 100 worms per timepoint. This information has now been added to the respective figure legend. Further, we provide representative images of the different scored states in Supplementary Figure 5.

Section PP4SMK-1 promotes DAF-16-driven longevity and stress resistance by dephosphorylating SPT-5
Fig. 6b: Provide images of SPT-5 influencing expression of the sod-3 reporter

Our response: We now show such images in the new Supplementary Figure 8a.

Section PP4SMK-1 promotes DAF-16-driven longevity and stress resistance by dephosphorylating SPT-5
Line 301: "Overall, our data is consistent with SPT-5 being the relevant substrate, by which PP4SMK-1 promotes Pol II recruitment and transcriptional initiation at many DAF-16 target genes and thus fulfills its lifespan and stress resistance regulatory roles under low IIS" Correct, the data are consistent with this conclusion. To further substantiate this conclusion that PP4SMK-1 promotes longevity by dephosphorylating SPT-5, the authors need to show that expression of a constitutively dephosphorylated SPT-5 can overcome the lifespan-shortening effect of pp4-1/2 RNAi in daf-2 mutant animals. Moreover, the data do not provide convincing evidence for SPT-5 being a direct PP4SMK-1 substrate. This requires an in vitro phosphatase assay.

Our response: Regarding the expression of a constitutively dephosphorylated SPT-5: We attempted to generate such animals already twice, by ourselves and through a company. However, in both cases we unable to obtain animals that would express such transgene. Thus, we assume that expression of a constitutively dephosphorylated SPT-5 is toxic for the animals.

Regarding the question of whether SPT-5 is a direct target of $PP4^{SMK-1}$, we now have conducted an *in vitro* phosphatase assay. It shows that purified $PP4^{SMK-1}$ is able to dephosphorylate at least two of the four $PP4^{SMK-1}$-dependent phosphorylation sites in SPT-5. This data is presented in Supplementary Figure 9c, e. We conclude from this data that SPT-5 is indeed a direct substrate of $PP4^{SMK-1}$.

Dephosphorylation of SPT-5 by PP4SMK-1 promotes multiple transcriptional events, including Pol II recruitment, transcriptional initiation, and transcriptional termination Cf. comment above. The text (here and also in the discussion) as it is written implies that SPT-5 is a direct substrate. Perform an in vitro phosphatase assay!

Our response: As stated also in response to the previous comment, we now have conducted such *in vitro* phosphatase assay. It shows that purified $PP4^{SMK-1}$ is able to dephosphorylate at least two of the four $PP4^{SMK-1}$-dependent phosphorylation sites in SPT-5. This data can now be seen in Supplementary Figure 9c, e. We conclude that SPT-5 is indeed a direct substrate of $PP4^{SMK-1}$.

Discussion

Line 376: "This complex contains only some of the possible PP4 subunits, namely PPH-4.1/PPH-

4.2, providing the catalytic activity, as well as PPFR-2 and SMK-1, two regulatory subunits. Notably, we found also PAA-1 in this complex, normally a structural subunit of PP2A complexes11. It will be interesting to explore in follow-up studies, why this subunit is present in PP4SMK-1." This is all based on IP/mass spec studies, which, as indicated above, cannot distinguish whether proteins interacting with a bait protein do so as part of a common complex or of separate complexes. Answering this question would require reciprocal co-IP studies on all proteins in question. Perform these experiments or phrase more carefully!

Our response: We agree with the reviewer that we should phrase this more carefully. Since PPH-4.1/PPH-4.2, PPFR-2, and SMK-1 have been shown already in multiple species and by several labs to reside in the same complex, we don't think that we overstate anything in their regard. However, we agree that we only provide an indication but have not conclusively shown that PAA-1 is part of this $PP4^{SMK-1}$ complex. Alternatively, PAA-1 and SMK-1 could also form a separate complex that does not contain other PP4 subunits. We therefore removed our statements about PAA-1 from this paragraph.

Materials and Methods

This section lacks many important details that may unnecessarily complicate potential attempts to reproduce the data by other labs - The authors use strains from several labs. Where alleles obtained from other labs (CB, KW, AS, CF-strains) outcrossed to the author lab's N2-strain?

Our response: Yes, we 4 times outcrossed alleles obtained from other labs to the lab's N2 background.

- State precisely which RNAi-clones were from which library and which ones were generated in house

Our response: We now added this information to the methods section "RNAi by feeding".

- Also state in methods that generally, an RNAi-sensitive strain was used and knockdown was initiated in L1

Our response: We now added this information to the methods section "RNAi by feeding".

- How was the pph-4.1. promoter for the transgene defined?

Our response: Given that there is the gene Y75B8A.31 closely upstream of the *pph-4.1* locus, we decided to use a rather minimal promoter of the 600 bp immediately upstream of the *pph-4.1* START codon. We now added this information to the respective methods section.

- Specify the amounts of transgene/coinjection/filler DNA used for injection

Our response: We now added this information to the respective methods section.

- Stating that "large scale immunoprecipitation…" was performed as previously described is not sufficient. It should at least be stated whether worms lysed for IP/mass spec were also from a mixed stage population or synchronized and harvested as L4/adults etc

Our response: We provide now a more detailed description of our *C. elegans* immunoprecipitation experiments in the methods section, which also clarifies that the IP/mass spec was conducted from an asynchronously grown mixed-stage population.

- Methods for stress survival assays state that animals were transferred to tBOOH at day 2 of adulthood, but legend for figure 3 says day 1. Clarify!

Our response: We apologize for this inconsistency. The animals were transferred on day 2 of adulthood. We now corrected the legend of Figure 3 accordingly.

- How was transgene expression scored? Visually or by some image analysis method?

Our response: It was scored visually. We have now added this information to the respective methods section.

- Which concentration of FUDR was used?

Our response: We always used a concentration of 50 μM. We have now added this information to the respective methods sections and figure legends.

- Which lysis buffer was used in the PhosTag SDS-PAGE and how much total protein was loaded?

Our response: We apologize for this omission and have now added this information to the respective methods section.

- Instead of stating how many worms were bleached before mRNA isolation, it is more informative to mention how many L1s were plated and subsequently harvested for mRNA isolation

Our response: We rephrased this methods section, clarifying that it is eventually 100 worms which are harvested for mRNA isolation.

- Which RNA quality control steps were performed before library synthesis?

Our response: RNA quality was assessed by determining its RNA integrity number (RIN). We required all samples to have a RIN above 9.

- How many worms were grown for Chip-Seq?

Our response: We used approximately 800,000 worms per condition. We have now added this information also to the respective methods section.

- How precisely are up/downregulated genes defined?

Our response: As also stated above, significantly up/downregulated genes were identified by use of the software Cuffdiff, using a FDR of 5%. We have now adjusted the respective methods section "Processing of mRNA-seq reads, differential gene expression analysis, and evaluation of gene list overlaps", to better clarify this.

Reviewer #4:

The manuscript by Sen et al describes a mechanism through which SMK-1 may regulate the activity of DAF16/FOXO-dependent genes, which it appears to do through dephosphorylation of Spt5, a subunit of DSIF factor that in turn is involved in regulation of transcription initiation, Pol II pausing and elongation.

The work touches upon an important signaling pathway and appears to be quite novel and of general interest. The novelty of this study, in my opinion, is demonstration that a Pol II pausing factor that participates in transcription of all genes is a regulatory target for modulating only a specific pathway. The hypothesis proposed here could be quite compelling and has to do with PP4 complex dynamically regulating Spt5 phosphorylation levels, thereby maintaining Spt5 functionality. I am not entirely sure about the arguments made in support of this idea: they ether need to be clarified or the idea needs to be toned down.

Our response: We understand the reviewer's concern. Some of our mechanistic interpretations and resulting statements may have been too strong or unclear. We therefore have extensively revised those parts of the manuscript to improve clarity, to tone down potential overinterpretations, and to also better accommodate alternative mechanistic interpretations of our data and findings.

The methodology proposed and used is for the most part adequate, and tests are rigorous. I have a question about quality of ChIP-seq datasets. Based on what's presented, it is impossible to determine if these ChIP-seq datasets are good or not. At the very least, some quality metrics should be presented, plus browser shots of relevant genes should be shown to show signal to noise ratio. It is not even clear what the sequencing coverage was. ChIP-sequencing presentation is definitely a weak point of this manuscript and needs to be remedied, hopefully with tools and information that authors already should have at hand.

Our response: We now provide detailed statistics for all the ChIP-seq data, including the sequencing coverage, in Supplementary Table 5. As can be seen, the data should be of good quality, showing enough sequencing coverage (at least ~4-fold, mostly around ~10-fold) and low read replication (usually better than 75% unique reads in 10 Mio reads). For actual browser shots of relevant genes, we refer to the new Supplementary Figure 6 and Supplementary Figure 4e, the first of which has also been created to address the next comment below. In any regard, we believe that they illustrate a good signal-to-noise ratio in our ChIP data.

On a related note, the shift of ChIP-seq peaks shown on metagene plots is not convincing and needs to be demonstrated through independent experimentation or analysis. For example, are there individual genes where this shift can be seen?

Our response: In our original manuscript, we suggested based on Figures 5b and 5d that there is two phenotypes arising from knockdown of $PP4^{SMK-1}$: 1) An overall decrease in RNA Pol II binding/recruitment to TSS regions, specifically at genes that are co-activated by DAF-16 and $PP4^{SMK-1}$ under low IIS. 2) A moderate shift of RNA Pol II distribution from the pause site in the gene body to the promoter region (the region upstream of the TSS), which may indicate a defect in transcriptional initiation. Also, this phenotype specifically occurs at genes that are co-activated by DAF-16 and $PP4^{SMK-1}$ under low IIS.

We have now carefully reinvestigated these phenotypes on a gene-by-gene level and conclude that the first phenotype, namely decreased RNA Pol II binding to TSS regions, is robust and can be observed at many genes. Consistently, quantification of this decrease in Figure 5b shows it to be statistically significant. We now provide views from the UCSC genome browser for examples of genes where this decrease occurs (see in particular the left side of Supplementary Figure 6; genes *dod-3* and *col-10*).

Regarding the second phenotype, we have to conclude that the shift of RNA Pol II distribution from the pause site into the promoter region is a more subtle phenotype. As can be seen in Figure 5b, it does not resemble a full relocalization of RNA Pol II but only a shift of its distribution's summit. Furthermore, this shift amounts to ~150 bp, which is close to the resolution limit of our ChIP-seq experiment. Thus, maybe not so surprisingly, looking at the single gene level we found it much more difficult to identify individual genes where this exact shift can be seen. The right side of Supplementary Figure 6 shows now some of the better examples (the genes *aagr-4* and *sqt-3*) where Pol II seems to shift into the 5' direction, when *smk-1* has been knocked down. Ultimately though, the shift becomes predominantly visible only by the meta-gene analysis of Figure 5b, combining 150 genes, which helps to consolidate the phenotype and reduces the noise.

Taken together, the reviewer touches upon a relevant point, namely that while loss of $PP4^{SMK-1}$ causes clearly a robust defect in RNA Pol II recruitment to TSS regions, it's impact on RNA Pol II moving from promoter regions into the gene body and thus to undergo initiation is less obvious. However, the fact that *smk-1* RNAi leads to reduction of Ser5-phosphorylated RNA Pol II at the TSS (Figure 5d) provides some additional support for an initiation defect and the physiological relevance of this shift. Nevertheless, we now removed some of the emphasis from $PP4^{SMK-1}$ influencing the actual step of transcriptional initiation throughout the manuscript, leaving more room for alternative interpretations of our data.

Another issue notable even without having access to additional data is normalization between different ChIP seq samples such as for figure 7. I am not sure it was adequately done as currently described, and certainly requires additional explanation.

Our response: We apologize for not sufficiently explaining how the Chip-seq data in Figures 5b-d or 7c,d were normalized. We have now added a clear explanation of how normalization was conducted and how the eventual "fold enrichment over input" values were obtained to the methods section "Processing of ChIP-seq reads and metagene analysis".

1. Quote. "This phosphatase complex binds and dephosphorylates the transcription elongation factor SPT-5, and in turn this supports efficient RNA polymerase II (Pol II) binding to promoter regions, transcriptional initiation, and eventually transcriptional termination and the release of Pol II and SPT-5 from transcriptional end sites (TESs) under low IIS." If, as stated in Reference 34, SPT5 is phosphorylated to release paused Pol II into elongation, then what is the above statement (that dephosphorylation promotes these steps rather than inhibits them) based on? There seems to be a contradiction here unless I am misunderstanding the authors' explanation.

Our response: Even after careful consideration of the reviewer's comment, we do not really see here a contradiction. It is true that phosphorylation of SPT-5 by P-TEFb is needed, to release paused RNA Pol II into elongation. But this should not interfere with dephosphorylation of SPT-5 promoting other stages of transcription, i.e. Pol II recruitment, transcriptional initiation, or transcriptional termination. The statement that is quoted by the reviewer is then based on the phenotypes that we observe for RNA Pol II behavior upon knockdown of $PP4^{SMK-1}$, mostly shown in Figures 5 and 7. Basically, we think that the phosphorylation state of SPT-5 has to cycle throughout the different stages of transcription.

If PP4 complex dephosphorylates SPT5, this should in theory lead to inactivation of transcription. How come depletion of one, as stated in the abstract, phenocopies rather than antagonizes depletion of the other? I understand that a mechanism is possible wherein timely dephosphorylation of Spt5 is needed to get the turnover of SPT5 going. Is this is true, then Pol II should get stuck somewhere at the ends of genes, not at their promoter regions, no? As written, I fail to see a connection between the argument and the experimental outcome of PP4 depletion on a specific step of Pol II transcription. This may be a presentation issue, not a problem with experiments.

Our response: We do not think that knockdown of $PP4^{SMK-1}$ causes RNA Pol II to substantially get stuck at any particular moment during transcription. First, *smk-1* RNAi will actually cause substantially less RNA Pol II to be recruited to the promoter region and thereby cause less RNA Pol II to be present throughout the gene. Second, *smk-1* RNAi will impair promoter clearance/transcriptional initiation. But as also already stated in a comment above, this phenotype is moderate, hard to see on the single-gene-level, and thus surely does not mean that RNA Pol II gets fully stuck in the promoter region. Finally, we do see an impact of knocking down $PP4^{SMK-1}$ on the release of RNA Pol II from DNA during termination. However, this is a delayed and not a fully blocked release. Thus, again RNA Pol II does not get stuck here. We now made some adjustments to the manuscript, hoping to provide better clarity on this matter.

2. As I mentioned above ChIP-seq experiments need to be presented better. This is one of the most difficult molecular biology methods to get right and showing a metagene squiggle is not enough, and example genes should be shown at least in the supplement. Also, the tiny shift shown on these metagenes is not convincing because the shift distance is well within technical variations of DNA fragmentation and therefore must be presented better. Was the ChIP done in single-end or paired end format? If it is single end for a shorter sequencing run, then it is practically a nonstarter for this type of resolution even with opposite strand tag peak center approximation. Also, it may be my fault, but I may have missed the antibody that was used for ChIP of total Pol II (not S5P).

Our response: Regarding example genes: We now show ChIP-seq tracks for some example genes in the new Supplementary Figures 6 and 4e.

Regarding the shifts in the meta-gene analyses: As stated also in the methods section, we sequenced our ChIP-seq libraries on Illumina HiSeq 2000 sequencers, generating single end 50 bp reads. These reads were then mapped to the *C. elegans* genome and extended by an additional 150 bp in their 5'-direction, to eventually resemble the approximate size and sequences of the fragments in our ChIP-seq libraries. While this approach is not as accurate as paired-end sequencing approaches, it is the most commonly used; and in most cases it is considered sufficient for interpretation of ChIP-seq libraries – even by major consortia like ModEncode (Niu et al., 2011). In Figure 5, we describe peak shifts in meta-gene plots in the magnitude of approximately 150 bp. We agree with the reviewer that such shifts would be hard to claim on a single gene level. However, the metagene plots of Figure 5 are based on at least 150 genes, which immensely helps to consolidate phenotypes and eliminate effects of stochastic experimental noise. Thus, we believe that the

shifts which we describe in Figures 5 and 7, even though they are not the most robust phenotypes, are still noteworthy and likely to be true.

Regarding the antibody to immunoprecipitate total RNA Pol II: We used the mouse monoclonal antibody 8WG16 from BioLegend (Cat# 920102).

> *3. Do authors see docking of Pol II described by Baugh R et al, in 2013 and, if so, are there any changes on these genes?*

Our response: To properly and reliably identify genes that are in a "docked" state, as described by (Maxwell et al., 2014), one would need to conduct not only RNA Pol II ChIP-seq but also GRO-seq under the given conditions, i.e. in *daf-2(e1370ts)* animals. Unfortunately, we lack this GRO-seq data for our *daf-2(e1370ts)* animals. Nevertheless, we do not think that the genes co-activated by DAF-16 and SMK-1 are particularly enriched for genes with such truly "docked" RNA Pol II. First, when we look for overlap of the more than 775 genes with "docked" Pol II published by (Maxwell et al., 2014) with our 150 genes co-activated by DAF-16 and SMK-1, we find a mere overlap of 2 genes, which is insignificant ($p>0.05$, hypergeometric test). Second, the genes with "docked" Pol II published by (Maxwell et al., 2014) were found to lack TATA boxes; but we show in Supplementary Table 8 that the genes co-activated by DAF-16 and SMK-1 are significantly enriched for TATA boxes in their promoter regions. Finally, according to (Maxwell et al., 2014), genes with "docked" Pol II preferentially promote growth but not the resistance to stress; in contrast, Figures 2-4 argue that the genes co-activated by DAF-16 and SMK-1 promote stress resistance. Taken together, we don't think that genes with truly "docked" RNA Pol II play a role in the co-regulation of gene expression by DAF-16 and SMK-1 and thus the subject of our manuscript.

> *4. I do not fully understand the section where PP4 complex is required for DAF-16 to promote resistance to one type of stress, but not the other (heat shock). This is an interesting observation, but it is not placed well in the context of the manuscript flow. The way I interpret this section is that DAF-16 is not at all the point of this manuscript. Rather, the point of this manuscript is PP4 complex' effect on what is essentially a general factor (SPT5) leading, surprisingly, to defects in specific genes rather than general effect. In other words, this result is certainly worth presenting, but it needs to be incorporated into the flow of the manuscript better.*

Our response: We agree with the reviewer and now tried our best to adjusted the manuscript accordingly.

> *5. Line 198 Supplement Fig 3b, c should be in the main figure, not in supplement. This is an important piece of data.*

Our response: While we agree with the reviewer that this data is relevant for the story-line and reasoning of the manuscript, we prefer to keep this data in the supplement, for the following reasons: 1) All the information provided in Supplementary Figure 4 (formerly Supp Fig 3) forms one thematically connected set of experiments, in which we test whether the events of nuclear entry/exit and the binding of DAF-16 to target genes are affected by $PP4^{SMK-1}$. Further, we deem the data of Supplementary Figure 4d (formerly Supp Fig 3d) equally important to Supp Fig 4b, c (formerly Supp Fig 3(b, c)). As a consequence, we prefer to not divide Supplementary Figure 4 (formerly Supp Fig 3) but to keep it together as one figure. 2) The result of Supplementary Figure 4b (formerly Supp Fig 3b) is not entirely novel. The fact that SMK-1 does not majorly influence nuclear entry of DAF-16 had already been shown in prior work (Wolff et al., 2006). The only novelty was that we investigated this matter in more detail, by not only looking at endpoints of

translocation but the kinetics. 3) The data shown in Supplementary Figures 4b, c (formerly Supp Fig 3(b, c)) is mostly negative.

In summary, we find the data of Supplementary Figures 4b, c (formerly Supp Fig 3(b, c)) more suitable for presentation in the Supplement, together with the data from Supplementary Figure 4d (formerly Supp Fig 3d) and the diagram of Supplementary Figure 4a (formerly Supp Fig 3a). However, if the editor prefers, we could certainly upgrade Supplementary Figure 4 (formerly Supp Fig 3) to become a new main figure.

6. The last Results section "Dephosphorylation of SPT5" appears to be a standalone discussion with a mini introduction to boot. This verbiage may be hiding a crucial, and maybe the most important point of the manuscript, namely, the mechanistic consequence of SPT5 dephosphorylation on Pol II transcription. Authors suggest that this leads to changes on all steps of transcription. It is more likely that only one step is perturbed directly, and the rest are secondary, not direct, effects. In order for me (and reader) to see the data and arguments, this section needs to be shortened and clarified. There are strong conclusions made here, but the evidence is hidden behind too much wording and again squiggles of ChIP-seq traces.

<u>Our response:</u> We have now adjusted the last results section to be more concise and clearer for the reader, and we now accommodate other possible mechanistic explanations of the observed phenotypes.

7. On a related technical note, how was normalization made between different specimens In 7a, b, etc. ChIP normalization is a difficult task because differences in background will completely skew the data.

<u>Our response:</u> For each of our ChIP-seq experiments we used a highly standardized experimental protocol, we processed all the samples in parallel, and we normalized each sample to its corresponding input material, to assure best possible data quality and comparability between conditions. Thus, in Figures 5b-d and 7c,d as well as Supplementary Figures 4d (formerly Supp Fig 3d) and 10 (formerly Supp Fig 7) an input-normalized ChIP IP signal is plotted. This means that we first determined normalized read densities for input and IP samples in 60 bp bins across the displayed regions. Then we normalized the value for the IP bin by dividing it by the value for the corresponding input bin. This results in the "fold enrichment over input" which is plotted on the y-axes in all these figures. We now also explain our method of input-normalization in more detail in the methods section.

Minor issues.

1. In the abstract, loss of Spt5 is mentioned. Isn't this factor essential so its loss is hard to interpret. Did authors mean partial depletion?

<u>Our response:</u> Throughout the manuscript we occasionally use the term "loss" to refer not only to null mutations but also to partial loss-of-function situations, e.g. the consequences of a knockdown. However, we agree with the reviewer that especially in the abstract better accuracy may be desired. Thus, we exchanged here now "loss" for "post-developmental knockdown".

2. "Phosphorylation of SPT-5 is crucial for efficient transcriptional elongation but otherwise absent during other stages of transcription. We propose that this absence is important and assured by PP4SMK-1. "The importance of absence is a very confusing construct that makes little sense

and muddles understanding of the abstract and the manuscript. How can the absence be required for transcription initiation, and termination?

Our response: We have rephrased those parts of the abstract for better clarity. They now read "Phosphorylation of SPT-5 is crucial for efficient transcriptional elongation but the protein is found non-phosphorylated during other stages of transcription. We propose that maintenance of this non-phosphorylated pool of SPT-5 is important and assured by PP4SMK-1. Although it does not influence all genes in the genome, we see it required for efficient Pol II recruitment, and to facilitate transcriptional initiation and transcriptional termination, at genes whose expression may particularly depend on transcriptional initiation as a rate-limiting step."

3. In the abstract, a statement is made of genes that are strongly transcription initiation dependent. This statement makes no sense because all genes are absolutely and unequivocally transcription initiation dependent. Genes may have stronger or weaker promoters though, for example.

Our response: We agree that expression of all genes is transcriptional initiation dependent. However, the degree to which transcriptional initiation is rate-limiting for gene expression can differ between genes. We now have adjusted the sentence accordingly to read "Although it does not influence all genes in the genome, we see it required for efficient Pol II recruitment, and to facilitate transcriptional initiation and transcriptional termination, at genes whose expression may particularly depend on transcriptional initiation as a rate-limiting step."

4. What does this mean: "significant part of DAF-16 mediated gene expression changes"?

Our response: We now rephrased this expression to say "a subset of the DAF-16-mediated gene expression changes", hoping that this clarifies its meaning.

5. The statement that PP4 confers its phenotype by" licensing the transcriptional regulation of a subset" of genes… appears to carry no useful information and essentially reads as if it regulates a subset of genes by regulating a subset of genes.

Our response: We now rephrased this sentence to say "Taken together, $PP4^{SMK-1}$ is required for DAF-16 to successfully regulate many of its aging-related target genes."

6. In Figure 4A, if I am understanding it correctly, an argument is made about compensatory restoration of gene expression. This is impressive, but what should be clarified is how many genes (what %) restored is the threshold for a reader to get excited. I thought that 18.7% is a low number, but maybe not.

Our response: We investigated this matter extensively and had to conclude that it is very difficult to calculate an actual threshold value, beyond which such restoration of gene expression should be considered significant. Thus, the results must be viewed as descriptive and qualitative rather than quantitative results. Nevertheless, when we compare our results here to previously published analyses of the same kind, we can at least say the following: We previously determined such restoration rates for the loss of two other factors that are required for DAF-16 to express many of its target genes and to promote the longevity of *daf-2* animals, namely for the chromatin remodeling complex SWI/SNF (Riedel et al., 2013) and for the transcription factor HLH-30 (Lin et al., 2018). At least for the genes activated in *daf-2* mutants, *smk-1* RNAi leads to a better restoration rate (40.7%) compared to loss of *hlh-30* (31.2%) and it performs similarly to a

swsn-1 LOF mutant (42%). Only for genes repressed in *daf-2* mutants, it provides a worse restoration, namely 18.7% vs 54.6% and 32%, respectively.

From these comparisons we conclude that at least the transcription-activatory events under low IIS majorly depend on $PP4^{SMK-1}$.

References:

Adelman, K., and Lis, J.T. (2012). Promoter-proximal pausing of RNA polymerase II: emerging roles in metazoans. Nat. Rev. Genet. *13*, 720–731.

Chowdhury, D., Xu, X., Zhong, X., Ahmed, F., Zhong, J., Liao, J., Dykxhoorn, D.M., Weinstock, D.M., Pfeifer, G.P., and Lieberman, J. (2008). A PP4-Phosphatase Complex Dephosphorylates γ-H2AX Generated during DNA Replication. Mol. Cell *31*, 33–46.

Core, L.J., Waterfall, J.J., and Lis, J.T. (2008). Nascent RNA sequencing reveals widespread pausing and divergent initiation at human promoters. Science (80-.). *322*, 1845–1848.

Diamant, G., Bahat, A., Dikstein, R., Dvir, A., Conaway, J.W., Conaway, R.C., Liu, X., Bushnell, D.A., Silva, D.A., Huang, X., et al. (2016). The elongation factor Spt5 facilitates transcription initiation for rapid induction of inflammatory-response genes. Nat. Commun. *7*, 11547.

Ewald, C.Y., Landis, J.N., Abate, J.P., Murphy, C.T., and Blackwell, T.K. (2014). Dauer-independent insulin/IGF-1-signalling implicates collagen remodelling in longevity. Nature *519*, 97–101.

Garriga, J., Segura, E., Mayol, X., Grubmeyer, C., and Graña, X. (1996). Phosphorylation site specificity of the CDC2-related kinase PITALRE. Biochem. J. *320 (Pt 3*, 983–989.

Gingras, A.-C., Caballero, M., Zarske, M., Sanchez, A., Hazbun, T.R., Fields, S., Sonenberg, N., Hafen, E., Raught, B., and Aebersold, R. (2005). A novel, evolutionarily conserved protein phosphatase complex involved in cisplatin sensitivity. Mol. Cell. Proteomics *4*, 1725–1740.

Hastie, C.J., Carnegie, G.K., Morrice, N., and Cohen, P.T. (2000). A novel 50 kDa protein forms complexes with protein phosphatase 4 and is located at centrosomal microtubule organizing centres. Biochem. J. *347 Pt 3*, 845–855.

Ivanov, D., Kwak, Y.T., Guo, J., and Gaynor, R.B. (2000). Domains in the SPT5 protein that modulate its transcriptional regulatory properties. Mol. Cell. Biol. *20*, 2970–2983.

Lee, D.-H., Pan, Y., Kanner, S., Sung, P., Borowiec, J.A., and Chowdhury, D. (2010). A PP4 phosphatase complex dephosphorylates RPA2 to facilitate DNA repair via homologous recombination. Nat. Struct. Mol. Biol. *17*, 365–372.

Lin, X.-X., Sen, I., Janssens, G.E., Zhou, X., Fonslow, B.R., Edgar, D., Stroustrup, N., Swoboda, P., Yates, J.R., Ruvkun, G., et al. (2018). DAF-16/FOXO and HLH-30/TFEB function as combinatorial transcription factors to promote stress resistance and longevity. Nat. Commun. *9*, 4400.

Maxwell, C.S., Kruesi, W.S., Core, L.J., Kurhanewicz, N., Waters, C.T., Lewarch, C.L., Antoshechkin, I., Lis, J.T., Meyer, B.J., and Baugh, L.R. (2014). Pol II docking and pausing at growth and stress genes in C. elegans. Cell Rep. *6*, 455–466.

Murphy, C.T., McCarroll, S.A., Bargmann, C.I., Fraser, A., Kamath, R.S., Ahringer, J., Li, H., and Kenyon, C. (2003). Genes that act downstream of DAF-16 to influence the lifespan of *Caenorhabditis*

elegans. Nature *424*, 277–283.

Neil, H., Malabat, C., D'Aubenton-Carafa, Y., Xu, Z., Steinmetz, L.M., and Jacquier, A. (2009). Widespread bidirectional promoters are the major source of cryptic transcripts in yeast. Nature *457*, 1038–1042.

Niu, W., Lu, Z.J., Zhong, M., Sarov, M., Murray, J.I., Brdlik, C.M., Janette, J., Chen, C., Alves, P., Preston, E., et al. (2011). Diverse transcription factor binding features revealed by genome-wide ChIP-seq in *C. elegans*. Genome Res. *21*, 245–254.

Parua, P.K., Booth, G.T., Sansó, M., Benjamin, B., Tanny, J.C., Lis, J.T., and Fisher, R.P. (2018). A Cdk9-PP1 switch regulates the elongation-termination transition of RNA polymerase II. Nature *558*, 460–464.

Riedel, C.G., Dowen, R.H., Lourenco, G.F., Kirienko, N. V, Heimbucher, T., West, J. a, Bowman, S.K., Kingston, R.E., Dillin, A., Asara, J.M., et al. (2013). DAF-16 employs the chromatin remodeller SWI/SNF to promote stress resistance and longevity. Nat. Cell Biol. *15*, 491–501.

Shen, L., Shao, N., Liu, X., and Nestler, E. (2014). ngs.plot: Quick mining and visualization of next-generation sequencing data by integrating genomic databases. BMC Genomics *15*, 284.

Shetty, A., Kallgren, S.P., Demel, C., Maier, K.C., Spatt, D., Alver, B.H., Cramer, P., Park, P.J., and Winston, F. (2017). Spt5 Plays Vital Roles in the Control of Sense and Antisense Transcription Elongation. Mol. Cell *66*, 77-88.e5.

Tabb, D.L., McDonald, W.H., and Yates, J.R. (2002). DTASelect and Contrast: tools for assembling and comparing protein identifications from shotgun proteomics. J. Proteome Res. *1*, 21–26.

Wolff, S., Ma, H., Burch, D., Maciel, G.A., Hunter, T., and Dillin, A. (2006). SMK-1, an essential regulator of DAF-16-mediated longevity. Cell *124*, 1039–1053.

Yamada, T., Yamaguchi, Y., Inukai, N., Okamoto, S., Mura, T., and Handa, H. (2006). P-TEFb-mediated phosphorylation of hSpt5 C-terminal repeats is critical for processive transcription elongation. Mol. Cell *21*, 227–237.

Reviewers' comments:

Reviewer #1 (Remarks to the Author):

The authors have somewhat adequately addressed the criticism raised by this reviewer.

Reviewer #2 (Remarks to the Author):

This is a revised version of a manuscript I reviewed previously, exploring how SMK-1 promotes longevity and stress resistance in C. elegans. The new data strengthen the main conclusion, i.e., that SMK-1 functions as a part of a PP4 complex to modulate transcriptional output of DAF-16, in part by regulating the phosphorylation state of the conserved transcription elongation factor SPT-5. To address my concerns the authors added data showing that 1) C. elegans-derived PP4 catalytic and regulatory subunits (including SMK-1) form an active phosphatase complex when expressed in yeast, which can dephosphorylate phosphopeptides containing some of the SPT-5 target sites they previously identified; and 2) that combined effects of mutating or depleting SMK-1 and PP4 catalytic subunits are less than additive with respect to longevity and stress resistance, suggesting that they function in the same pathway. Together, the findings reported here constitute an important addition to our understanding of the DAF-16 pathway and the role(s) of SPT-5 in controlling gene expression. However, I still have reservations regarding interpretations of data, and objections to misstatements of what is known from previous studies. I strongly urge the authors to tone down some of their conclusions and refrain from excessive speculation regarding the mechanism by which SMK-1/PP4 is regulating transcription through SPT-5.

Below I reiterate my remaining, specific concerns:

1. As I pointed out in my initial review, a requirement that the SPT-5 CTD be hypo- or de-phosphorylated in order to engage with Pol II upstream of the pause site has never been demonstrated, and can only be inferred from effects on pausing and elongation of P-TEFb (which has other substrates), or postulated by analogy with the Pol II CTD. As potential (albeit also circumstantial) evidence against this model I can cite 1) the ability of DSIF, pre-treated with P-TEFb, to support efficient elongation in a nuclear extract system (ref. 39); and 2) the lack of any discernible phenotype in fission yeast strains with a phosphomimetic mutant variant ("T1E") as their only form of Spt5 (Schneider et al., MCB 30: 2353-64, 2010). The fission yeast ChIP-seq study (Parua et al., 2018) cited as evidence in the rebuttal also did not show this; total Spt5 and phospho-Spt5 profiles look quite similar at 5'-ends of genes. To be clear, I am not arguing that any of these studies refutes this model, or that the model is wrong, but rather that the extant literature does not prove it, and that the authors' contention that "it would make little sense for SPT5 to…arrive at the pause site with a phosphorylated CTD" is not really an argument one way or the other.

2. The effects of manipulating PP4 function (by smk-1 RNAi) on Pol II distribution are complex, and don't readily fit with known or suspected roles of SPT-5 or SPT-5-dephosphorylating enzymes. (That should not be taken as a criticism of the present study, but rather as reflective of gaps in our understanding of SPT-5.) In presenting a highly detailed molecular model (Fig. 7a), however, in which SPT-5 is directly promoting recruitment of Pol II (step 1) and already bound to Pol II at initiation (step 2), I'm afraid the authors are only adding to the confusion. Most prior work places DSIF recruitment downstream of Pol II recruitment and initiation (consistent with its role as an elongation factor), dependent on displacement of initiation factors such as TFIIE, which binds in mutually exclusive fashion to the same region of Pol II as Spt5 does (e.g. ref. 36, which is supported by many other studies). The idea that SPT5 might influence Pol II recruitment has more limited support, and the one cited study that explicitly suggested this (ref. 35) seemed to invoke a feedback-based mechanism, in which post-initiation recruitment of DSIF led to chromatin modifications that stabilized TFIID

association—a nuance omitted from the simple cartoon in Fig. 7.

3. This is a minor point, but in the new measurements of phosphatase activity of PP4 complexes towards SPT-5-derived phosphopeptides (Supplementary Fig. 9), there was one such peptide, containing phospho-S671, which could not be dephosphorylated under any of the conditions tested, leaving open the trivial possibility that it is simply not capable of serving as a substrate. Have the authors tried any commercial, non-specific phosphatase (e.g. lambda) on this peptide?

4. Another minor point: the aforementioned reference to Parua et al. is duplicated (refs. 37 and 38).

Reviewer #3 (Remarks to the Author):

In their revised manuscript entitled "DAF-16/FOXO requires Protein Phosphatase 4 to initiate the transcription of stress resistance and longevity promoting genes", Sen et al. satisfactorily addressed all my previous concerns by rephrasing certain statements, including key experimental information that was missing previously, and in some cases, by providing new data. In fact, I applaud the authors for their comprehensive and precise answers/additional explanations. Importantly, the conclusions from the first version of the manuscript remain valid. As indicated previously, the presented mechanistic insights into how the known C. elegans longevity regulator SMK-1 actually exerts its longevity-promoting functions clearly are of interest to the aging field. Accordingly, I now recommend publication in Nature Communications and only have minor comments/suggestions for further improvement:

- Supplementary figure 9d: the experiment shown is suitable to further support the conclusion of SMK-1 being a PP4-subunit and experimental details are well-described in the methods section; however, to facilitate "reading" this figure, align the label "SMK-1-HA…" horizontally, and not tilted.

- Previous comment on Fig.4a/overlap of up/downregulated gene lists with previous studies: consider including some of this information as a supplementary table or at least indicate that you checked the overlap with other similar studies by a "data not shown" statement. The apparently highly significant overlap with similar studies is indeed encouraging, and my gut feeling is that the reasons cited by the authors (slight differences in culture conditions, genetic background etc) can adequately explain the discrepancies with other studies.

Reviewer #4 (Remarks to the Author):

The manuscript is substantially improved.

For ChIP-seq, the issue of Pol II recruitment is solved. The issue of Pol II ChIP signal lateral shift is not. The validated results on Pol II recruitment are sufficient to make a point and the "shift" does not add anything to understanding the mechanism of transcription blockage beyond speculation. To get to the mechanisms requires additional experimentation beyond the scope of this paper. Gro/Pro-seq will not be helpful because the methods are blind to initiating complexes without pre-existing nascent RNA. Anything having to do with lateral shift of ChIP seq peaks should be either deleted completely or moved to discussion as very speculative at best.

Another remaining issue is that introduction into and explanation of DSIF does not occur until the very last section of Results and, when it does, really does not fit in the manuscript as written. Even the title of this Results subsection is rambling. This section needs to be way shortened to focus on actual results instead of introduction and discussion.

Discussion section is too long and is strongly suggested to be condensed down to the most essential points.

The text may need language editing throughout. The usage of commas appears to be arbitrary. Almost all instances of “eventually” should be deleted. Many “also” words are misplaced within respective sentences.

Line 344. “SPT-5 as the relevant substrate”. “The” is too strong a statement that implies an exhaustive/saturating screen, which is not what’s been done. “A relevant substrate” appears to be more appropriate.

Line 537-538. Where are therapeutic interventions coming from? What mechanism would be an interesting (?) therapeutic target, whatever that is. The entire last sentence carries no information and should be deleted.

Minor points.
Line 271 – 272. Transcription initiation includes Pol II recruitment, so the sentence makes no sense as written.
Line 330 “letting us disregard … as relevant substrates”. This reads ambiguous: why disregard something that is relevant? Perhaps “exclude” is what was meant.
Line 355. “Washed it into assay buffer” makes no sense.
Line 180-181. Can be deleted.
Line 203. “Successfully regulated”. How does one estimate success of regulation? Maybe “properly regulated” was meant.

Response to the editor's and the reviewers' comments regarding our revised manuscript:

We would like to thank the editor and reviewers for their feedback regarding our revised manuscript. We were pleased to read that reviewers #1 and #3 were satisfied with the revisions we made. The last minor comments by reviewer #3 have now been addressed. Further, we fully acknowledge the remaining concerns by reviewers #2 and #4 and tried our very best to alleviate them – mostly by adjusting the manuscript as described below.

Comments by the reviewers:

Reviewer #1:

> *The authors have somewhat adequately addressed the criticism raised by this reviewer.*

Our response: We are pleased that our revisions resolved the reviewer's concerns.

Reviewer #2:

> *This is a revised version of a manuscript I reviewed previously, exploring how SMK-1 promotes longevity and stress resistance in C. elegans. The new data strengthen the main conclusion, i.e., that SMK-1 functions as a part of a PP4 complex to modulate transcriptional output of DAF-16, in part by regulating the phosphorylation state of the conserved transcription elongation factor SPT-5. To address my concerns the authors added data showing that 1) C. elegans-derived PP4 catalytic and regulatory subunits (including SMK-1) form an active phosphatase complex when expressed in yeast, which can dephosphorylate phosphopeptides containing some of the SPT-5 target sites they previously identified; and 2) that combined effects of mutating or depleting SMK-1 and PP4 catalytic subunits are less than additive with respect to longevity and stress resistance, suggesting that they function in the same pathway. Together, the findings reported here constitute an important addition to our understanding of the DAF-16 pathway and the role(s) of SPT-5 in controlling gene expression. However, I still have reservations regarding interpretations of data, and objections to misstatements of what is known from previous studies. I strongly urge the authors to tone down some of their conclusions and refrain from excessive speculation regarding the mechanism by which SMK-1/PP4 is regulating transcription through SPT-5.*

Our response: We would like to thank the reviewer for the new feedback, and we are pleased to read that our new experimental data improved the manuscript and addressed some of the reviewer's comments. Further, we apologize that in the reviewer's opinion we didn't sufficiently tone down and adjust our interpretations of the data during the revision. We understand the reviewer's concern and are fully determined to adjust the manuscript, so that the concerns are alleviated.

Below I reiterate my remaining, specific concerns:

1. *As I pointed out in my initial review, a requirement that the SPT-5 CTD be hypo- or de-phosphorylated in order to engage with Pol II upstream of the pause site has never been demonstrated, and can only be inferred from effects on pausing and elongation of P-TEFb (which has other substrates), or postulated by analogy with the Pol II CTD. As potential (albeit also circumstantial) evidence against this model I can cite 1) the ability of DSIF, pre-treated with P-TEFb, to support efficient elongation in a nuclear extract system (ref. 39); and 2) the lack of any discernible phenotype in fission yeast strains with a phosphomimetic mutant variant ("T1E") as their only form of Spt5 (Schneider et al., MCB 30: 2353-64, 2010). The fission yeast ChIP-seq study (Parua et al., 2018) cited as evidence in the rebuttal also did not show this; total Spt5 and phospho-Spt5 profiles look quite similar at 5'-ends of genes. To be clear, I am not arguing that any of these studies refutes this model, or that the model is wrong, but rather that the extant literature does not prove it, and that the authors' contention that "it would make little sense for SPT5 to…arrive at the pause site with a phosphorylated CTD" is not really an argument one way or the other.*

<u>Our response:</u> We thank the reviewer for these comments. We had proposed that the SPT-5 CTD needs to be hypo- or dephosphorylated to engage with Pol II upstream of the pause site, because we felt that this model was most compatible with our data. But indeed, we do not provide proof for this model/hypothesis. Other explanations cannot be excluded. It also is correct that the two pieces of evidence mentioned here by the reviewer speak against our hypothesis/model. To therefore address the reviewer's concern and to avoid any overinterpretation of our data, we have now substantially rewritten the manuscript and revised our model in Figure 7. We no longer make any claims about SPT-5 arriving hypo- or de-phosphorylated at the TSS region. In fact, we now refrain from making any claims at all about the phosphorylation status of $PP4^{SMK-1}$-targeted sites on SPT-5 during specific stages of transcription.

2. *The effects of manipulating PP4 function (by smk-1 RNAi) on Pol II distribution are complex, and don't readily fit with known or suspected roles of SPT-5 or SPT-5-dephosphorylating enzymes. (That should not be taken as a criticism of the present study, but rather as reflective of gaps in our understanding of SPT-5.) In presenting a highly detailed molecular model (Fig. 7a), however, in which SPT-5 is directly promoting recruitment of Pol II (step 1) and already bound to Pol II at initiation (step 2), I'm afraid the authors are only adding to the confusion. Most prior work places DSIF recruitment downstream of Pol II recruitment and initiation (consistent with its role as an elongation factor), dependent on displacement of initiation factors such as TFIIE, which binds in mutually exclusive fashion to the same region of Pol II as Spt5 does (e.g. ref. 36, which is supported by many other studies). The idea that SPT5 might influence Pol II recruitment has more limited support, and the one cited study that explicitly suggested this (ref. 35) seemed to invoke a feedback-based mechanism, in which post-initiation recruitment of DSIF led to chromatin modifications that stabilized TFIID association—a nuance omitted from the simple cartoon in Fig. 7.*

<u>Our response:</u> Also, here we respect the reviewer's concerns. We have now tried to make it clearer in the text and also in our model of Figure 7 that SPT-5 only binds to Pol II in the course of promoter clearance and that it should not be present at promoters during Pol II recruitment. Nevertheless, binding of SPT-5 to Pol II seems to occur prior to reaching the pause site and thus prior to productive elongation, as is evident

from our Supplementary Figure 10a, which shows that SPT-5 starts to associate with chromatin already upstream of the TSS.

We also agree that in our study SPT-5's impact on Pol II recruitment occurs likely through an indirect mechanism. In light of the findings by (Diamant et al., 2016), SPT-5's participation in post-initiation events may lead to a feedback mechanism that alters promoter regions epigenetically, in a way that facilitates Pol II recruitment. We now mention this prior work and hypothesis in the discussion section of the manuscript.

Finally, we substantially altered our model in Figure 7, to be much more simplistic and to keep it void of mechanistic speculations that would interfere with published work.

> *3. This is a minor point, but in the new measurements of phosphatase activity of PP4 complexes towards SPT-5-derived phosphopeptides (Supplementary Fig. 9), there was one such peptide, containing phospho-S671, which could not be dephosphorylated under any of the conditions tested, leaving open the trivial possibility that it is simply not capable of serving as a substrate. Have the authors tried any commercial, non-specific phosphatase (e.g. lambda) on this peptide?*

Our response: Unfortunately, we did not try to dephosphorylate this peptide by use of a non-specific phosphatase. Thus, we cannot conclusively say, if this phosphopeptide is a poor substrate only to PP4 or to phosphatases in general. Nevertheless, we don't think that knowing the answer to this question is crucial for the conclusions that we draw in the manuscript. In any regard we could reliably show that at least some of the identified phosphorylation sites in SPT-5 can be dephosphorylated by $PP4^{SMK-1}$.

> *4. Another minor point: the aforementioned reference to Parua et al. is duplicated (refs. 37 and 38).*

Our response: Thank you for identifying this mistake. We have now corrected this duplication in the new manuscript.

Reviewer #3:

> *In their revised manuscript entitled "DAF-16/FOXO requires Protein Phosphatase 4 to initiate the transcription of stress resistance and longevity promoting genes", Sen et al. satisfactorily addressed all my previous concerns by rephrasing certain statements, including key experimental information that was missing previously, and in some cases, by providing new data. In fact, I applaud the authors for their comprehensive and precise answers/additional explanations. Importantly, the conclusions from the first version of the manuscript remain valid. As indicated previously, the presented mechanistic insights into how the known C. elegans longevity regulator SMK-1 actually exerts its longevity-promoting functions clearly are of interest to the aging field. Accordingly, I now recommend publication in Nature Communications and only have minor comments/suggestions for further improvement:*

Our response: We thank the reviewer for this positive feedback.

- Supplementary figure 9d: the experiment shown is suitable to further support the conclusion of SMK-1 being a PP4-subunit and experimental details are well-described in the methods section; however, to facilitate "reading" this figure, align the label "SMK-1-HA…" horizontally, and not tilted.

Our response: We have now adjusted the labelling in Supplementary Figure 9d accordingly.

- Previous comment on Fig.4a/overlap of up/downregulated gene lists with previous studies: consider including some of this information as a supplementary table or at least indicate that you checked the overlap with other similar studies by a "data not shown" statement. The apparently highly significant overlap with similar studies is indeed encouraging, and my gut feeling is that the reasons cited by the authors (slight differences in culture conditions, genetic background etc) can adequately explain the discrepancies with other studies.

Our response: We have now added information about the overlap of our lists of up/downregulated genes with previous studies to the manuscript, mentioning these overlaps in the results and discussion sections and providing a new Supplementary Table 9 which summarizes these comparisons.

Reviewer #4:

The manuscript is substantially improved.

For ChIP-seq, the issue of Pol II recruitment is solved. The issue of Pol II ChIP signal lateral shift is not. The validated results on Pol II recruitment are sufficient to make a point and the "shift" does not add anything to understanding the mechanism of transcription blockage beyond speculation. To get to the mechanisms requires additional experimentation beyond the scope of this paper. Gro/Pro-seq will not be helpful because the methods are blind to initiating complexes without pre-existing nascent RNA. Anything having to do with lateral shift of ChIP seq peaks should be either deleted completely or moved to discussion as very speculative at best.

Our response: We agree with the reviewer that the defect in Pol II recruitment is much more apparent and robust than any of the defects in promoter clearance or termination of transcription. Thus, we now substantially adjusted the manuscript, to put the focus on defects in Pol II recruitment as the major phenotype arising from lack of SMK-1. Any statements or interpretations related to Pol II shifts in the ChIP-seq data have been either removed or substantially toned down. Furthermore, we tried our best to move the few remaining statements/interpretations of such potential shifts to the discussion section.

Another remaining issue is that introduction into and explanation of DSIF does not occur until the very last section of Results and, when it does, really does not fit in the manuscript as written. Even the title of this Results subsection is rambling. This section needs to be way shortened to focus on actual results instead of introduction and discussion.

Our response: We agree with the reviewer and have now adjusted the manuscript. In fact, we now removed this section entirely from the results, shortened it, and then placed its remnants in the discussion section.

Discussion section is too long and is strongly suggested to be condensed down to the most essential points.

Our response: We now tried our best to make the manuscript more concise. Overall, we shortened the manuscript by nearly 5000 characters. Also, the discussion section we tried to shrink as much as possible. However, this was only partially successful, due to two reasons: 1) As already stated above, we moved the remnants of the last results paragraph to the discussion section. This expanded the discussion a little bit. 2) We found that many parts of the discussion were important and hence should be kept. They put our results into the proper context of the current literature and they address the several apparent open questions and future research directions that arise from our work, which will help the reader to better understand and interpret the importance of our study.

The text may need language editing throughout. The usage of commas appears to be arbitrary. Almost all instances of "eventually" should be deleted. Many "also" words are misplaced within respective sentences.

Our response: We have now edited all but one occurrence of the word "eventually" by either deleting the word or replacing it by a different word or term. Further, we agree that some occurrences of the word "also" were misplaced. We now have repositioned the word where appropriate. Finally, we had a native speaker carefully edit the language and punctuation, leading to many (~200) changes throughout the manuscript.

Line 344. "SPT-5 as the relevant substrate". "The" is too strong a statement that implies an exhaustive/saturating screen, which is not what's been done. "A relevant substrate" appears to be more appropriate.

Our response: We have now changed this sentence accordingly.

Line 537-538. Where are therapeutic interventions coming from? What mechanism would be an interesting (?) therapeutic target, whatever that is. The entire last sentence carries no information and should be deleted.

Our response: We have now deleted this sentence.

Minor points.

Line 271 – 272. Transcription initiation includes Pol II recruitment, so the sentence makes no sense as written.

Our response: We have now changed/corrected the sentence to read "We conclude that $PP4^{SMK\text{-}1}$ is required for transcriptional initiation, predominantly by promoting efficient Pol II recruitment at a subset of DAF-16-activated target genes,...".

Line 330 "letting us disregard ... as relevant substrates". This reads ambiguous: why disregard something that is relevant? Perhaps "exclude" is what was meant.

Our response: Thank you for this suggestion. We have now changed the sentence to read "…, leading us to exclude these SPT family members as relevant substrates.".

Line 355. "Washed it into assay buffer" makes no sense.

Our response: Instead of "washed it into assay buffer" we now write "transferred it to assay buffer", hoping that this better describes the executed step. In fact, we had mis-used the verb "to wash" in multiple similar instances throughout the manuscript and now corrected those other instances, too.

Line 180-181. Can be deleted.

Our response: We have now deleted this sentence.

Line 203. "Successfully regulated". How does one estimate success of regulation? Maybe "properly regulated" was meant.

Our response: Thank you for the suggestion. We have now replaced "successfully" by "properly" in this sentence.

References:

Diamant, G., Bahat, A., and Dikstein, R. (2016). The elongation factor Spt5 facilitates transcription initiation for rapid induction of inflammatory-response genes. Nat. Commun. *7*, 11547.
